# Multi-Slice Radiomic Analysis of Apparent Diffusion Coefficient Metrics Improves Evaluation of Brain Alterations in Neonates With Congenital Heart Diseases

**DOI:** 10.3389/fneur.2020.586518

**Published:** 2020-12-11

**Authors:** Meijiao Zhu, Dadi Zhao, Ying Wang, Qinghua Zhou, Shujie Wang, Xuming Mo, Ming Yang, Yu Sun

**Affiliations:** ^1^Department of Radiology, Children's Hospital of Nanjing Medical University, Nanjing, China; ^2^Institute of Cancer and Genomic Sciences, University of Birmingham, Birmingham, United Kingdom; ^3^Department of Informatics, University of Leicester, Leicester, United Kingdom; ^4^Department of Cardio-Thoracic Surgery, Children's Hospital of Nanjing Medical University, Nanjing, China; ^5^School of Biological Science and Medical Engineering, Southeast University, Nanjing, China

**Keywords:** radiomics, neonate, diffusion weighted imaging, congenital heart disease, neurodevelopment

## Abstract

Apparent diffusion coefficients (ADC) can provide phenotypic information of brain lesions, which can aid the diagnosis of brain alterations in neonates with congenital heart diseases (CHDs). However, the corresponding clinical significance of quantitative descriptors of brain tissue remains to be elucidated. By using ADC metrics and texture features, this study aimed to investigate the diagnostic value of single-slice and multi-slice measurements for assessing brain alterations in neonates with CHDs. ADC images were acquired from 60 neonates with echocardiographically confirmed non-cyanotic CHDs and 22 healthy controls (HCs) treated at Children's Hospital of Nanjing Medical University from 2012 to 2016. ADC metrics and texture features for both single and multiple slices of the whole brain were extracted and analyzed to the gestational age. The diagnostic performance of ADC metrics for CHDs was evaluated by using analysis of covariance and receiver operating characteristic. For both the CHD and HC groups, ADC metrics were inversely correlated with the gestational age in single and multi-slice measurements (*P* < 0.05). Histogram metrics were significant for identifying CHDs (*P* < 0.05), while textural features were insignificant. Multi-slice ADC (*P* < 0.01) exhibited greater diagnostic performance for CHDs than single-slice ADC (*P* < 0.05). These findings indicate that radiomic analysis based on ADC metrics can objectively provide more quantitative information regarding brain development in neonates with CHDs. ADC metrics for the whole brain may be more clinically significant in identifying atypical brain development in these patients. Of note, these results suggest that multi-slice ADC can achieve better diagnostic performance for CHD than single-slice.

## 1. Introduction

Congenital heart disease (CHD) leads to childhood morbidity, affecting six to eight births per million. Among the various types of CHD, non-cyanotic CHDs are relatively common (1). Although recent advancements in cardiac surgery have dramatically increased the survival rates among patients with CHD, the neurodevelopmental and cognitive features of such patients remain controversial (2–4). Clinical studies demonstrated altered pre-surgery brain development in more than half of neonates with CHD—a known a risk factor for neurodevelopmental impairment (5, 6). Long-term studies suggested that these deficits can persist until adolescence and young adulthood, potentially impacting the individual's ability to function in society. Early identification of altered brain development may aid in elucidating the potential mechanisms underlying brain dysmaturation in neonates with CHD, which may in turn allow for timely and appropriate protective treatments (7).

Functional imaging was used to study brain maturity and injury in neonates with CHD (8, 9). For example, magnetic resonance spectroscopy studies showed that neonates with cyanotic CHD exhibit characteristic decreased ratio of *N*-acteyl-aspartate to choline in the thalamus, basal ganglia, and corticospinal tracts (1). Unfortunately, children are often unable to tolerate the lengthy scan durations required for functional imaging, thus its clinical application in pediatric populations becomes less practical.

Conversely, diffusion weighted imaging (DWI) is routinely performed for the non-invasive assessment of tissue cellularity and cellular membrane integrity in clinical settings. DWI detects the random thermal motion of water molecules in living tissue (10–12). Diffusion coefficients of water are expressed as apparent diffusion coefficients (ADC), which reflect water diffusion and the development of membranes in neuronal and glial cells. However, further phenotypic information of lesions remains difficult to obtain through classic methods. Radiomics utilizes the full potential of the ADC to improve clinical diagnosis and prognosis through analysing quantitative factors in brain tissue (13–15). Histogram analysis can be used to quantify the distribution of signal intensity in voxels based on routinely acquired clinical ADC. Lesion heterogeneity is reflected by texture features that describe the statistical interrelationships between adjacent voxels. Recent clinical studies have highlighted the potential of radiomics in aiding the clinical diagnosis of pathologic subtypes of cervical cancer (16) and predicting the molecular characteristics of glioblastoma (17).

To our knowledge, very few studies have investigated radiomic ADC in brain development of neonates with CHDs. Classic methods normally select regions of interest (ROIs) from only representative slices, which may have resulted in underestimation of lesion heterogeneity (18). Alternatively, ROIs can be selected by covering the entire volume of the lesion, allowing for a complete assessment of tissue characteristics and heterogeneity in quantitative analysis (19). This study aimed to investigate the value of single-slice and multi-slice measurements by using ADC metrics and texture features for assessing brain alterations in neonates with CHD.

## 2. Patients and Methods

### 2.1. Participants

This study retrospectively reviewed the preoperative ADC images of neonates with echocardiographically confirmed non-cyanotic CHDs treated at Children's Hospital of Nanjing Medical University from January 2012 to December 2016 (20). Neonates with mild neonatal pneumonia or scalp hematoma exhibiting normal intracranial MRI findings were enrolled as healthy controls (HCs). Seventy patients were excluded due to non-related neurological abnormalities or prematurity. All participants were followed up for 6 months, and those exhibiting typical development of the nervous system were enrolled as HCs. The present study was approved by the ethics committee of our institution (ethic number: 201603005-1).

### 2.2. Image Acquisition

MR images were acquired by using a Siemens Avanto 1.5T scanner (Siemens Healthcare, Erlangen, Germany) and a standard high-definition eight-channel surface head coil. Prior to scanning, participants received 5% chloral hydrate at a dose of 1 mL/kg via oral administration. MRI scans were obtained during sleep, with the patient in the supine position. Axial images were acquired orthogonally to the anterior-posterior commissure line in a standard fashion. Imaging protocols were as follows: multi-planar T_1_-weighted spin-echo (SE) imaging (axial, repetition time (TR) 4,490 ms, echo time (TE) 7.5 ms; sagittal, TR 4,400 ms, TE 9 ms), axial T_2_-weighted fast spin-echo (FSE) imaging (TR 5,570 ms, TE 117 ms), and axial fluid-attenuated inversion recovery (FLAIR) imaging (TR 6,000 ms, TE 92 ms). Axial DWIs were acquired in the Z, Y, and X directions (TR 3,200 ms, TE 99 ms) with *b* values of 0 and 1,000 s/mm^2^. ADC images were automatically processed using a standard mono-exponential fit.

### 2.3. Feature Extraction

Single axial brain slices were selected at the level of periventricular white matter injury or periventricular leukomalacia, considering (punctate) white matter injury, periventricular leukomalacia, and stroke are the most commonly observed lesions on MRI (18, 21). All CHD patients were assessed through MR images by experienced neurologists to confirm having no obvious brain damages. Since the ROIs containing the entire tumor in each slice of the ADC image could provide overall information related to the tumor (22), ROIs were drawn on single-slice and multi-slice images by experienced neuro-radiologists for comparison after excluding cerebrospinal fluid outside the brain. ADC metrics and texture features were extracted for only regions of interest, which include average ADC, minimum ADC, maximum ADC, peak location of ADC (mode), ADC skewness (asymmetry), ADC kurtosis (flatness), ADC entropy (randomness), ADC variance, the 5th–95th percentiles ADC, contrast, dissimilarity, homogeneity, angular second moment (ASM), and energy (23–25). Computational feature extraction was performed by using Python (version 2.7, Python Software Foundation, Delaware, United States).

### 2.4. Statistical Analysis

The Kruskal-Wallis test was performed to compare the gender. The non-parametric unpaired *t*-test was performed to compare the ages and gestational ages. The Pearson correlation analysis was performed to evaluate the correlations between ADC metrics and gestational age. The independent-samples *t*-test was performed to compare group-specific ADC metrics and texture features. Receiver operating characteristic (ROC) analysis based on the empirical method was performed to evaluate the diagnostic performance of ADC metrics for CHDs (26), where both the area under the curve (AUC) and the Youden index value (YIV) were analyzed. All statistical analyses were performed by using R (version 3.6.2, R Foundation, Vienna, Austria).

## 3. Results

### 3.1. Demographic Characteristics

**Figure 1:**
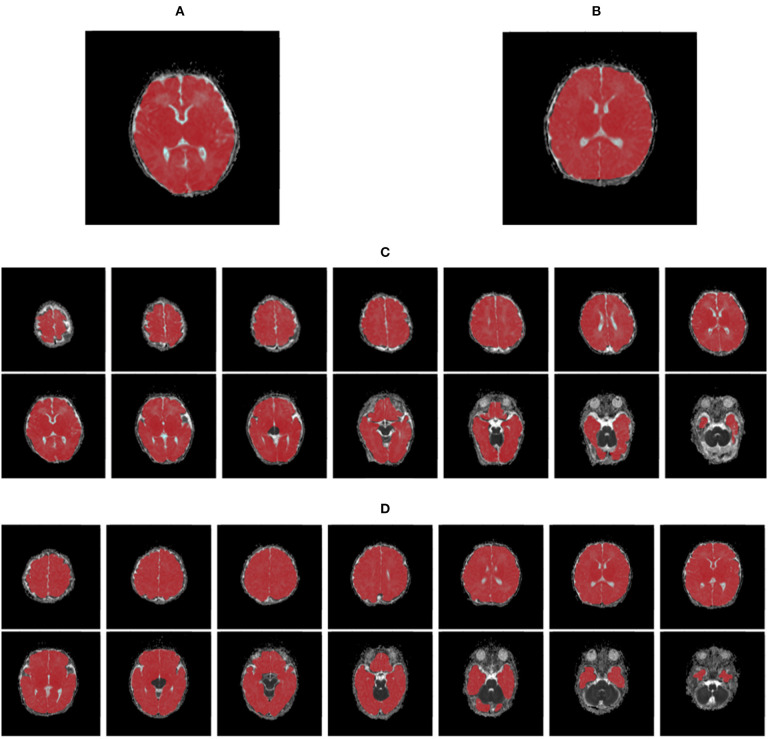
The maps of apparent diffusion coefficients in single- **(A,B)** and multi-slice **(C,D)** diffusion weighted images for a neonate with congenital heart disease **(A,C)** and a health control **(B,D)**, where regions of interest are highlighted in red.

**Figure 2:**
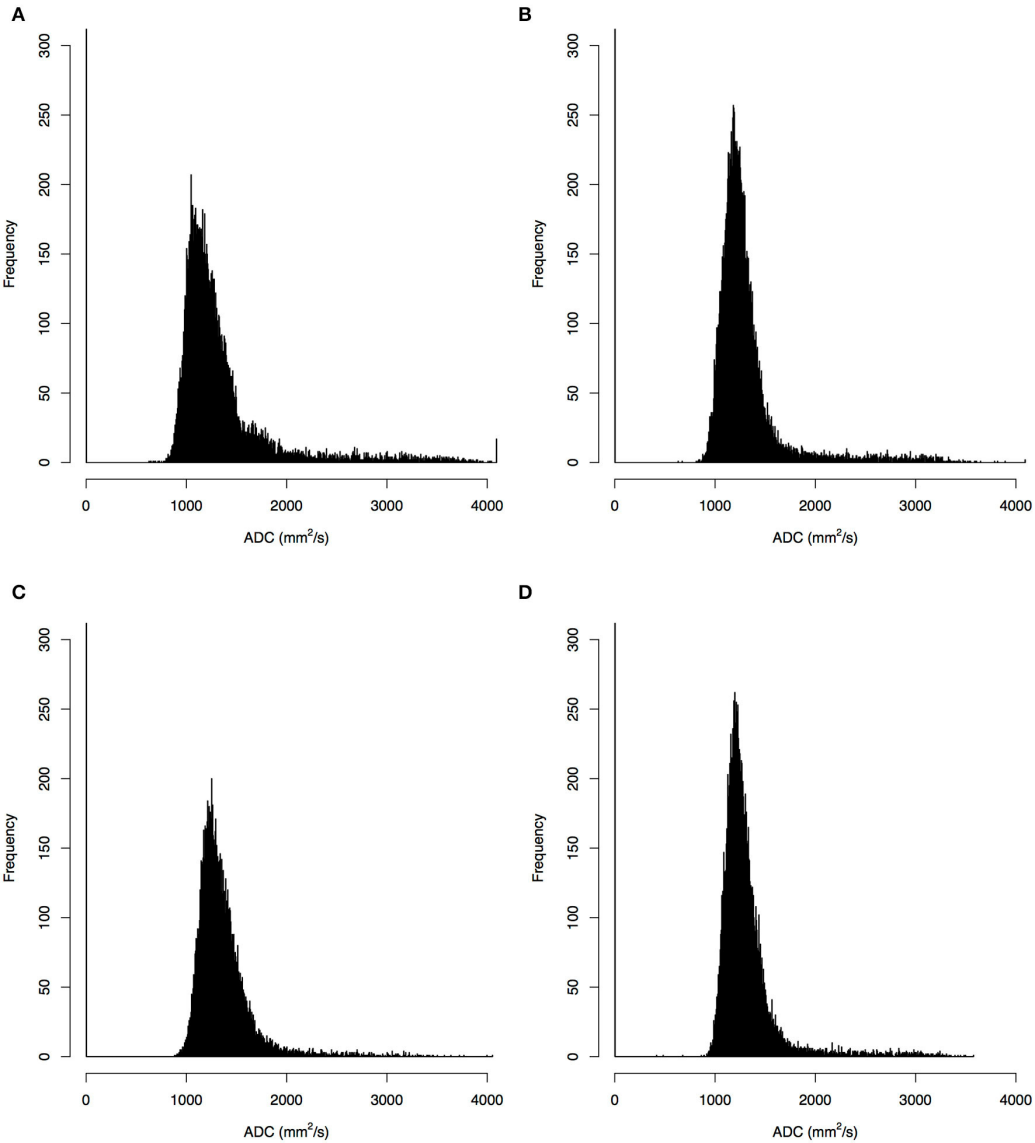
Histograms of apparent diffusion coefficients (ADC) in the single- **(A,B)** and multi-slice **(C,D)** diffusion weighted images for a neonate with congenital heart diseases **(A,C)** and a health control **(B,D)**, where the histograms for multi-slice imaging were given by a selected slice.

Sixty patients with CHDs [38 boys and 22 girls; age mean 10.5 days, standard deviation (SD) 2.91 days] and 22 HCs (14 boys and eight girls; age mean 8.6 days, SD 4.31 days) were enrolled in the cohort (Table 1). The malignant cohort included those with atrial septal defects (*N* = 38), patent ductus arteriosus (*N* = 1), both atrial and ventricular septal defects (*N* = 2), ventricular septal defect and patent ductus arteriosus (*N* = 1), atrial septal defects and patent ductus arteriosus (*N* = 18). The distribution of baseline epidemiological characteristics (gender *P* = 0.98, age *P* = 0.07, gestational age *P* = 0.11) was balanced between the CHD and HC groups. Representative ADC images and normalized histogram patterns for each group are presented in Figures 1, 2. Visual assessment revealed differences in the skewness and kurtosis of the histograms for the CHD and NC groups. Skewness was greater in the HC group than in the CHD group, reflected by greater deviation from a normal distribution, while bias was greater among the cohort cases. Kurtosis, which reflects the sharpness of a frequency-distributed curve, more closely approximated a normal distribution in the CHD group than in the HC group.

**Table 1:** Demographic variables.

	**Neonates with congenital**	**Health Controls**	***P***
	**Heart disease**		
Gender (female:male)	22:38	8:4	0.98
Age (days)	10.5 ± 2.9	8.6 ± 4.2	0.07
Gestational age (weeks)	40.6 ± 1.4	40.0 ± 1.3	0.11

### 3.2. Correlation Between Features and Gestational Age

**Figure 3:**
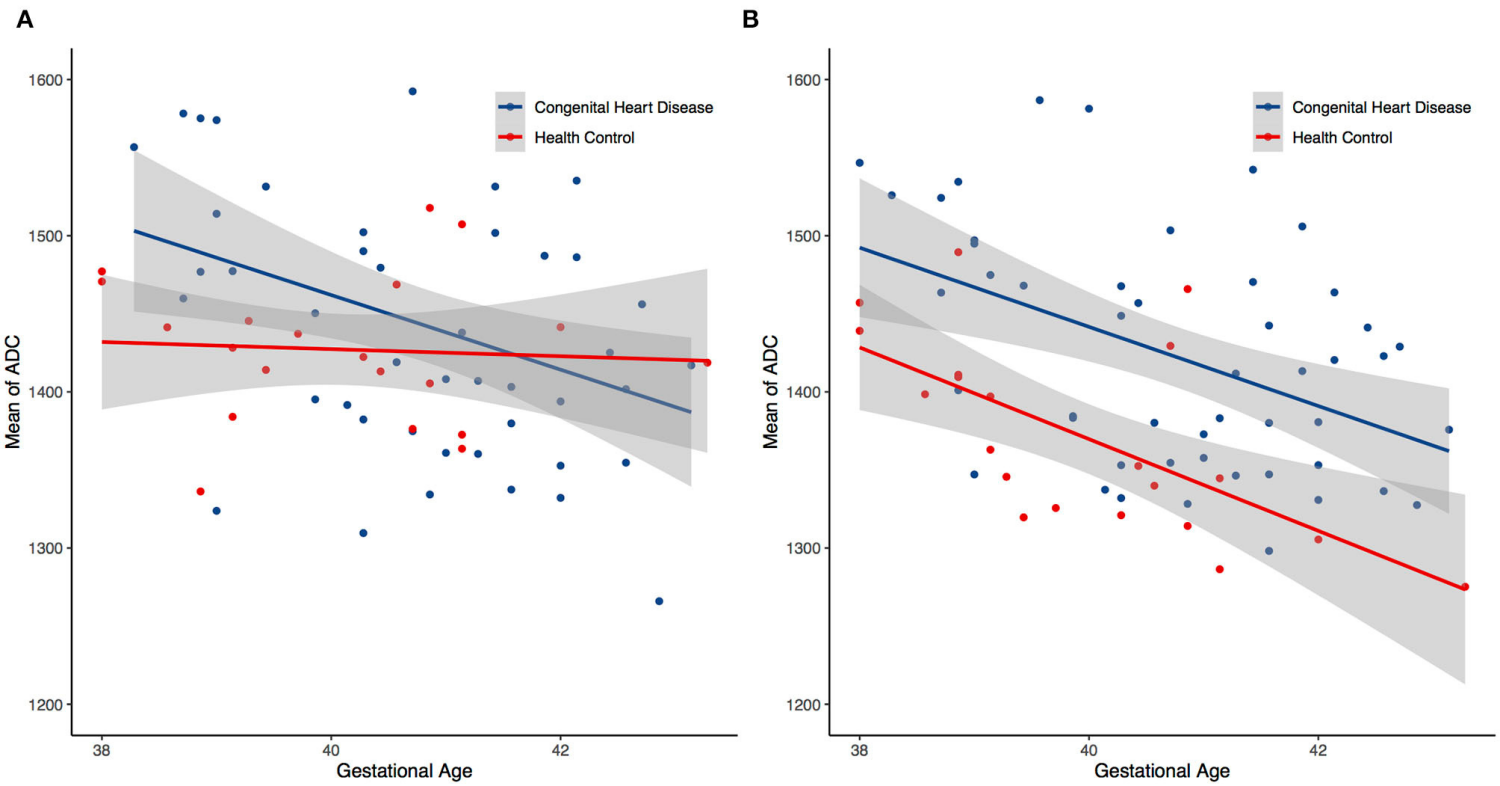
Scatter plots showing negative trend between the gestational age and the mean apparent diffusion coefficients (ADC) for both single- **(A)** and multi-slice **(B)** diffusion weighted imaging.

All ADC of the CHD and HC groups were significantly and inversely correlated with gestational age (*r* < 0, *P* < 0.05) in both single- and multi-slice analyses (Figure 3). In the CHD group, all single- and multi-slice measurements exhibited a significant inverse correlation with gestational age (*r* < 0, *P* < 0.05), except for minimum ADC (*r* > 0, *P* > 0.05). However, in the HC group, no measurements exhibited a significant inverse correlation with gestational age (*r* < 0, *P* > 0.05), except for average ADC (*r* < 0, *P* < 0.05).

### 3.3. Comparison of Single and Multi-Slice Measurements

**Figure 4:**
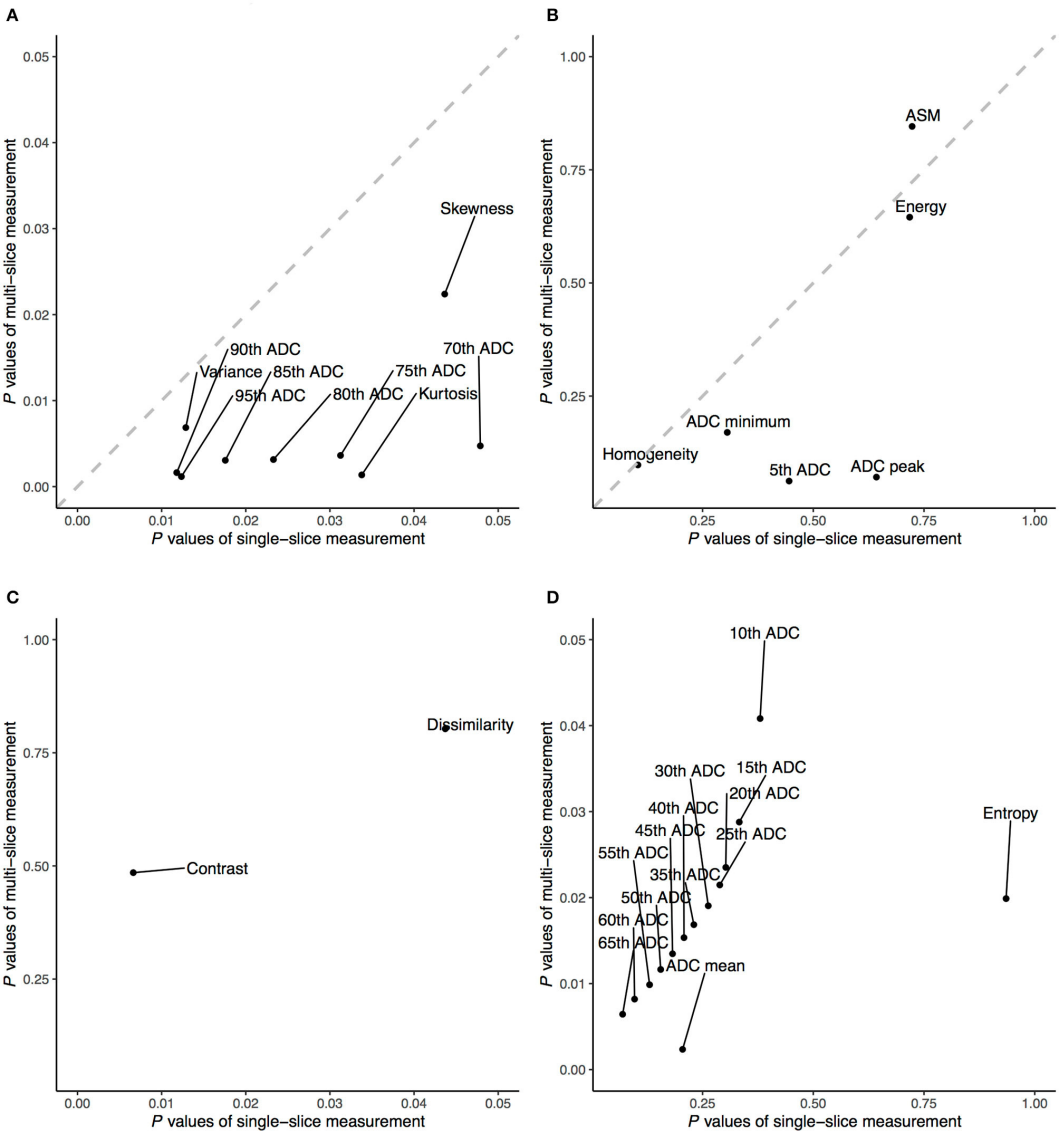
Comparison of the significance level of apparent diffusion coefficients (ADC) and texture features between single- and multi-slice diffusion weighted imaging in evaluating neonates with congenital heart diseases compared to health controls, given as significant **(A)** and insignificant **(B)** in both single- and multi-slice measurements as well as significant for only single- **(C)** or multi-slice **(D)** measurements.

ADC metrics were higher in the CHD group than in the HC group. The significance of ADC metrics is shown in Figure 4A, which shows that certain ADC measurements allowed for significant discrimination of the CHD and HC groups. Figure 4B illustrates that a few ADC metrics show no significant differences between the two groups. Such measurements included the 70th–95th percentile ADC for the single-slice analyses as well as the 10th–95th percentile ADC for the multi-slice analyses. For both single- and multi-slice ADC analyses, kurtosis, variance, and skewness allowed for significant discrimination between the CHD and HC groups. Additional factors including ADC entropy and average ADC allowed for discrimination in the multi-slice analysis (*P* < 0.05, Figure 4D). Skewness and kurtosis were lower in the CHD group than in the HC group, in accordance with the results of the visual assessment. Multi-slice DWI revealed higher entropy and a more randomly distributed histogram in the HC group than in the CHD group, suggestive of greater ADC variability.

### 3.4. Comparison of ADC Metrics and Texture Features

Unlike the ADC metrics, most texture features showed no significance between the CHD and HC groups in either the single- or multi-slice analyses (*P* > 0.05), including contrast, dissimilarity, homogeneity, ASM, and energy. However, some texture features allowed for significant discrimination of the CHD and HC groups in single-slice measurements (*P* < 0.05, Figure 4C).

### 3.5. Diagnostic Performance

**Figure 5:**
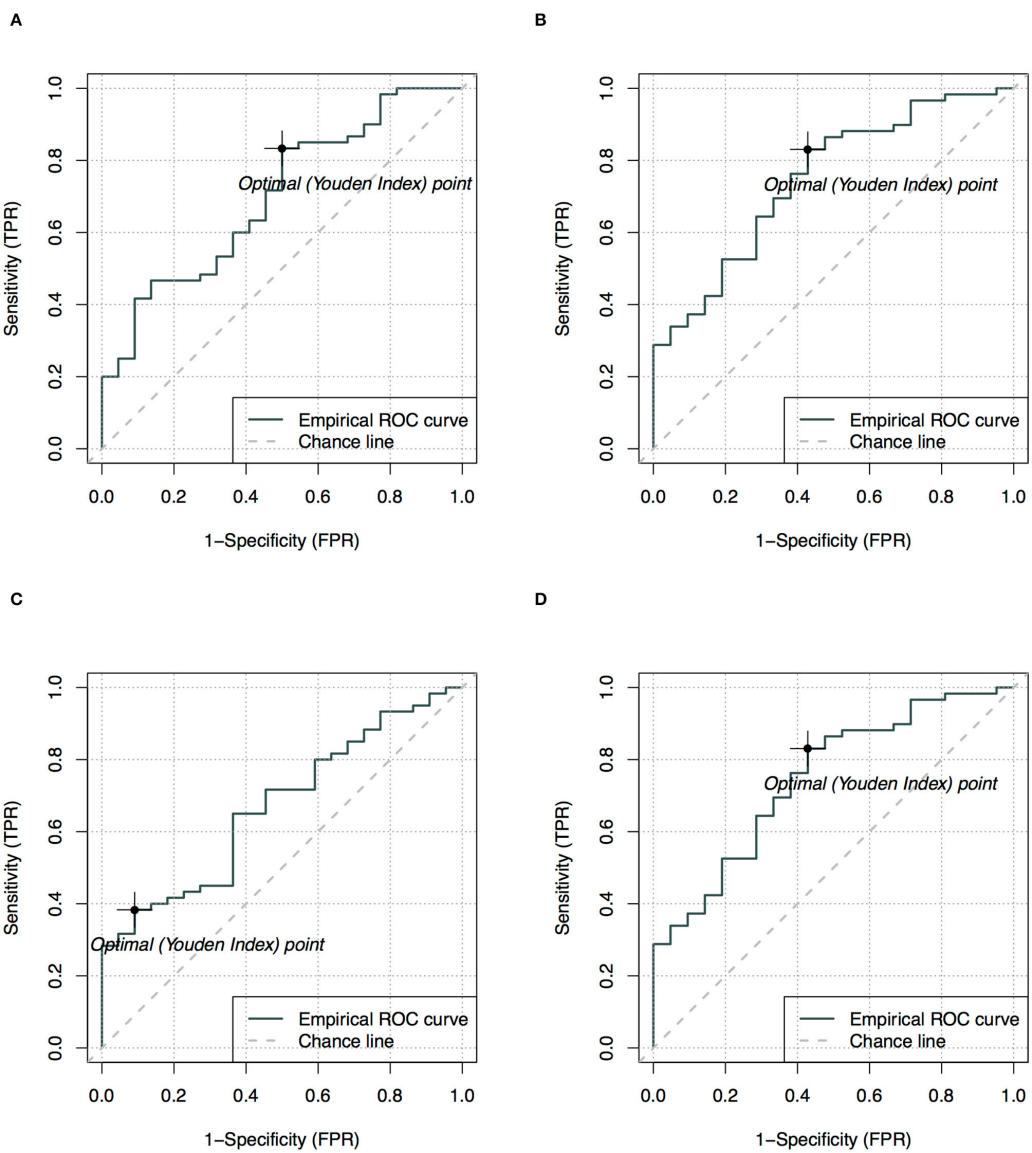
Receiver operating characteristics analysis with optimal points for the 90th **(A,B)** and 95th **(C,D)** apparent diffusion coefficients (ADC), comparing single-slice measurement **(A,C)** and multi-slice measurement **(B,D)** in evaluating neonates with congenital heart diseases compared to health controls.

**Figure 6:**
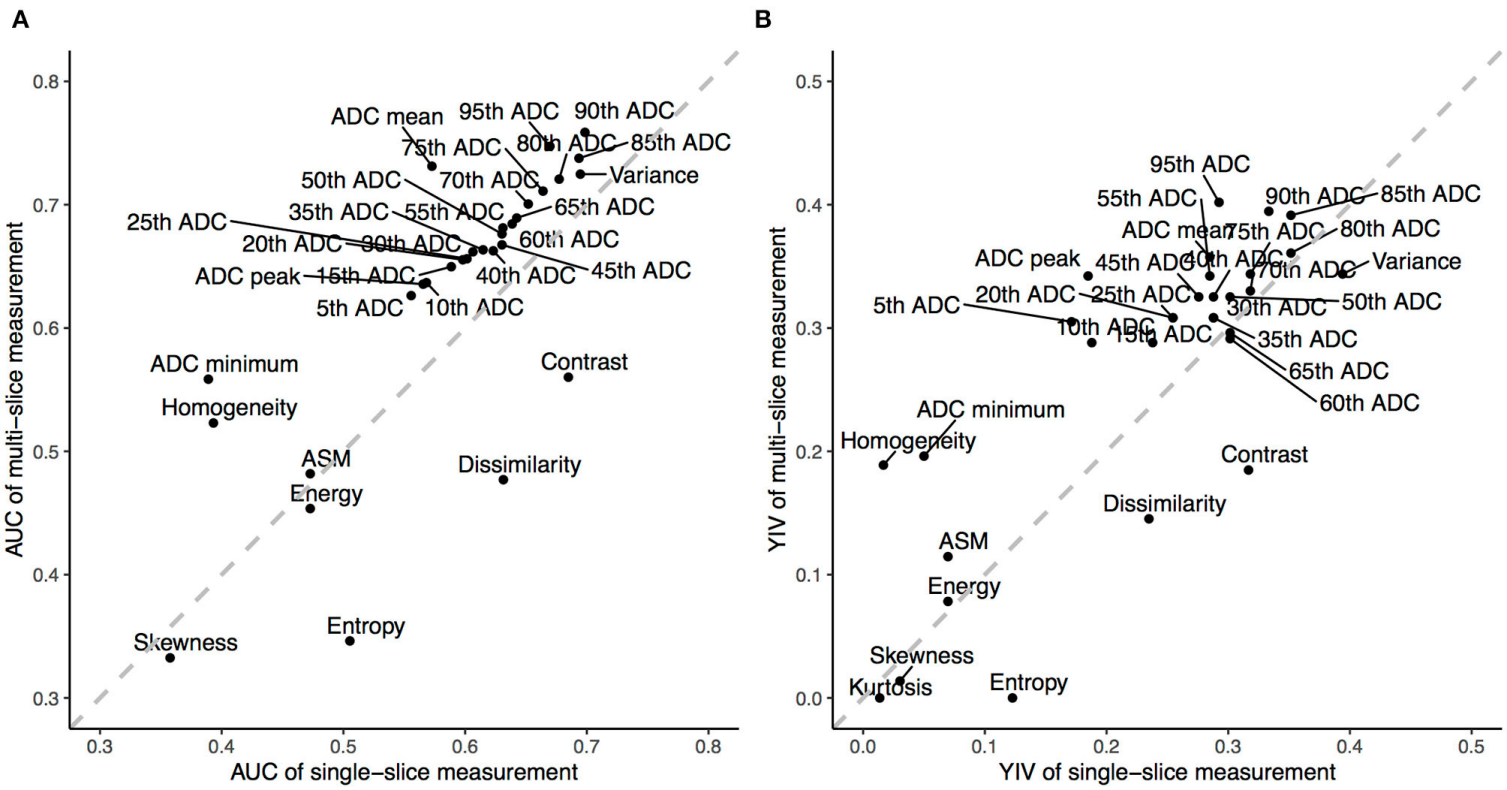
The Area Under the Curve (AUC) and Youden Index Values (YIV) of the apparent diffusion coefficients (ADC) and texture features estimated through receiver operating characteristics for single- **(A)** and multi-slice **(B)** diffusion weighted imaging in evaluating neonates with congenital heart diseases compared to health controls.

Among all ADC metrics and texture features, the 90th (single-slice: AUC = 0.70, YIV = 0.33, Figure 5A; multi-slice, AUC = 0.76, YIV = 0.39, Figure 5B) and 95th percentile ADC (single-slice: AUC = 0.67, YIV = 0.29, Figure 5C; multi-slice: AUC = 0.75, YIV = 0.40, Figure 5D) values allowed for the greatest discrimination between the CHD and HC groups (Figure 6). ROC analysis of ADC metrics suggested that multi-slice measurements exhibited generally greater diagnostic capabilities than single-slice measurements, except for ADC entropy, skewness, and kurtosis as well as the 60th and 65th percentile ADC. ROC analysis also suggested that texture features were less significant for discrimination than most ADC metrics (Figure 6). ASM and homogeneity measurements were more significant for discrimination in multi-slice analyses than in single-slice analyses, while contrast and dissimilarity were less significant.

## 4. Discussion

Changes in diffusion in the white matter of the perinatal brain (which may be related to myelination) could be a function of gestational age (27). This study showed similar univariate associations between several ADC metrics and the gestational age. Scatter plots of both measurements (Figure 3) revealed a linear decline in regional ADC metrics as a function of the gestational age in both the CHD and HC groups. This corresponds to the negative correlation between brain diffusion measurements and gestational age reported before (28). These results indicate that radiomics can be used to quantitatively assess the extent of cerebral myelination in neonates.

In this study, ADC parameters were higher in the CSD group than in the HC group, suggestive of a regional increase in water diffusion and higher local free water content in the CSD group (29). Such differences may be caused by chronic hypoxic ischemic injury due to impaired circulation of oxygenated blood in the fetal and neonatal brain in the CHD group. Mild hypoxia-ischemia is reported to result in selective death and arrested maturation of oligodendrocyte progenitors, possibly leading to delays in brain myelination (21). In addition, delayed brain myelination in children with CHDs may lead to immature development of neurons and glial cells (30, 31). Taken together, these findings suggest that impaired myelination of axons in neonates with CHD can result in increased ADC parameters.

The analysis of ADC metrics demonstrated the heterogeneity of the CHD and HC groups. Indeed, ADC metrics revealed significant differences between the CHD and HC groups for single- and multi-slice analyses. Furthermore, values at the higher end of the cumulative ADC metrics significantly differed between the two groups, and the ADC histograms exhibited divergence toward the higher end, suggesting that the difference between the two groups is mostly observed at higher ADC. Ninetieth percentile ADC represented the most stable parameter for discriminating the CHD and HC groups, in accordance with the findings of a previous study that aimed to discriminate between radiological indeterminate vestibular schwannoma and meningioma in the cerebellopontine angle (32). Such phenomenons were suggested to be potentially caused by the greater frequency of necrotic or cystic components in high-grade or malignant tumors (18, 33). Similarly, the results in this study may be associated with increased water content in the brain, as reflected by higher ADC and delayed myelination, in children with CHD.

ADC histograms may be a potential biomarker of alterations of tissue structure. The lower skewness and kurtosis observed in the CHD group may reflect an abundance of cystic or edematous tissue, suggesting that lesions can be characterized physiologically by assessing the curvature of ADC histograms (13, 34). Further studies are required to clarify its classification performance between CHD and HC groups and validate its application in clinical settings.

This study indicated that the selected texture features were unable to achieve better diagnostic performance than ADC metrics. This may be due to substantial overlap in the regional ADCs for whole slices or whole brains that might lead to a lack of significant differences in texture features. Recent advances combined ADC metrics and texture features to perform classification for high-risk atypical meningiomas (35). Compared to ADC metrics that showed significance, texture features was suggested to have limited contribution to tumor classification. This finding indicates the limited ability of texture features for characterizing brain lesion. In summary, ADC metrics may aid in the early assessment of brain alterations in neonates with CHD.

To the best of our knowledge, this is the first study comparing the diagnostic performance of ADC metrics between single-slice and multi-slice measurements. One of the most common types of brain injury seen in these neonates is white matter injury, which is similar to the pattern of injury observed in preterm neonates (36). Severe hypoxia-ischemia, presenting in the form of periventricular leukomalacia, results in neuronal death and could contribute to a reduction in axonal density (2). Single layers of periventricular white matter are therefore chosen and considered representative. Multi-slice measurements provided more statistically significant ADC parameters for diagnosis than single-slice measurements. Such multi-slice measurements may reflect the diffuse state of the brain in neonates with CHD more accurately than single-slice measurements, which is potentially due to the structural complexity of the brain and differences in the distribution of water molecule diffusion in the whole brain. ADC grayscale values and distributions derived from whole tumors could provide more quantitative information regarding the characteristics and heterogeneity of the tissue (15, 32), further supporting the potential clinical use of multi-slice measurements. Therefore, preoperative evaluation of the ADC using multiple slices may provide more useful information for differentiating patients with CHD from HCs. Meanwhile, given that single-slice measurements are easier to perform, the optimal technique should be determined based on individual patient needs in clinical settings. More detailed studies of functional areas will be carried out combined with following up.

The present study possesses some limitations of note. Firstly, performing MRI in healthy neonates is challenging because they are not normally included in clinical imaging studies. Consequently, few studies have reported ADC for healthy neonates, making it difficult to compare our findings with those of previous researchers (27). In addition, all neonates in our HC group were considered *healthy* based on MRI findings, but this grouping method also included patients with mild neonatal pneumonia or scalp hematoma. Nonetheless, all patients in the HC group were followed up for 6 months to confirm that no subsequent brain diseases occurred. Secondly, our study was also limited by its cross-sectional design, which did not allow us to obtain follow-up data regarding the relationship between ADC metrics and the development of advanced neural function. However, our preliminary results suggest that ADC metrics can be used to identify patients with CHDs, highlighting the need for additional validation studies. Lastly, the influence of genomic characteristics were not included due to the challenges associated with collecting such data. Future studies with extended follow-up periods should therefore assess the impact of genetic characteristics on neurodevelopmental disorders in children with CHDs.

## 5. Conclusion

Multi-slice analysis of the whole brain may provide more significant ADC metrics for the diagnosis of CHDs in neonates than single-slice analysis. Clinical application of multi-slice ADC may allow for more accurate diagnosis of CHDs than visual assessments based on single-slice measurements or structural imaging.

## Data Availability Statement

The original contributions presented in the study are included in the article/Supplementary Materials, further inquiries can be directed to the corresponding author/s.

## Ethics Statement

The studies involving human participants were reviewed and approved by Ethics Committee, children's Hospital of Nanjing Medical University. Written informed consent to participate in this study was provided by the participants' legal guardian/next of kin.

## Author Contributions

MZ, YW, and SW prepared the data. MZ performed the literature search, analyzed the results, and drafted the manuscript. DZ analyzed the data, performed statistical analysis, and contributed substantially to manuscript revision. QZ preliminarily corrected the language. MY, XM, and YS supervised manuscript development and reviewed all sections. All authors reviewed and approved the final version of the manuscript.

## Conflict of Interest

The authors declare that the research was conducted in the absence of any commercial or financial relationships that could be construed as a potential conflict of interest.
