# Supplementary material for: Multi-Slice Radiomic Analysis of Apparent Diffusion Coefficient Metrics Improves Evaluation of Brain Alterations in Neonates With Congenital Heart Diseases
Source: Front Neurol. 2020 Dec 11;11:586518. doi: 10.3389/fneur.2020.586518 (PMC7759540; doi:10.3389/fneur.2020.586518)
Supplement: Supplementary file 1 [file Data_Sheet_1.pdf]

Supplementary Material

1 SUPPLEMENTARY FIGURES

- Correlation with gestational age for all features including
  - Apparent diffusion coefficients (ADC)
    - The 5th percentile ADC (Figure S1)
    - The 10th percentile ADC (Figure S2)
    - The 15th percentile ADC (Figure S3)
    - The 20th percentile ADC (Figure S4)
    - The 25th percentile ADC (Figure S5)
    - The 30th percentile ADC (Figure S6)
    - The 35th percentile ADC (Figure S7)
    - The 40th percentile ADC (Figure S8)
    - The 45th percentile ADC (Figure S9)
    - The 50th percentile ADC (Figure S10)
    - The 55th percentile ADC (Figure S11)
    - The 60th percentile ADC (Figure S12)
    - The 65th percentile ADC (Figure S13)
    - The 70th percentile ADC (Figure S14)
    - The 75th percentile ADC (Figure S15)
    - The 80th percentile ADC (Figure S16)
    - The 85th percentile ADC (Figure S17)
    - The 90th percentile ADC (Figure S18)
    - The 95th percentile ADC (Figure S19)
    - Mean ADC (Figure S20)
    - Minimum ADC (Figure S21)
    - Maximum ADC (Figure S22)
  - Texture features
    - Angular second moment (ASM) (Figure S23)
    - Contrast (Figure S24)
    - Dissimilarity (Figure S25)
    - Energy (Figure S26)
    - Entropy (Figure S27)
    - Homogeneity (Figure S28)
    - Kurtosis (Figure S29)
    - Skewness (Figure S30)
    - Variance (Figure S31)

- Diagnostic ability through receiver operating characteristics for all features including
  - ADC
    - The 5th percentile ADC (Figure S32)
    - The 10th percentile ADC (Figure S33)
    - The 15th percentile ADC (Figure S34)
    - The 20th percentile ADC (Figure S35)
    - The 25th percentile ADC (Figure S36)
    - The 30th percentile ADC (Figure S37)
    - The 35th percentile ADC (Figure S38)
    - The 40th percentile ADC (Figure S39)
    - The 45th percentile ADC (Figure S40)
    - The 50th percentile ADC (Figure S41)
    - The 55th percentile ADC (Figure S42)
    - The 60th percentile ADC (Figure S43)
    - The 65th percentile ADC (Figure S44)
    - The 70th percentile ADC (Figure S45)
    - The 75th percentile ADC (Figure S46)
    - The 80th percentile ADC (Figure S47)
    - The 85th percentile ADC (Figure S48)
    - The 90th percentile ADC (Figure S49)
    - The 95th percentile ADC (Figure S50)
    - Mean ADC (Figure S51)
    - Minimum ADC (Figure S52)
    - Maximum ADC (Figure S53)
  - Texture features
    - Angular second moment (ASM) (Figure S54)
    - Contrast (Figure S55)
    - Dissimilarity (Figure S56)
    - Energy (Figure S57)
    - Entropy (Figure S58)
    - Homogeneity (Figure S59)
    - Kurtosis (Figure S60)
    - Skewness (Figure S61)
    - Variance (Figure S62)

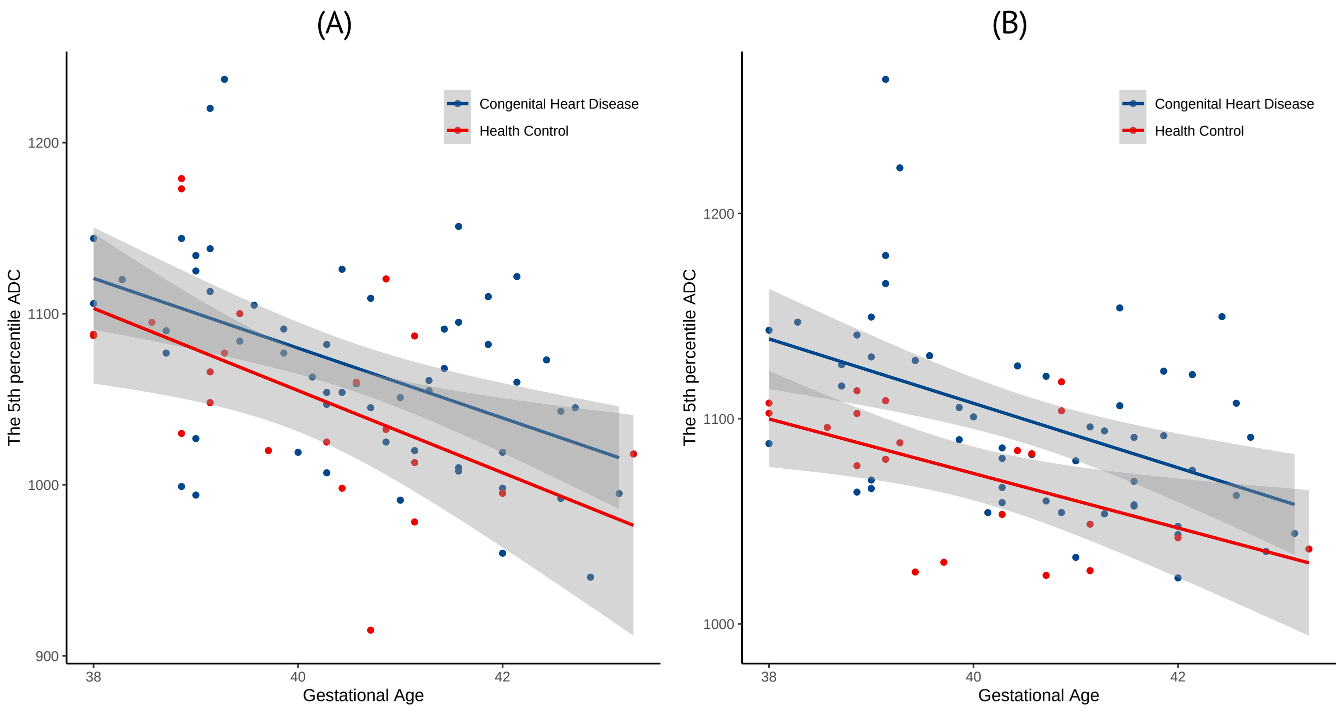

Figure S1. Regression analysis for the 5th percentile apparent diffusion coefficients with gestational age, comparing single- (A) and multi-slice (B) diffusion weighted imaging.

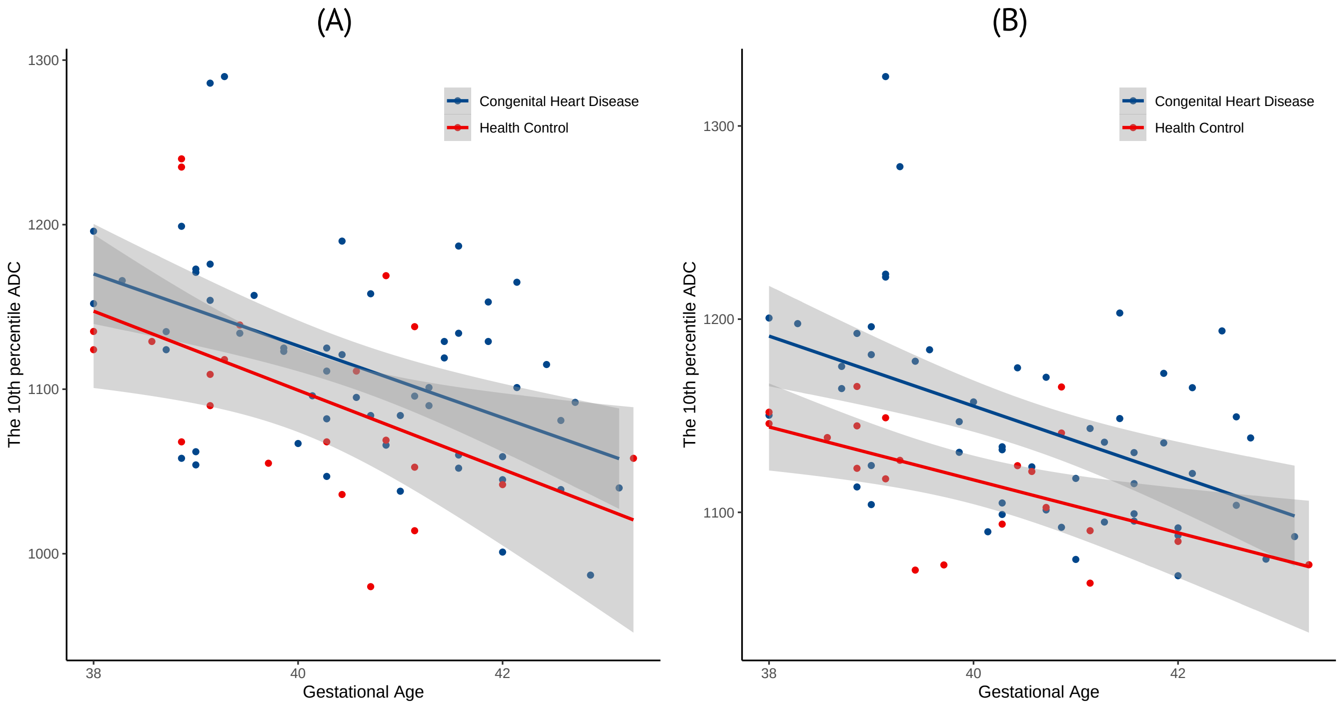

Figure S2. Regression analysis for the 10th percentile apparent diffusion coefficients with gestational age, comparing single- (A) and multi-slice (B) diffusion weighted imaging.

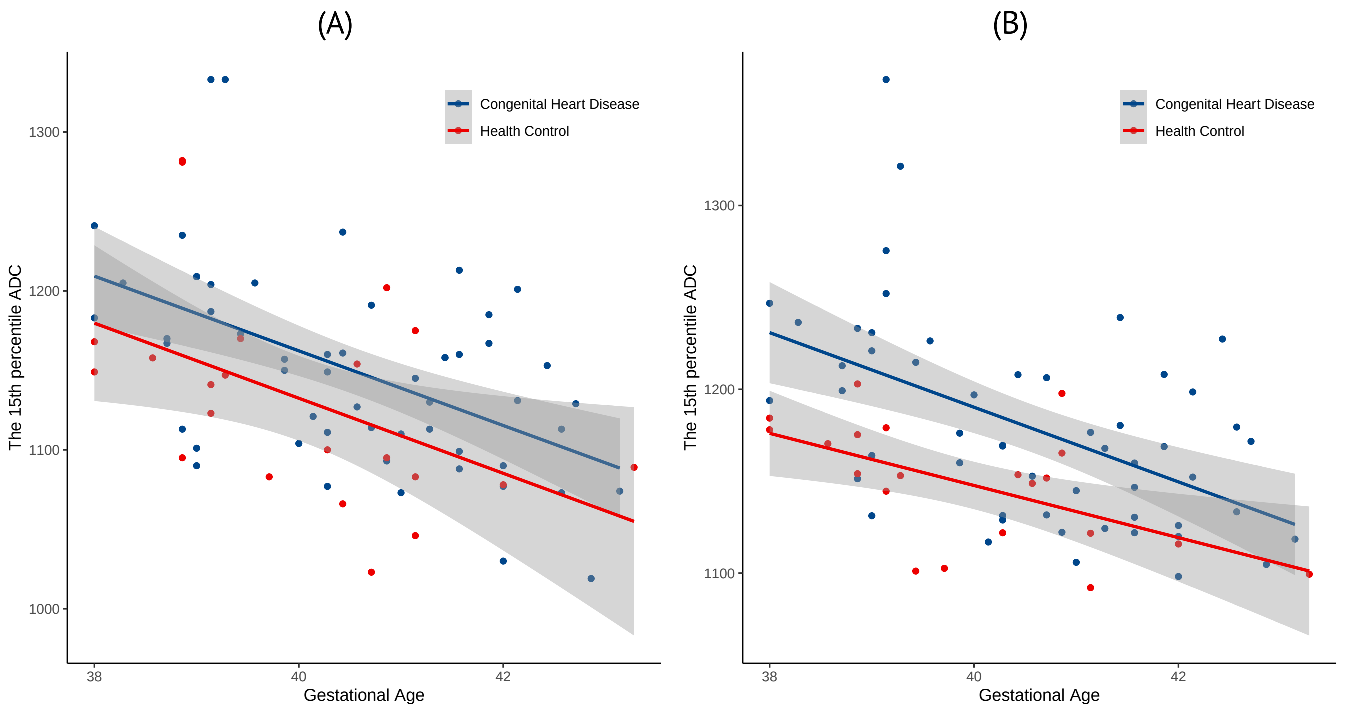

Figure S3. Regression analysis for the 15th percentile apparent diffusion coefficients with gestational age, comparing single- (A) and multi-slice (B) diffusion weighted imaging.

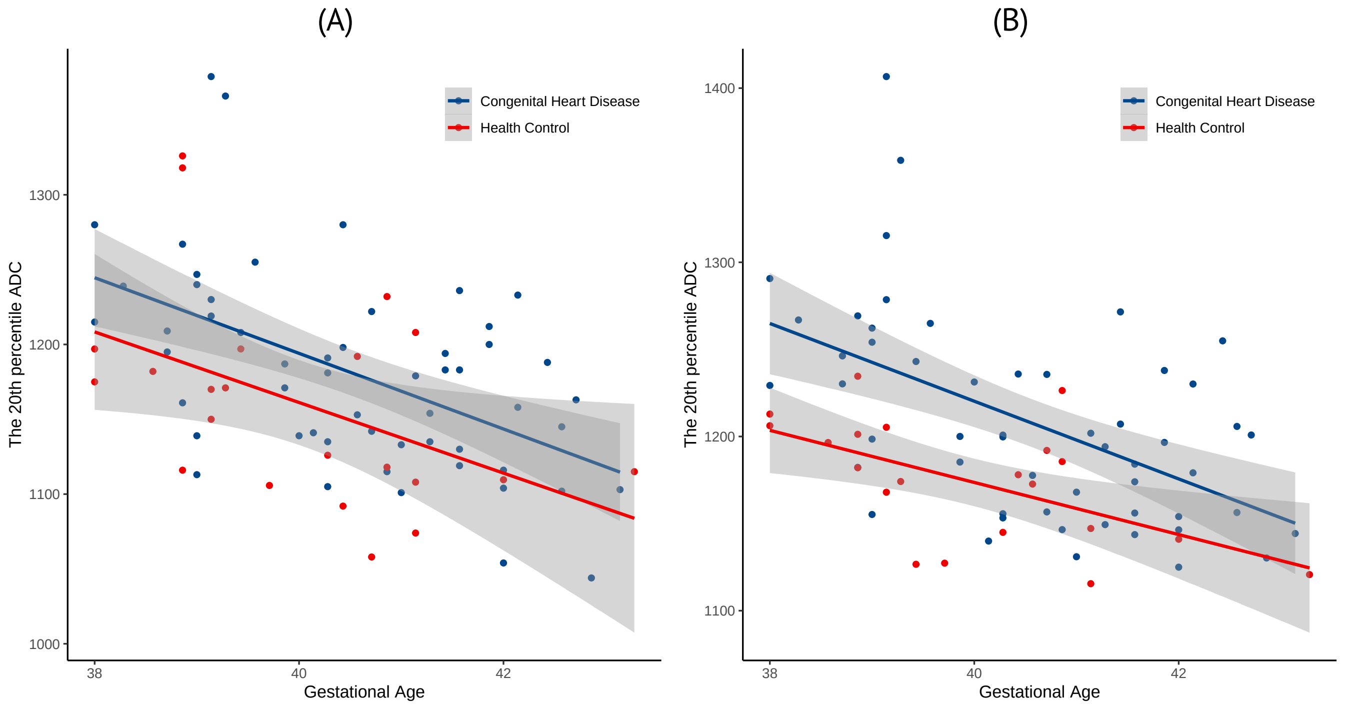

Figure S4. Regression analysis for the 20th percentile apparent diffusion coefficients with gestational age, comparing single- (A) and multi-slice (B) diffusion weighted imaging.

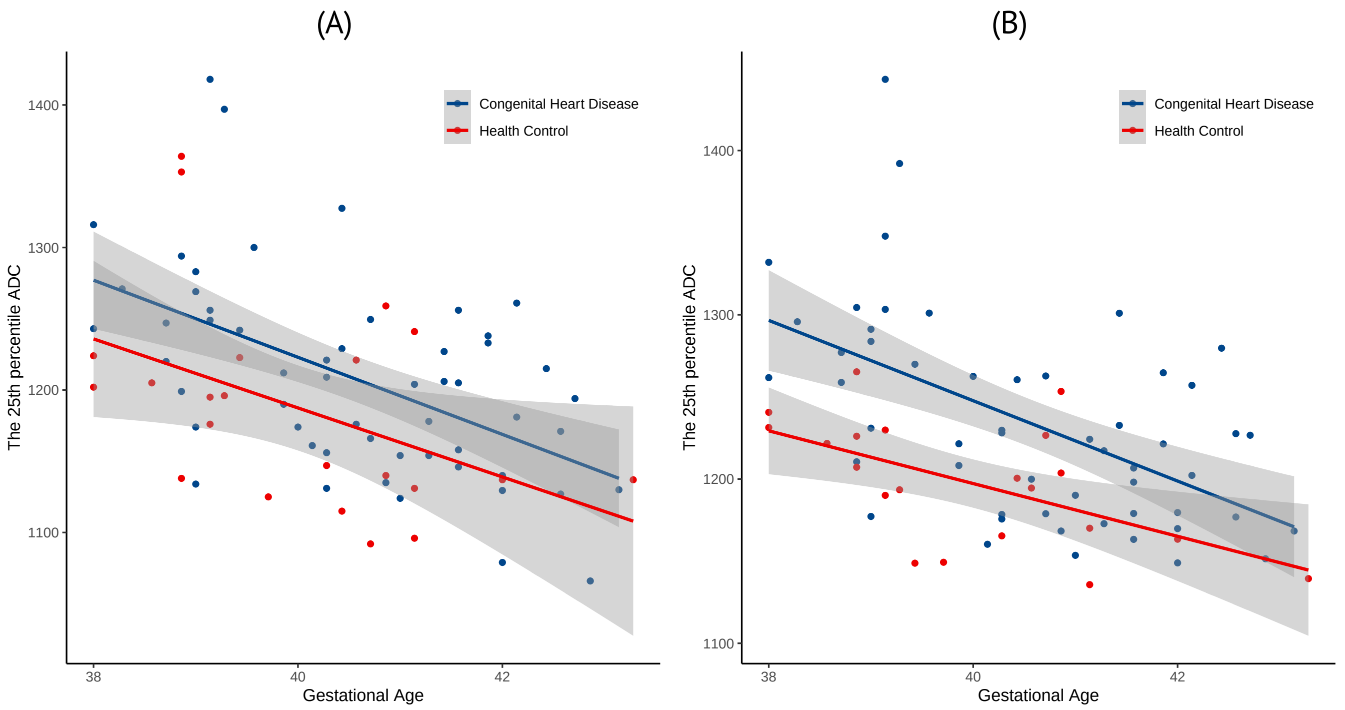

Figure S5. Regression analysis for the 25th percentile apparent diffusion coefficients with gestational age, comparing single- (A) and multi-slice (B) diffusion weighted imaging.

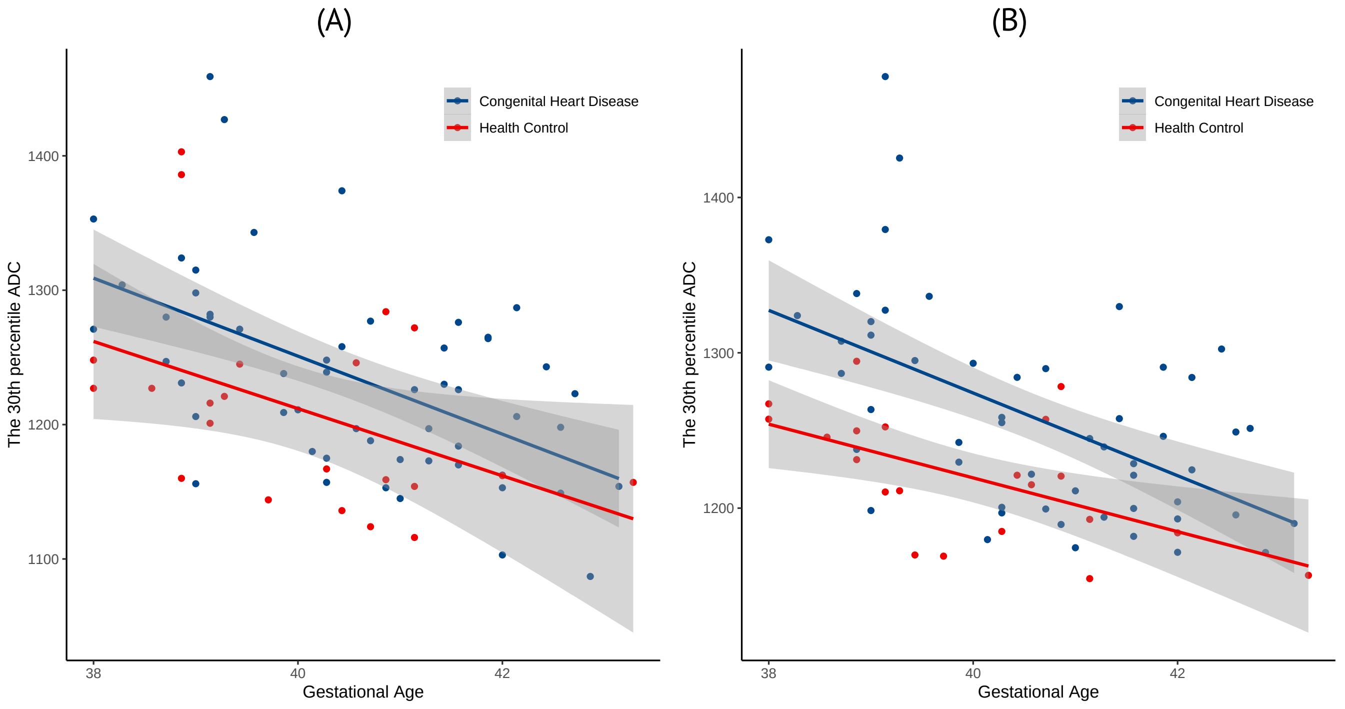

Figure S6. Regression analysis for the 30th percentile apparent diffusion coefficients with gestational age, comparing single- (A) and multi-slice (B) diffusion weighted imaging.

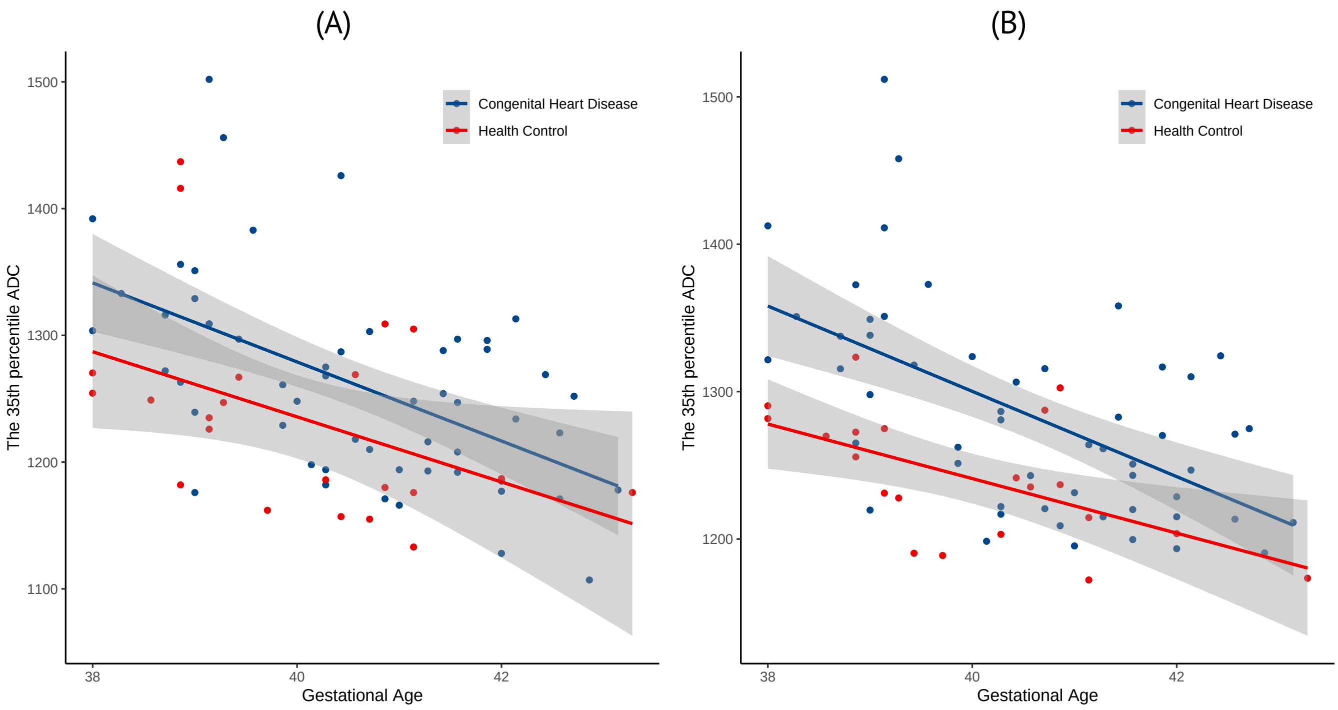

Figure S7. Regression analysis for the 35th percentile apparent diffusion coefficients with gestational age, comparing single- (A) and multi-slice (B) diffusion weighted imaging.

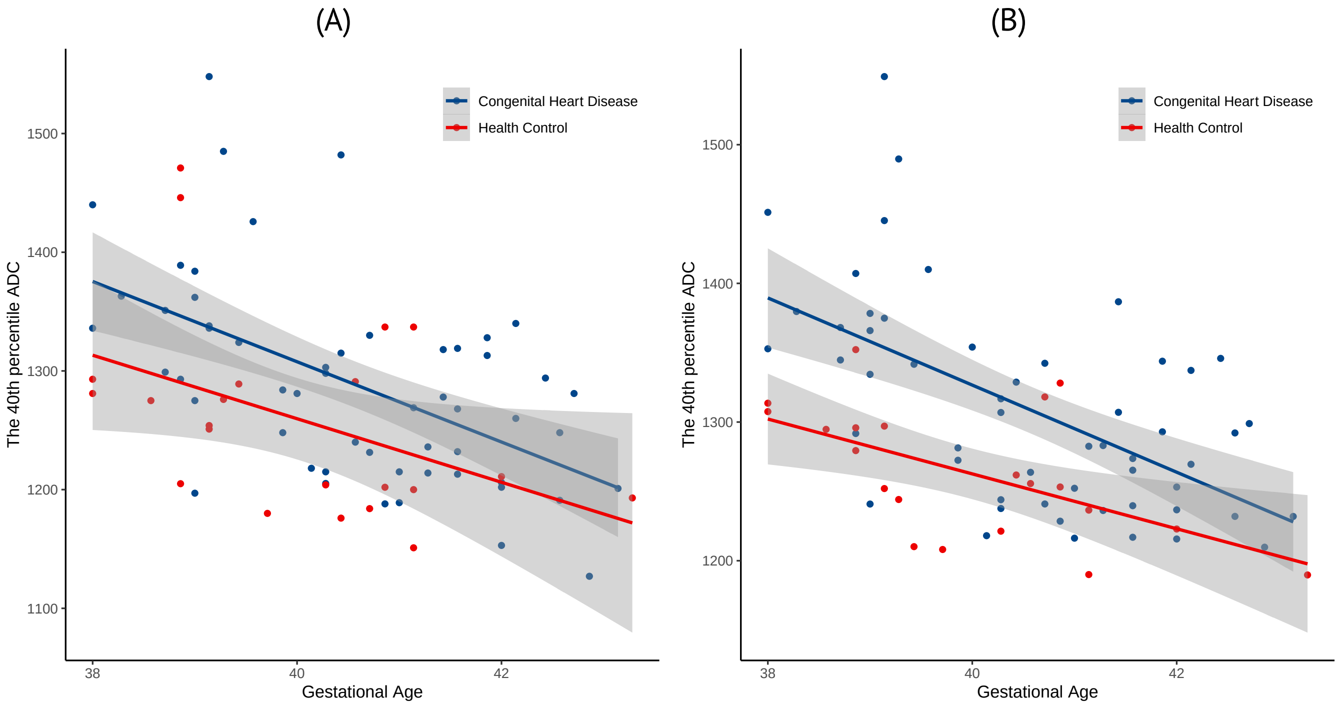

Figure S8. Regression analysis for the 40th percentile apparent diffusion coefficients with gestational age, comparing single- (A) and multi-slice (B) diffusion weighted imaging.

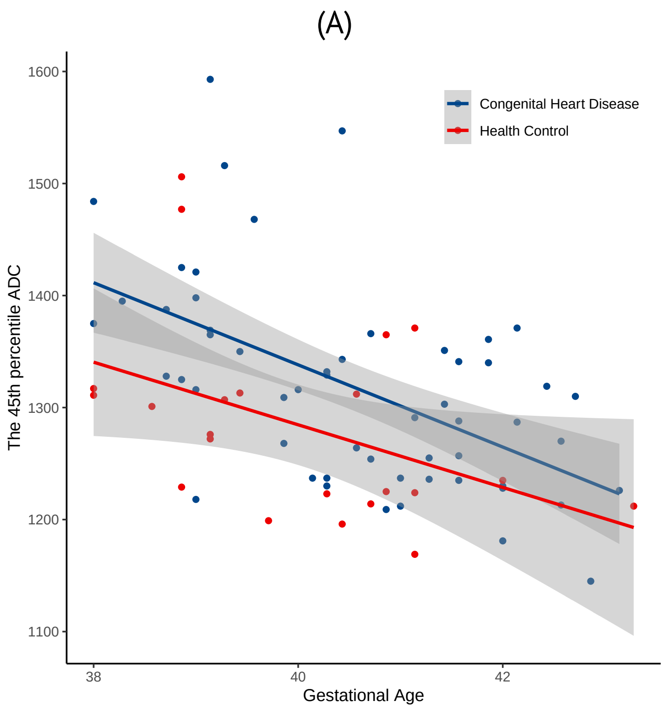

Figure S9. Regression analysis for the 45th percentile apparent diffusion coefficients with gestational age, comparing single- (A) and multi-slice (B) diffusion weighted imaging.

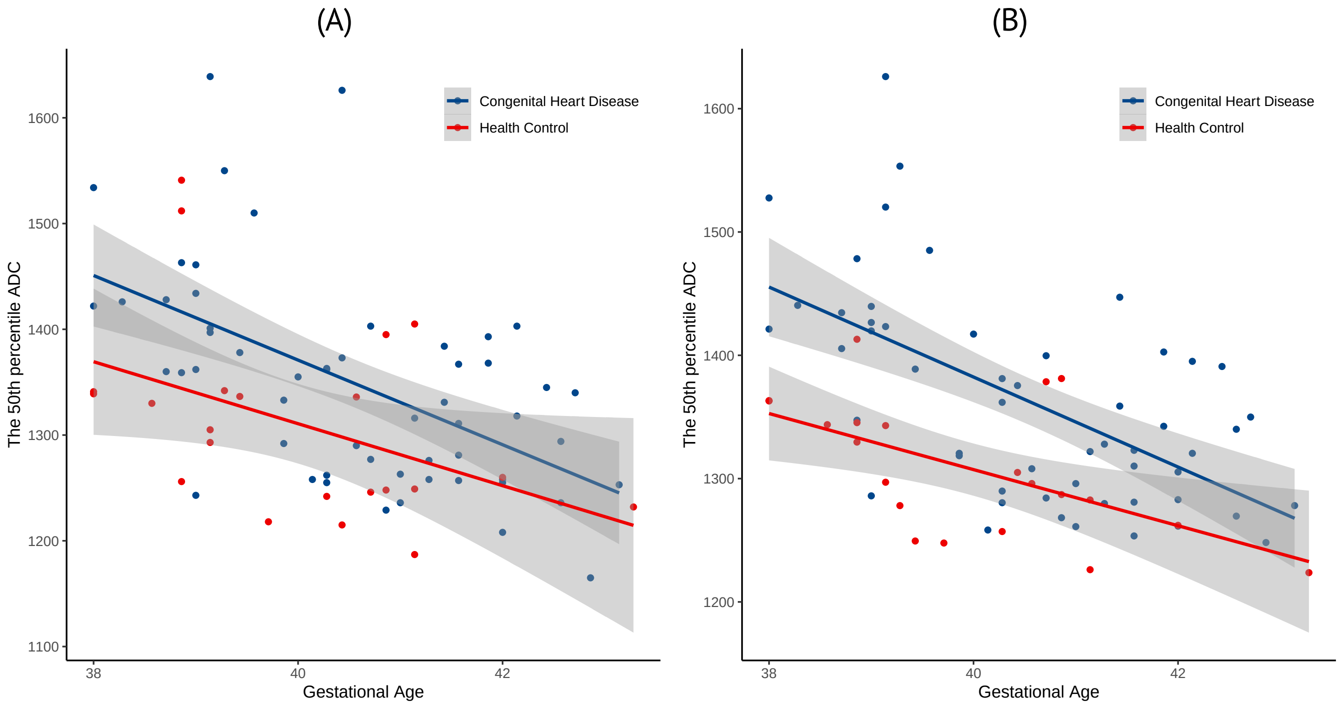

Figure S10. Regression analysis for the 50th percentile apparent diffusion coefficients with gestational age, comparing single- (A) and multi-slice (B) diffusion weighted imaging.

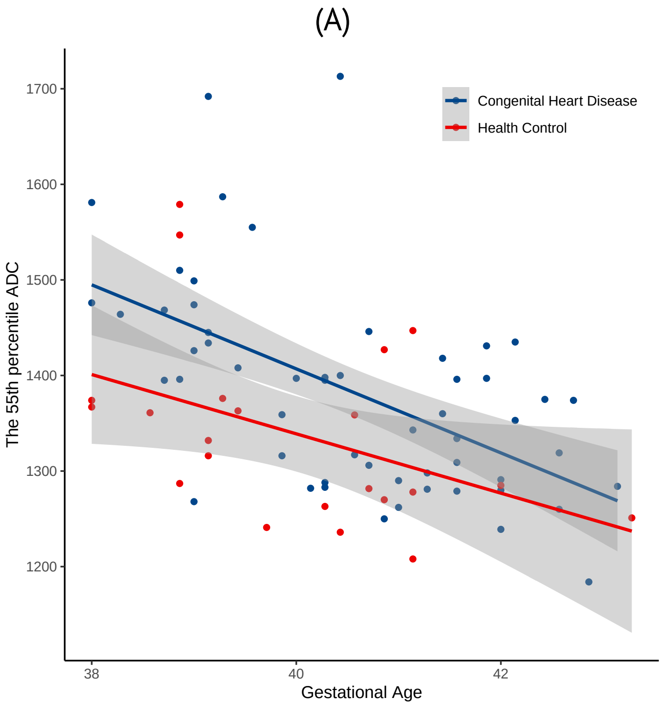

Figure S11. Regression analysis for the 55th percentile apparent diffusion coefficients with gestational age, comparing single- (A) and multi-slice (B) diffusion weighted imaging.

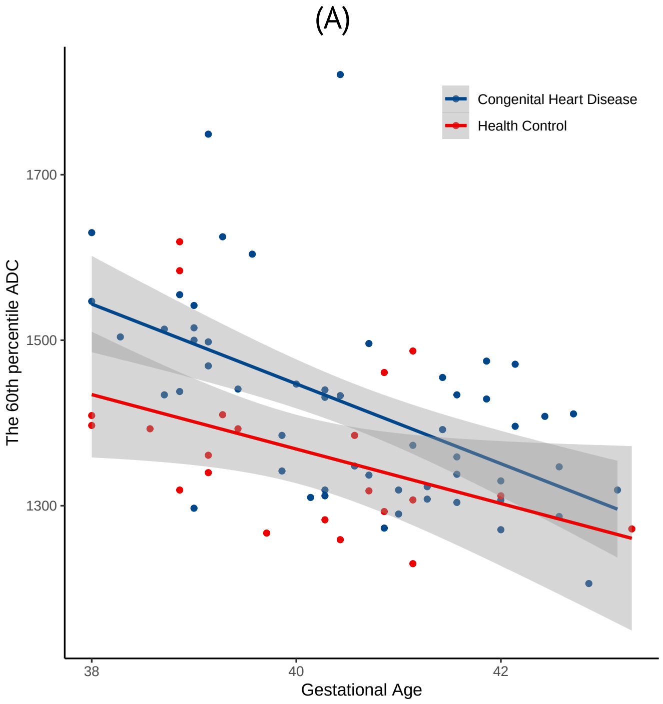

Figure S12. Regression analysis for the 60th percentile apparent diffusion coefficients with gestational age, comparing single- (A) and multi-slice (B) diffusion weighted imaging.

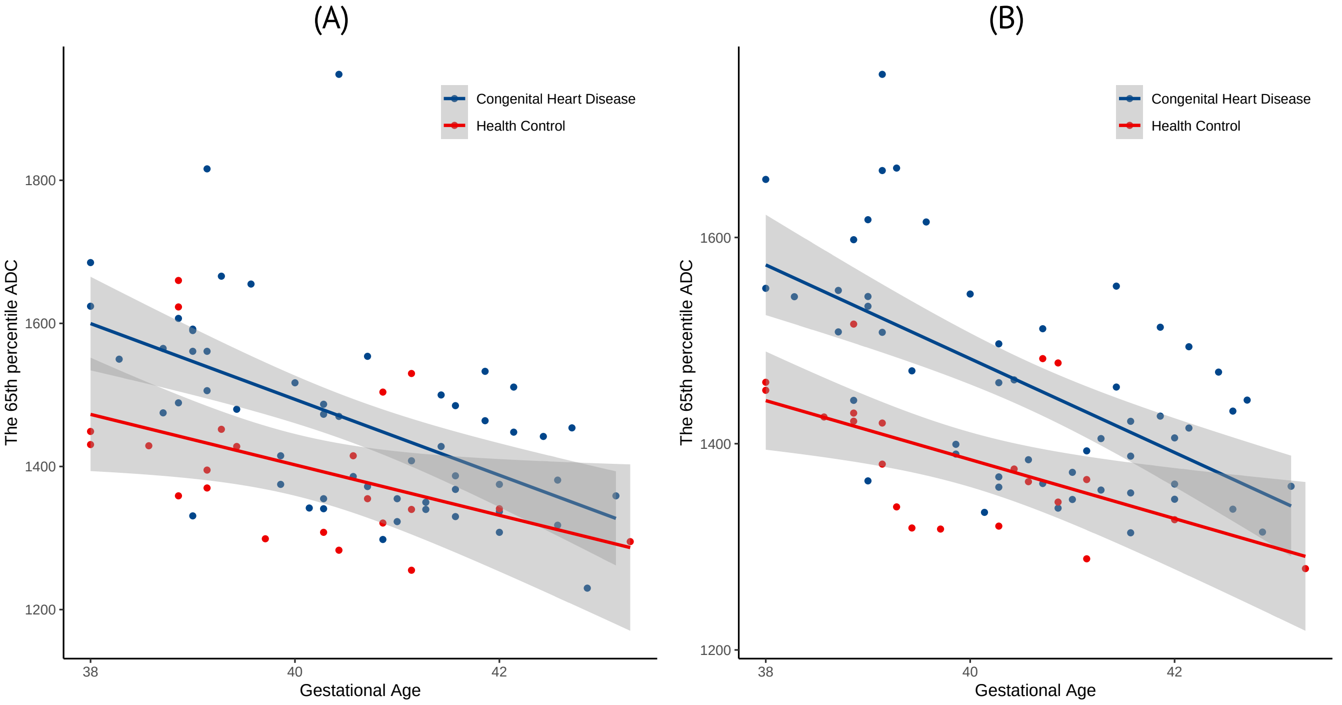

Figure S13. Regression analysis for the 65th percentile apparent diffusion coefficients with gestational age, comparing single- (A) and multi-slice (B) diffusion weighted imaging.

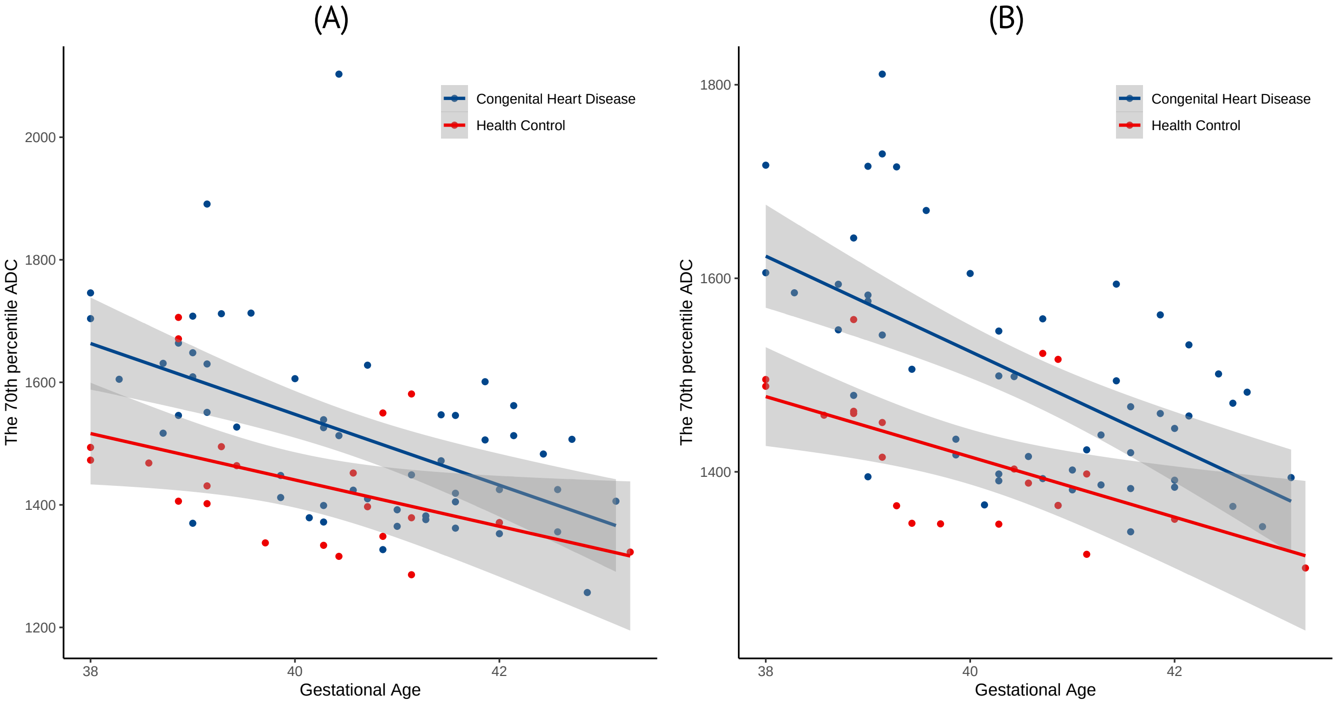

Figure S14. Regression analysis for the 70th percentile apparent diffusion coefficients with gestational age, comparing single- (A) and multi-slice (B) diffusion weighted imaging.

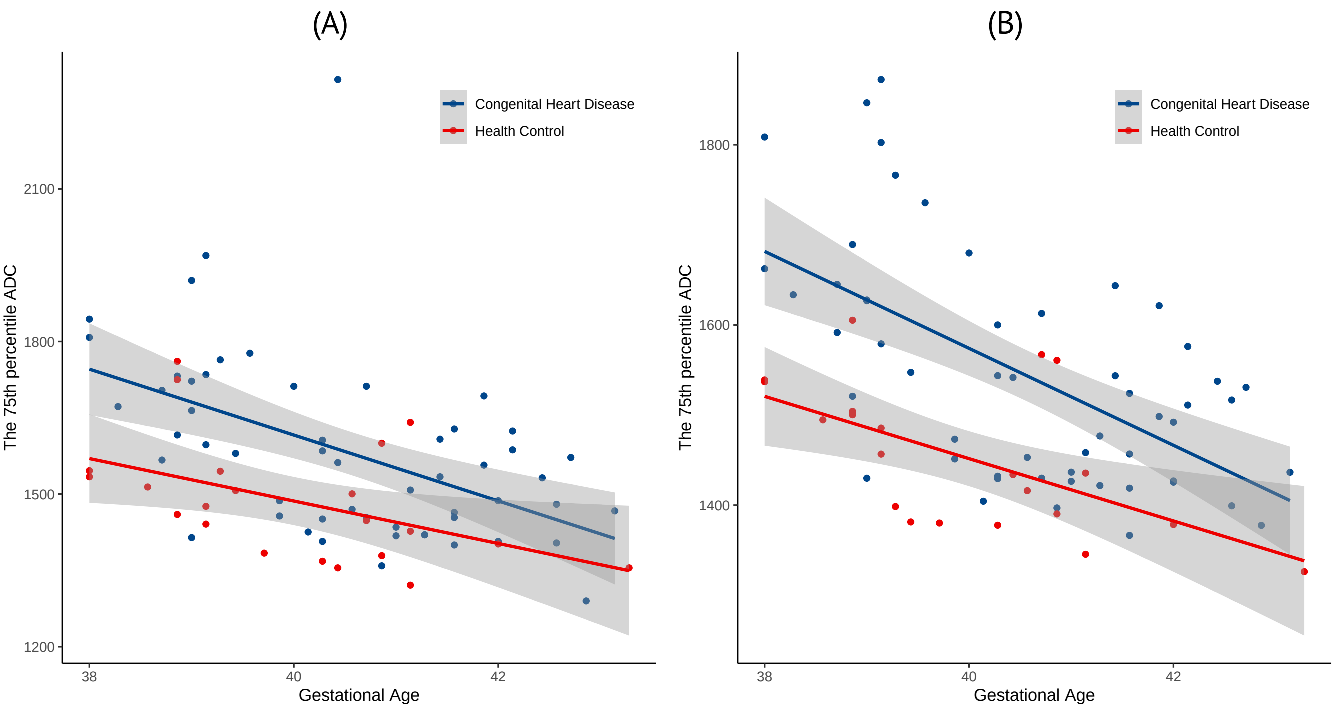

Figure S15. Regression analysis for the 75th percentile apparent diffusion coefficients with gestational age, comparing single- (A) and multi-slice (B) diffusion weighted imaging.

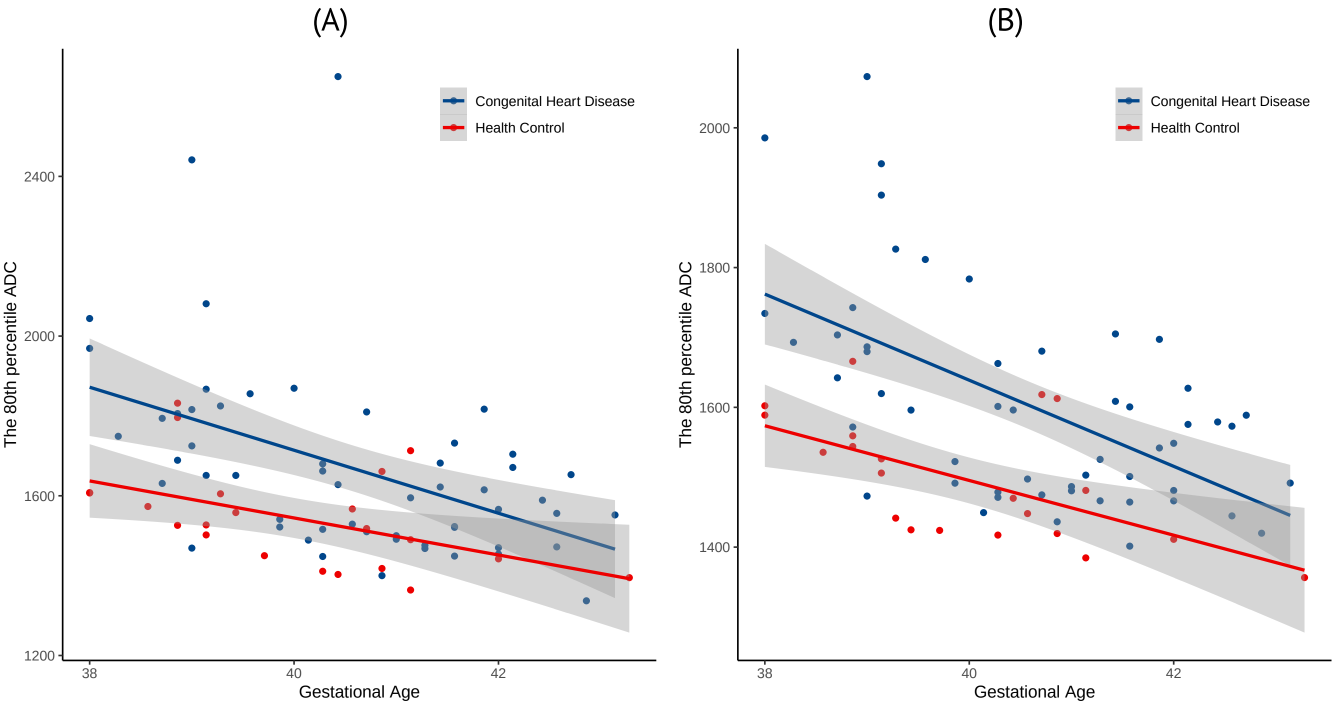

Figure S16. Regression analysis for the 80th percentile apparent diffusion coefficients with gestational age, comparing single- (A) and multi-slice (B) diffusion weighted imaging.

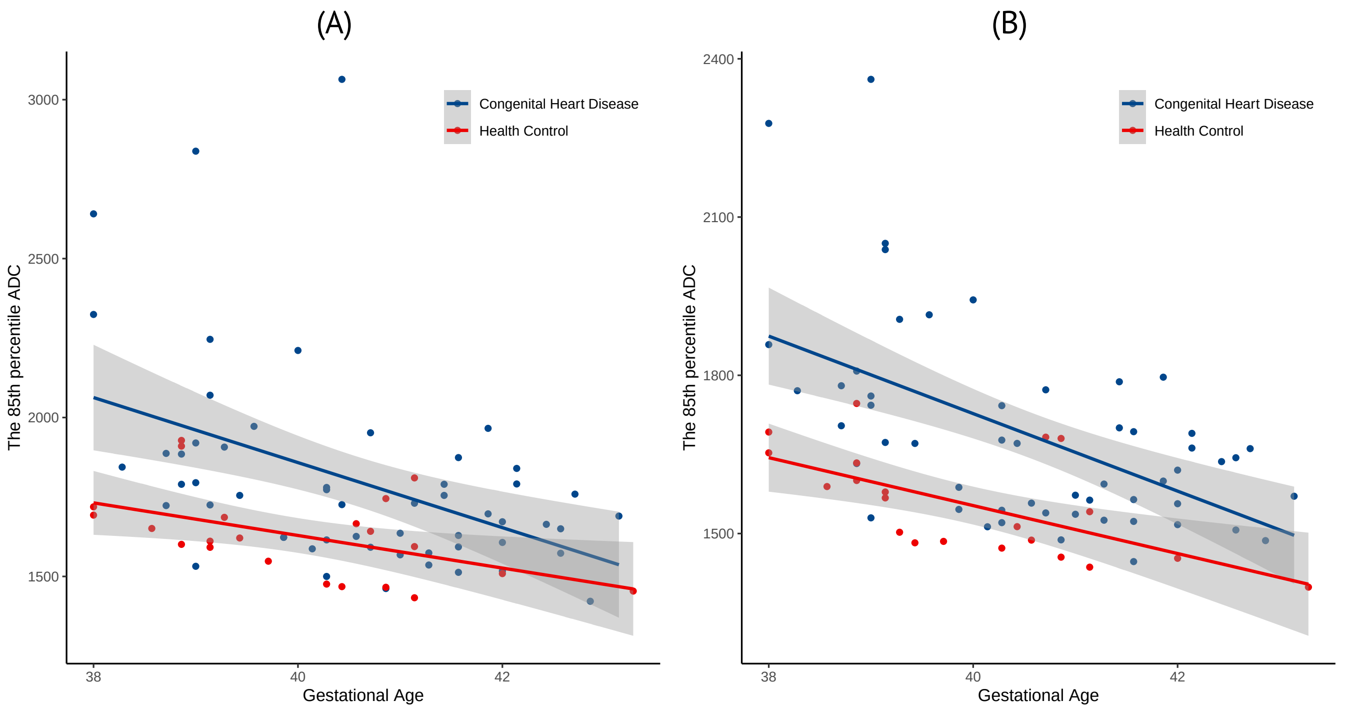

Figure S17. Regression analysis for the 85th percentile apparent diffusion coefficients with gestational age, comparing single- (A) and multi-slice (B) diffusion weighted imaging.

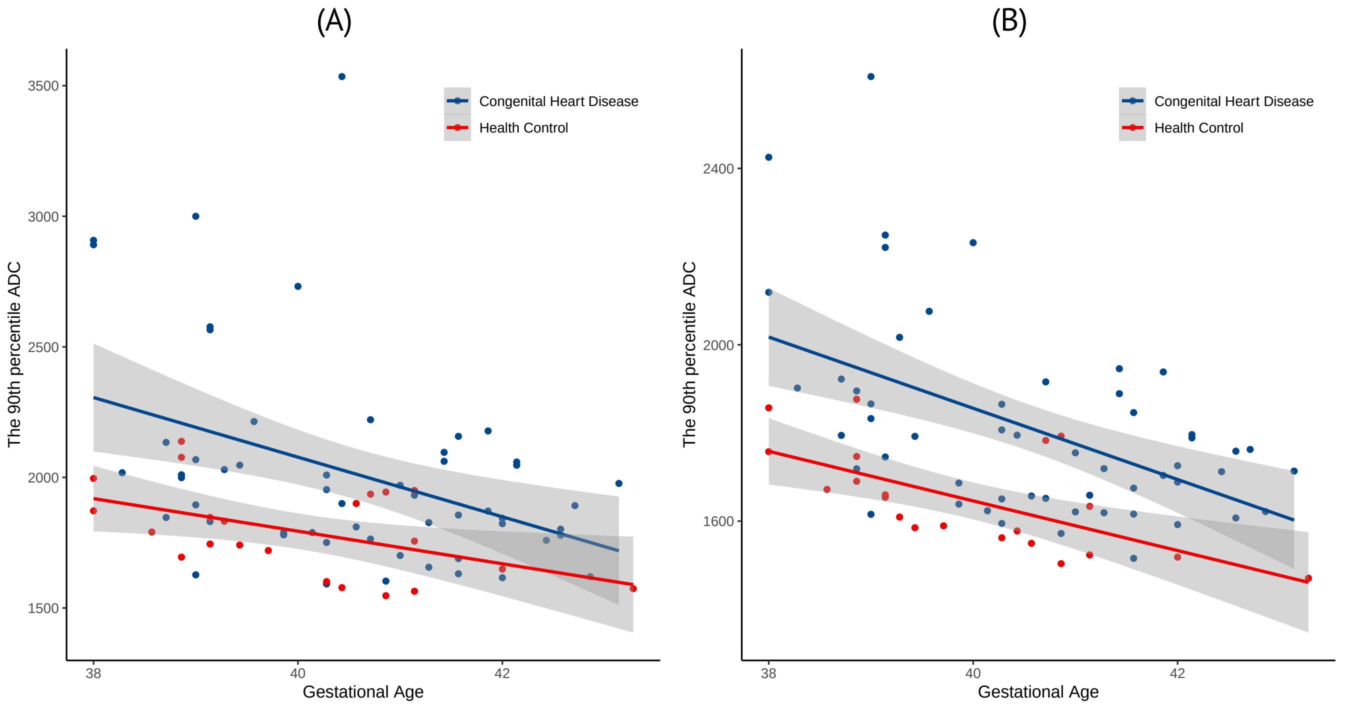

Figure S18. Regression analysis for the 90th percentile apparent diffusion coefficients with gestational age, comparing single- (A) and multi-slice (B) diffusion weighted imaging.

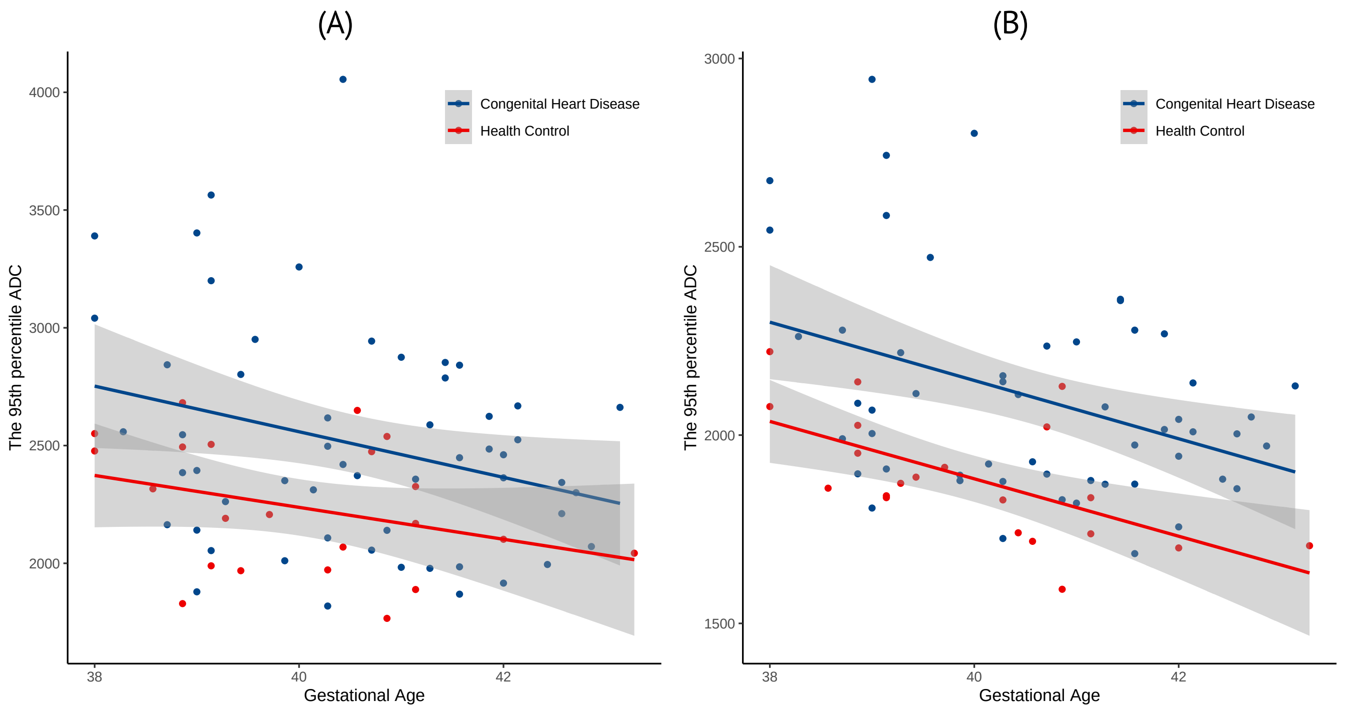

Figure S19. Regression analysis for the 95th percentile apparent diffusion coefficients with gestational age, comparing single- (A) and multi-slice (B) diffusion weighted imaging.

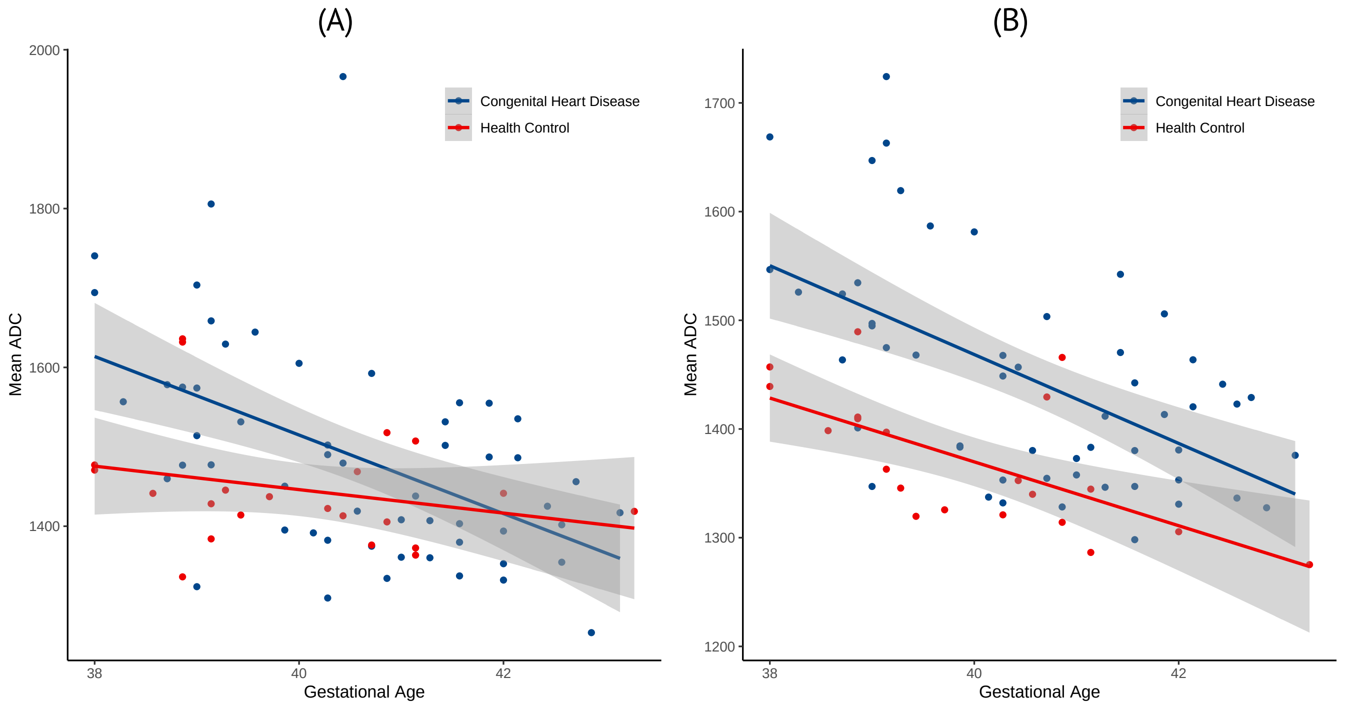

Figure S20. Regression analysis for the mean of apparent diffusion coefficients with gestational age, comparing single- (A) and multi-slice (B) diffusion weighted imaging.

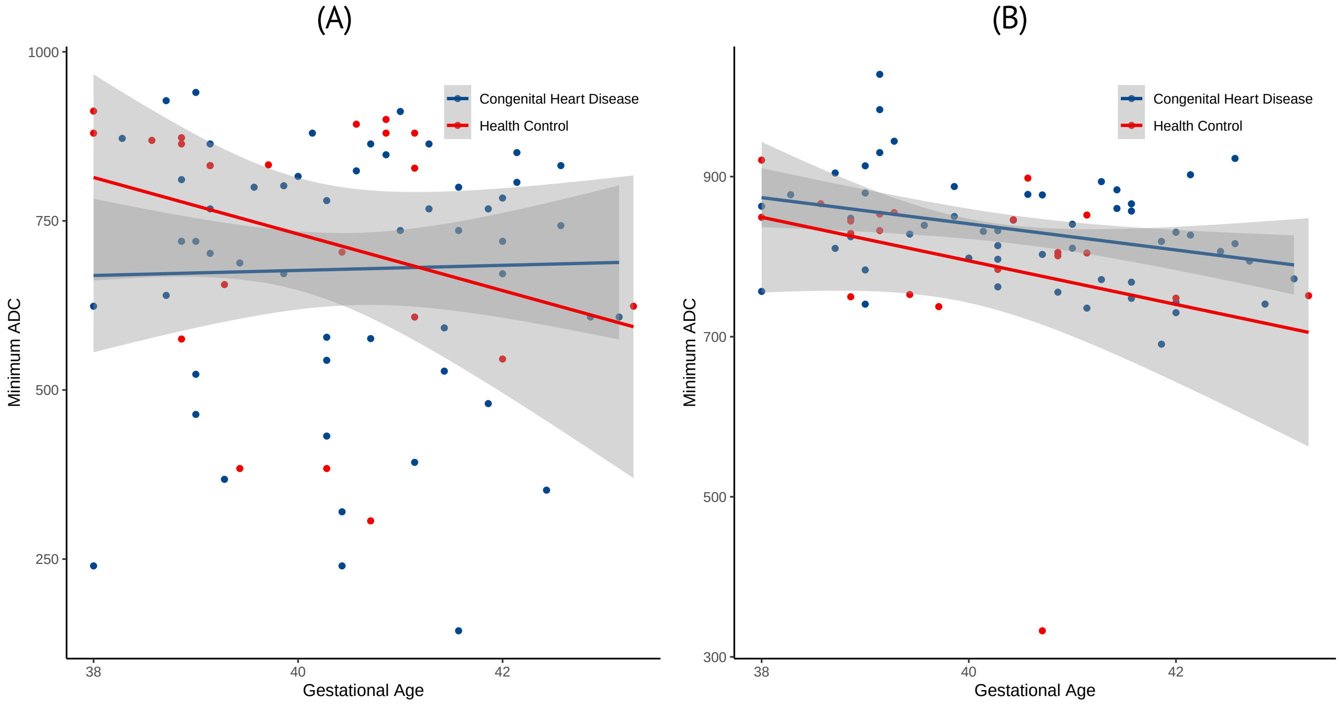

Figure S21. Regression analysis for the minimum of apparent diffusion coefficients with gestational age, comparing single- (A) and multi-slice (B) diffusion weighted imaging.

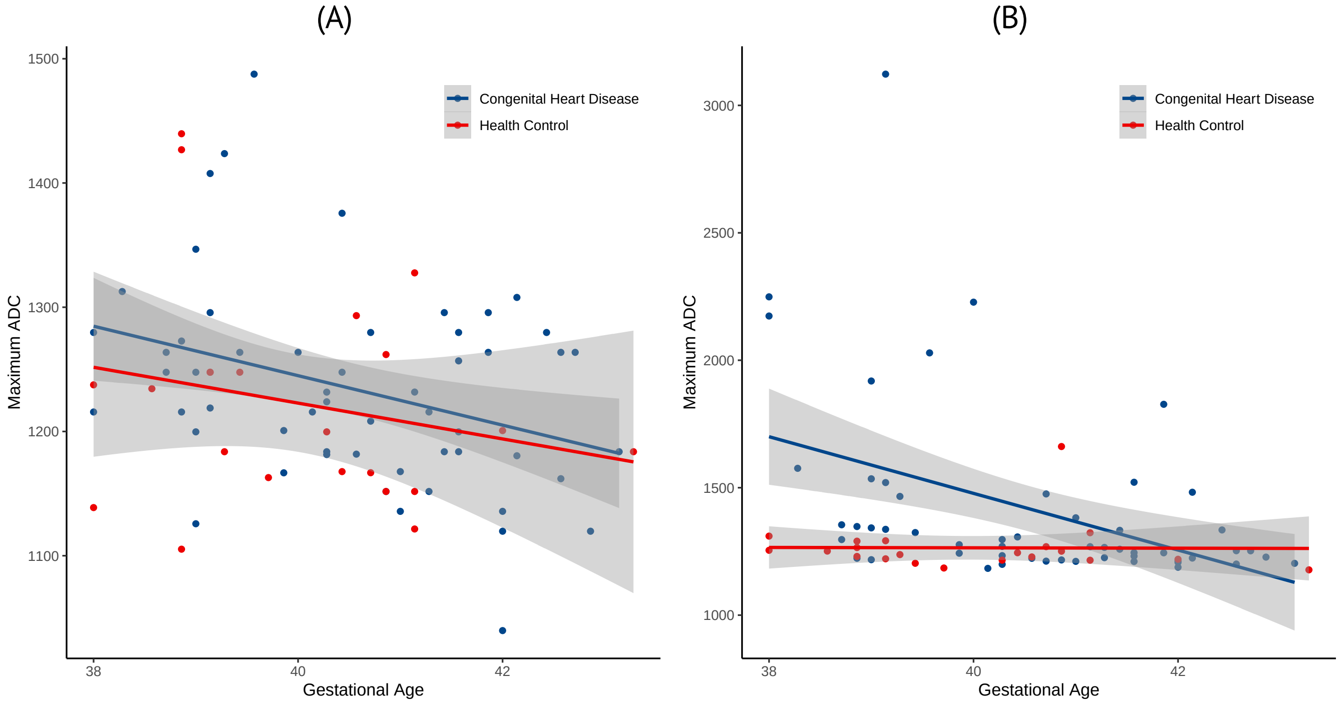

Figure S22. Regression analysis for the maximum of apparent diffusion coefficients with gestational age, comparing single- (A) and multi-slice (B) diffusion weighted imaging.

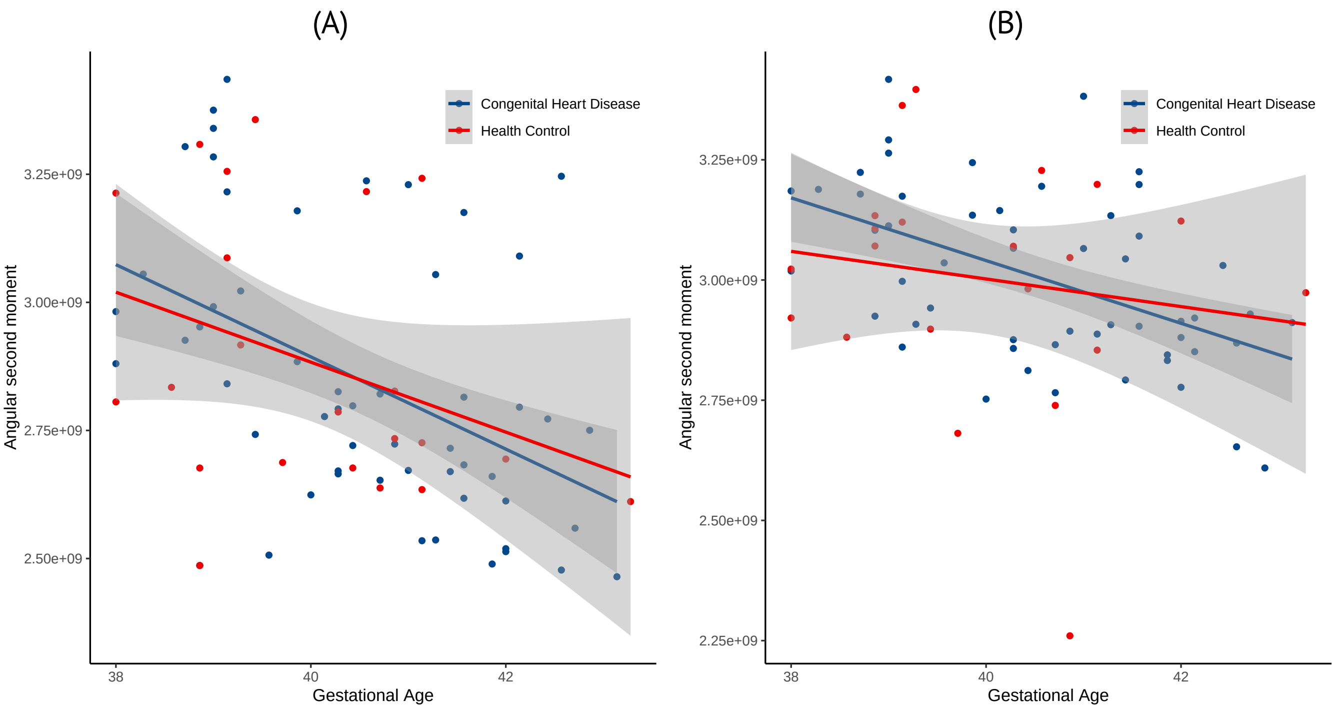

Figure S23. Regression analysis for the angular second moment with gestational age, comparing single- (A) and multi-slice (B) diffusion weighted imaging.

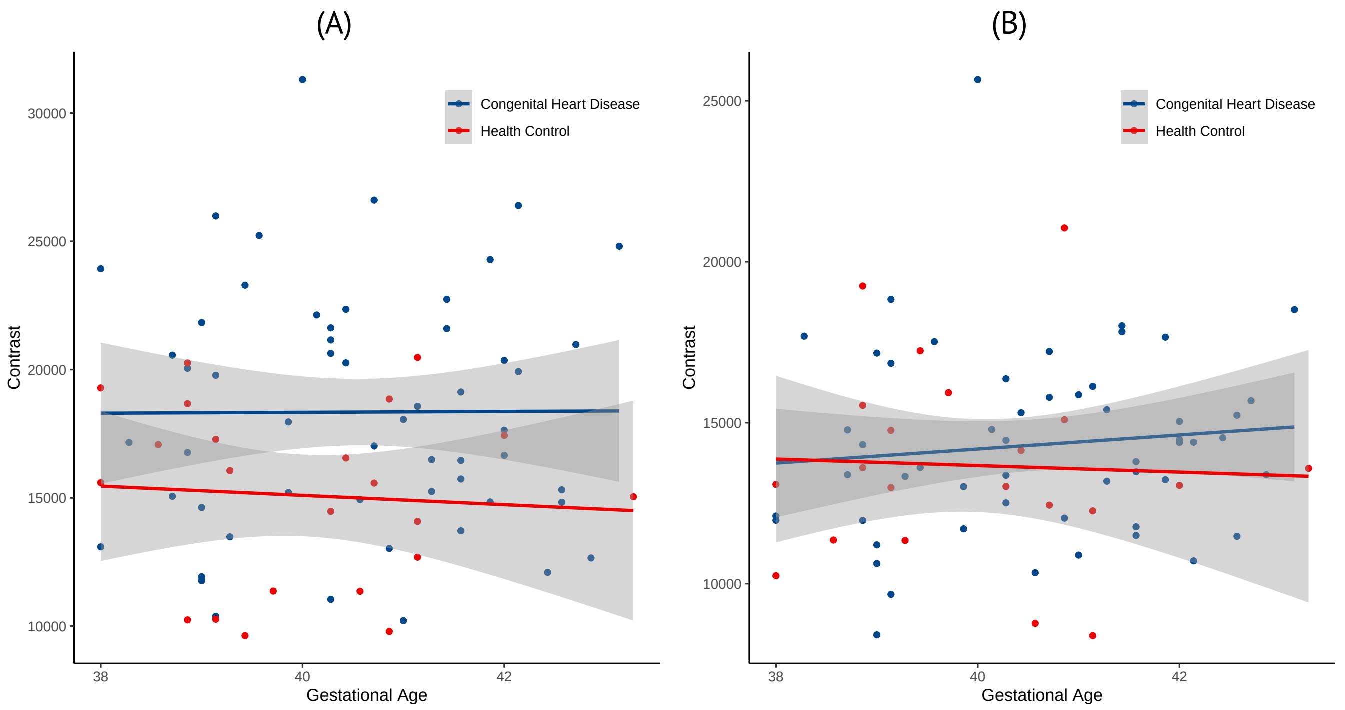

Figure S24. Regression analysis for the contrast with gestational age, comparing single- (A) and multi-slice (B) diffusion weighted imaging.

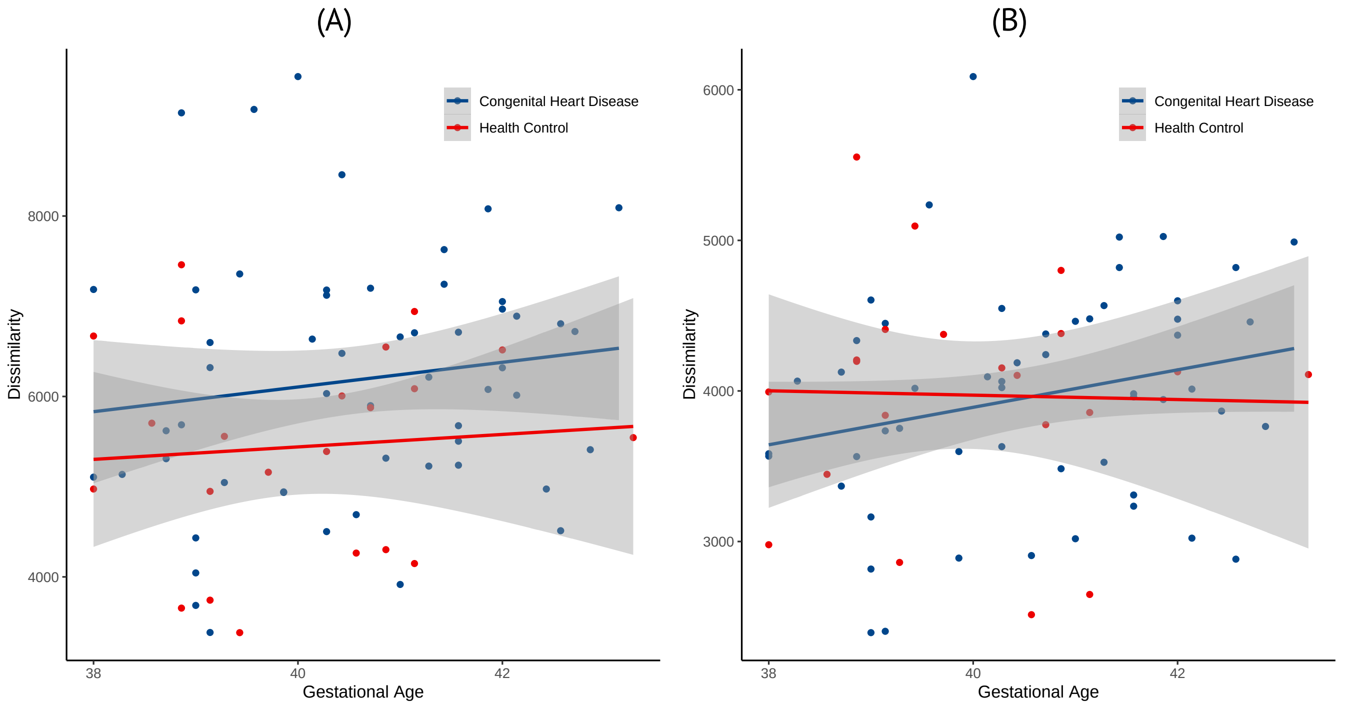

Figure S25. Regression analysis for the dissimilarity with gestational age, comparing single- (A) and multi-slice (B) diffusion weighted imaging.

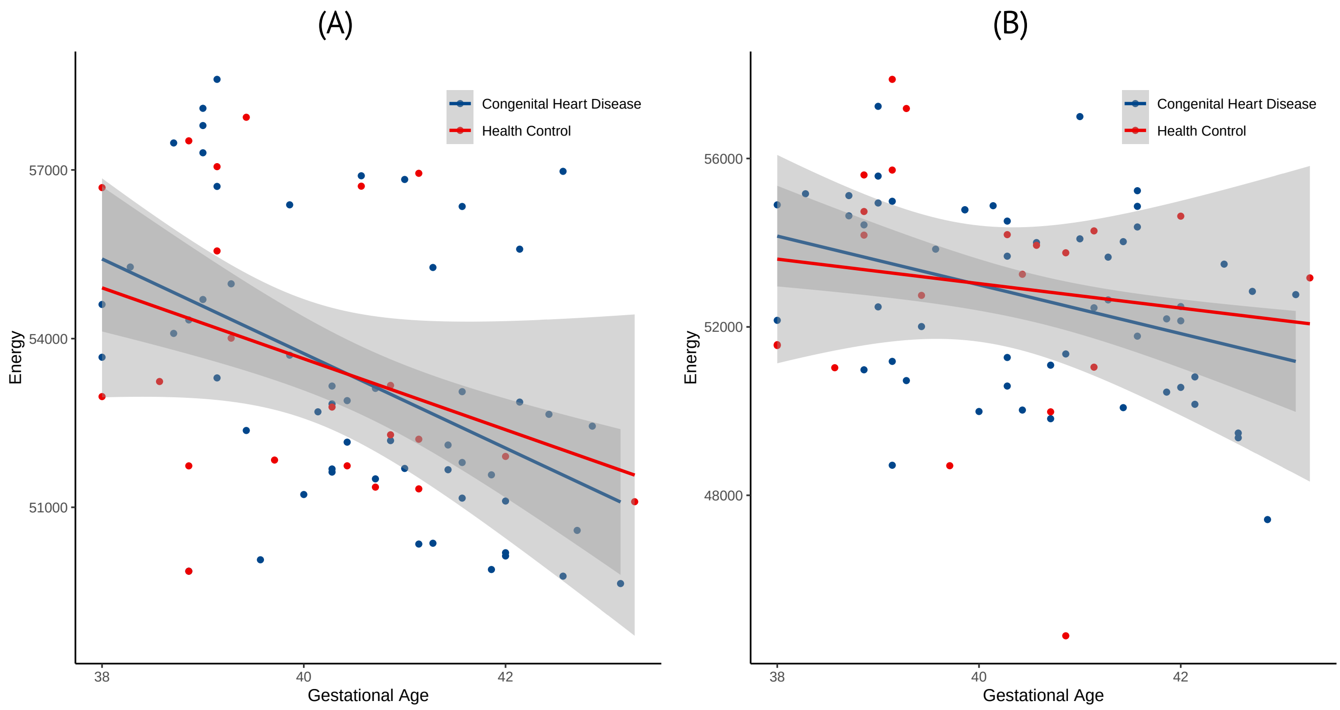

Figure S26. Regression analysis for the energy with gestational age, comparing single- (A) and multi-slice (B) diffusion weighted imaging.

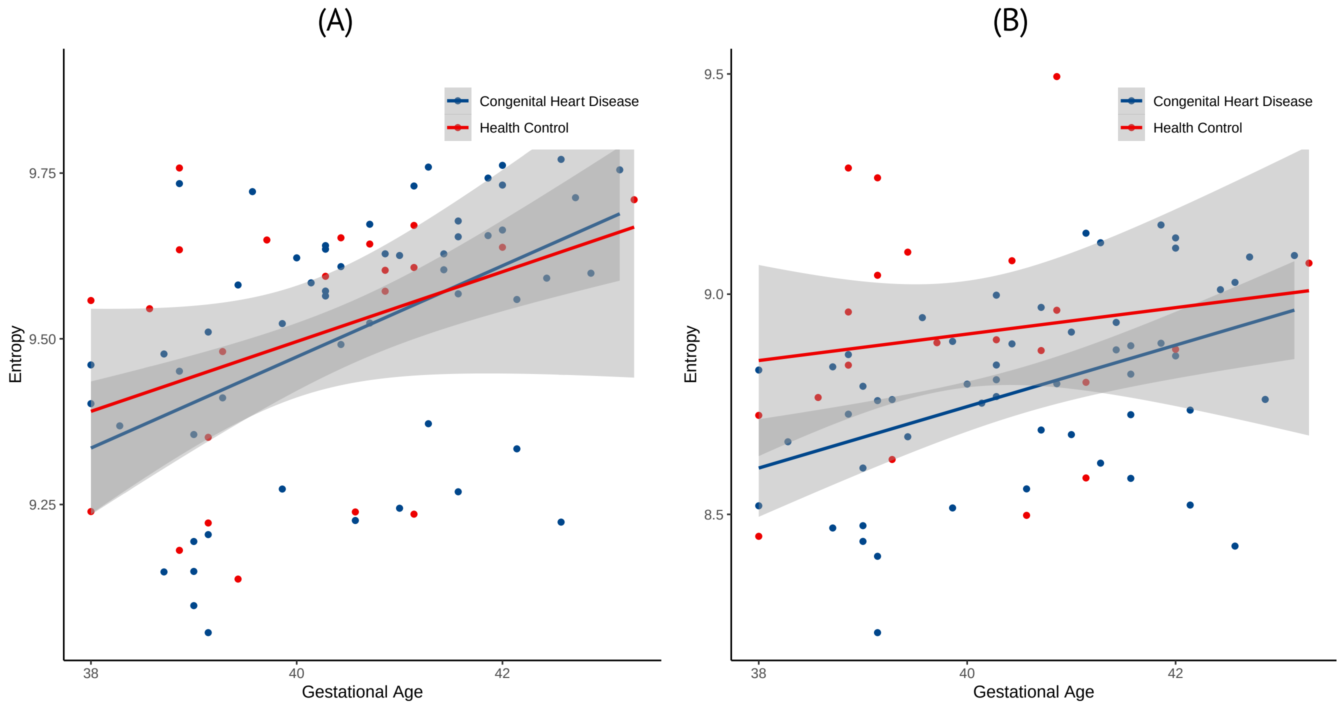

Figure S27. Regression analysis for the entropy with gestational age, comparing single- (A) and multi-slice (B) diffusion weighted imaging.

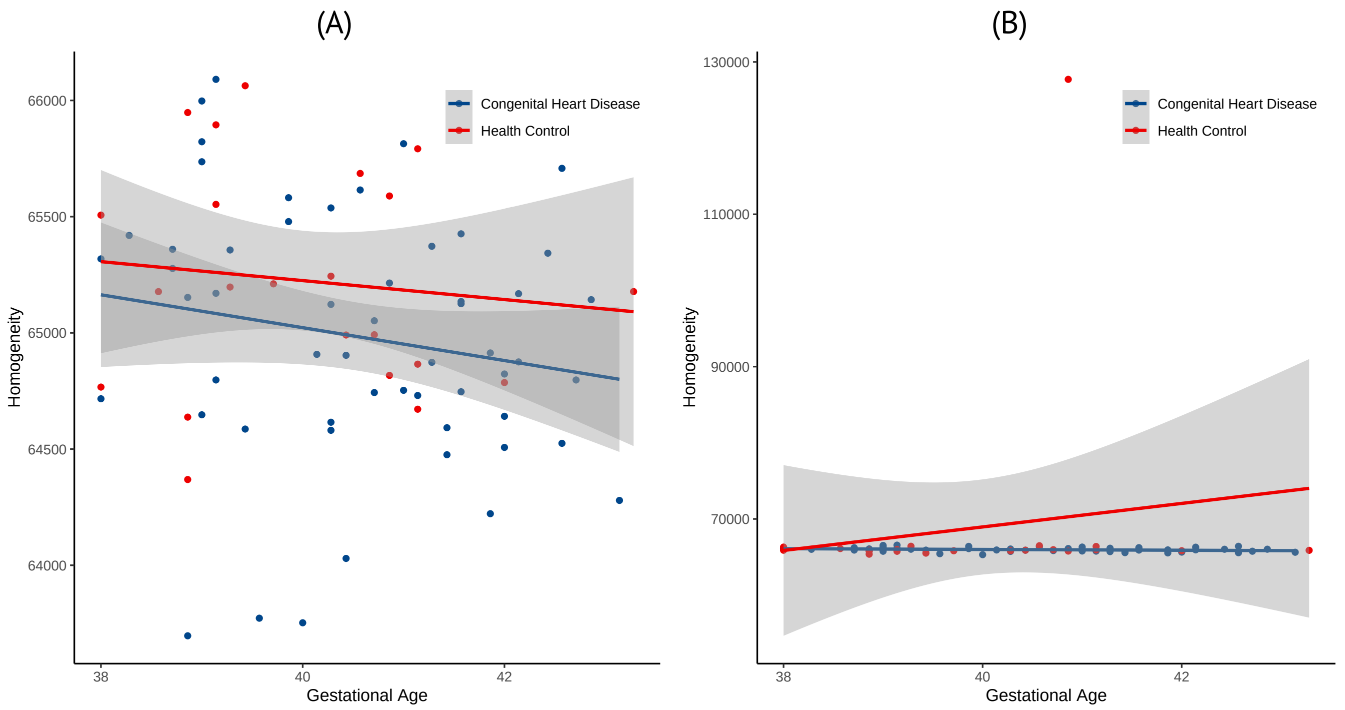

Figure S28. Regression analysis for the homogeneity with gestational age, comparing single- (A) and multi-slice (B) diffusion weighted imaging.

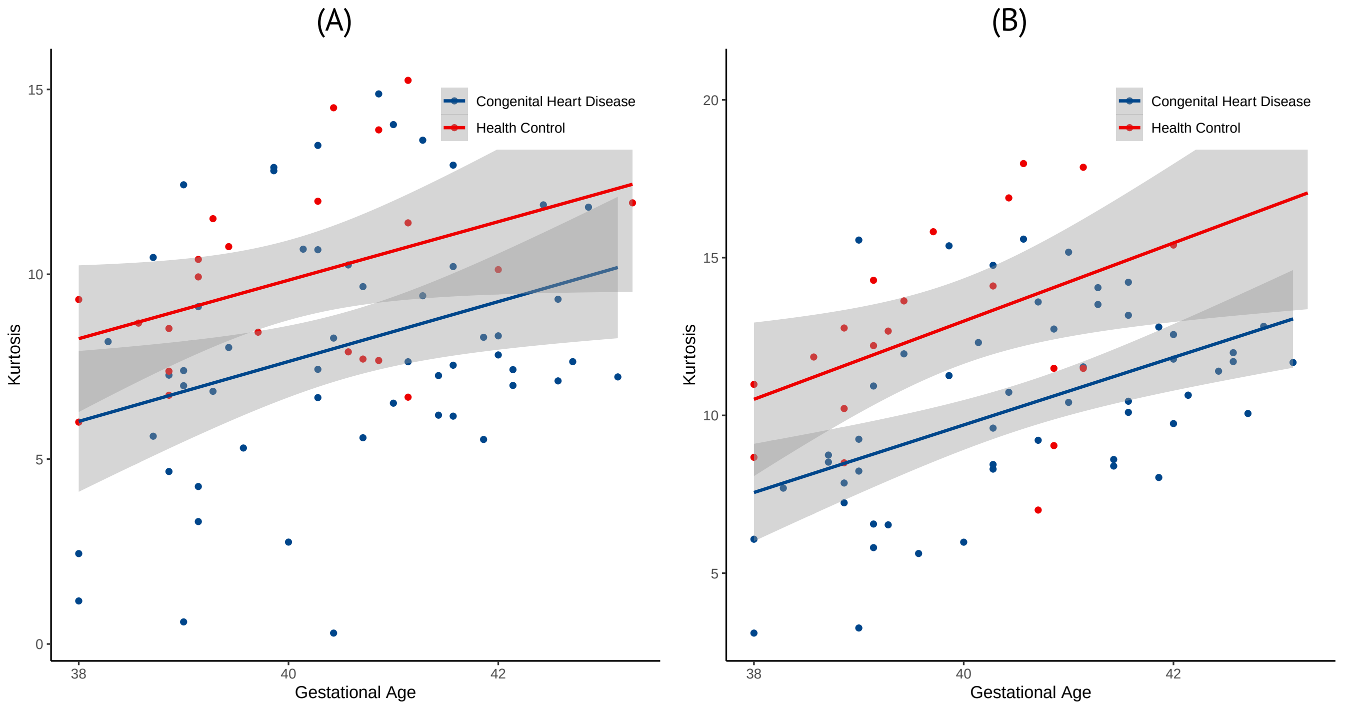

Figure S29. Regression analysis for the kurtosis with gestational age, comparing single- (A) and multi-slice (B) diffusion weighted imaging.

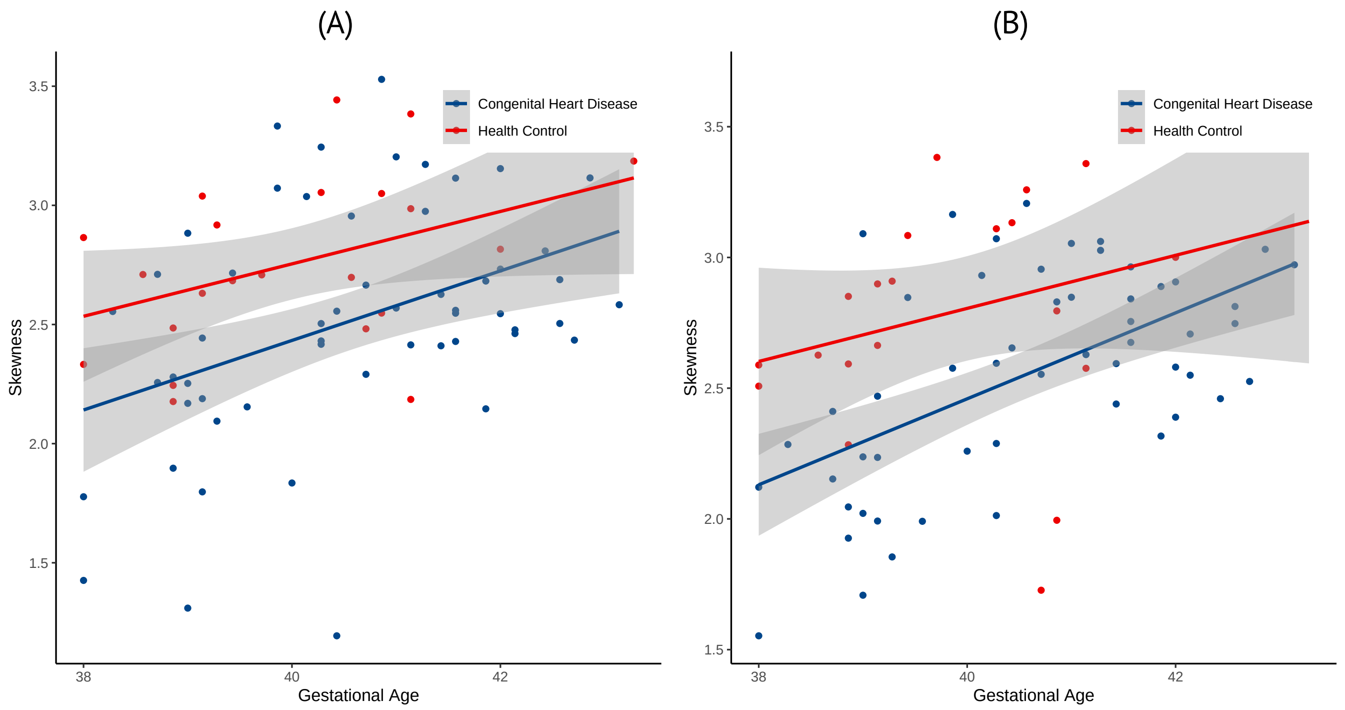

Figure S30. Regression analysis for the skewness with gestational age, comparing single- (A) and multi-slice (B) diffusion weighted imaging.

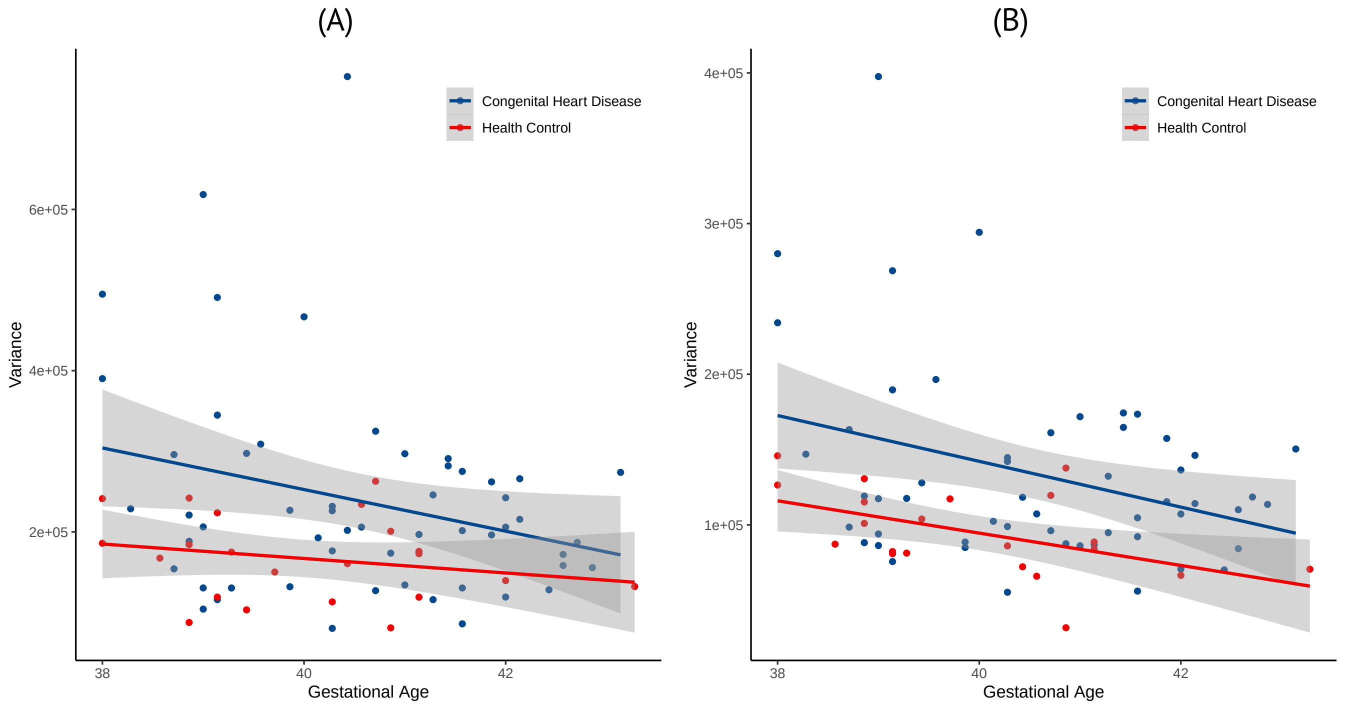

Figure S31. Regression analysis for the variance with gestational age, comparing single- (A) and multi-slice (B) diffusion weighted imaging.

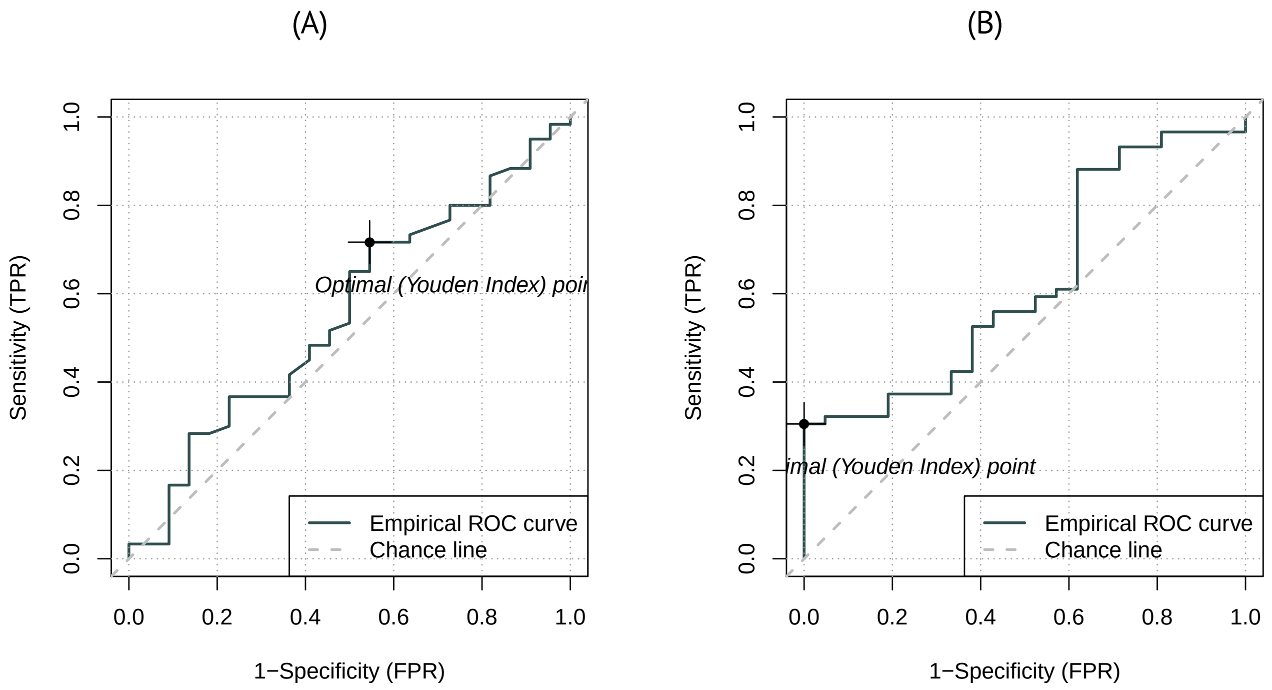

Figure S32. Receiver operating characteristics analysis with optimal point for the 5th percentile apparent diffusion coefficients in diagnosing congenital heart disease, comparing single- (A) and multi-slice (B) diffusion weighted imaging.

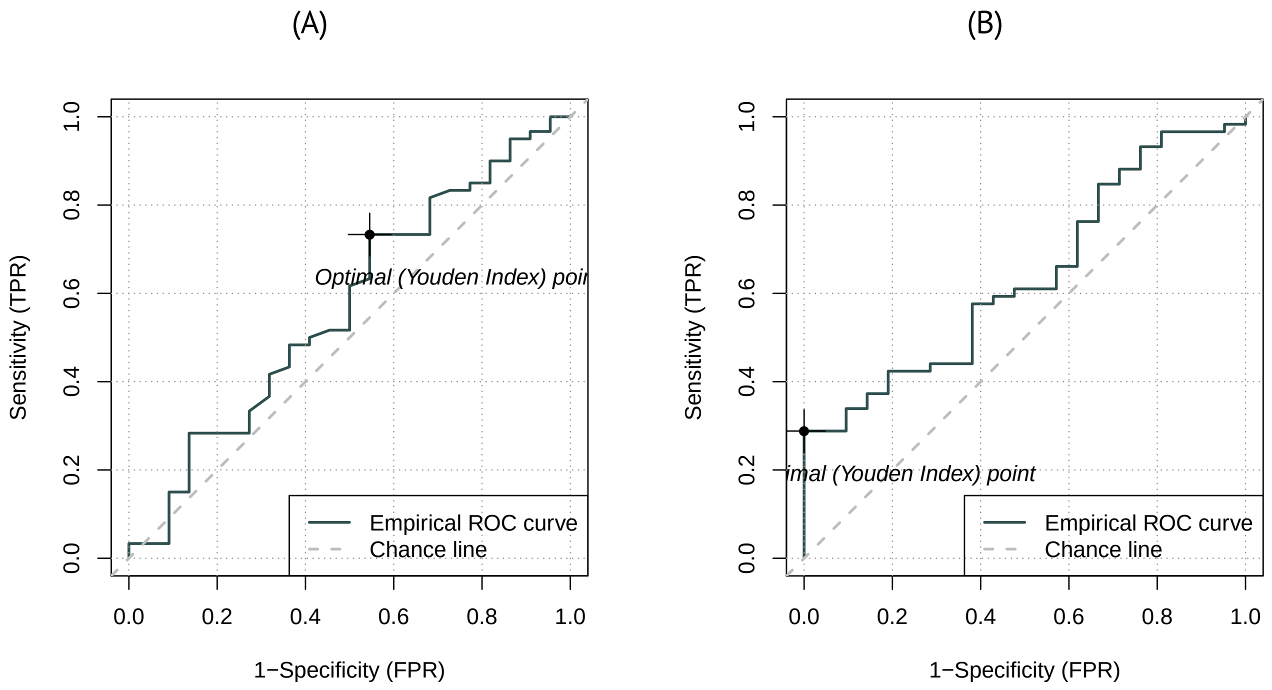

Figure S33. Receiver operating characteristics analysis with optimal point for the 10th percentile apparent diffusion coefficients in diagnosing congenital heart disease, comparing single- (A) and multi-slice (B) diffusion weighted imaging.

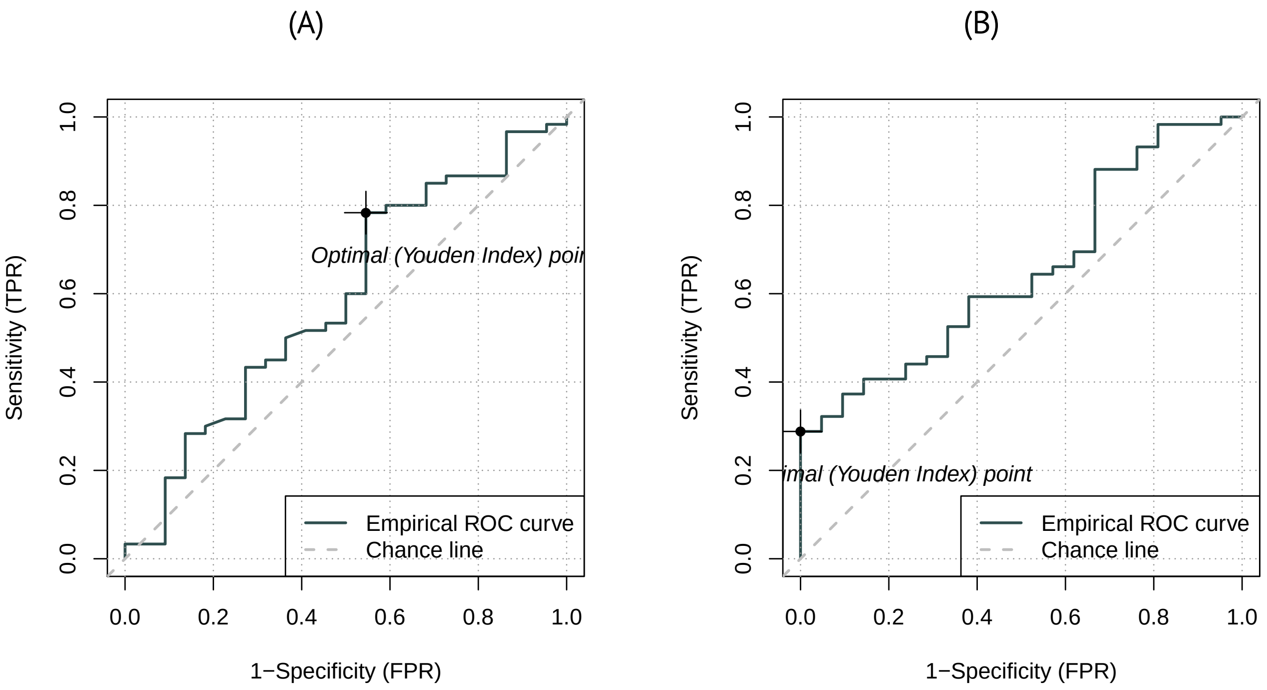

Figure S34. Receiver operating characteristics analysis with optimal point for the 15th percentile apparent diffusion coefficients in diagnosing congenital heart disease, comparing single- (A) and multi-slice (B) diffusion weighted imaging.

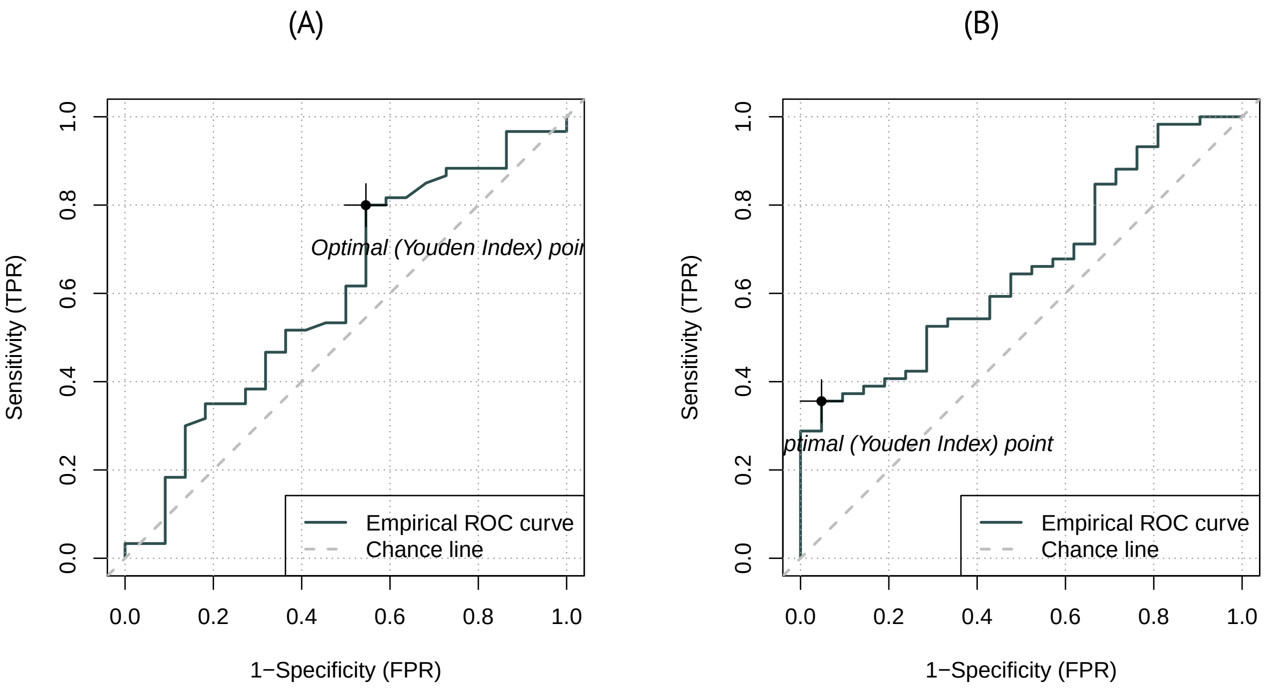

Figure S35. Receiver operating characteristics analysis with optimal point for the 20th percentile apparent diffusion coefficients in diagnosing congenital heart disease, comparing single- (A) and multi-slice (B) diffusion weighted imaging.

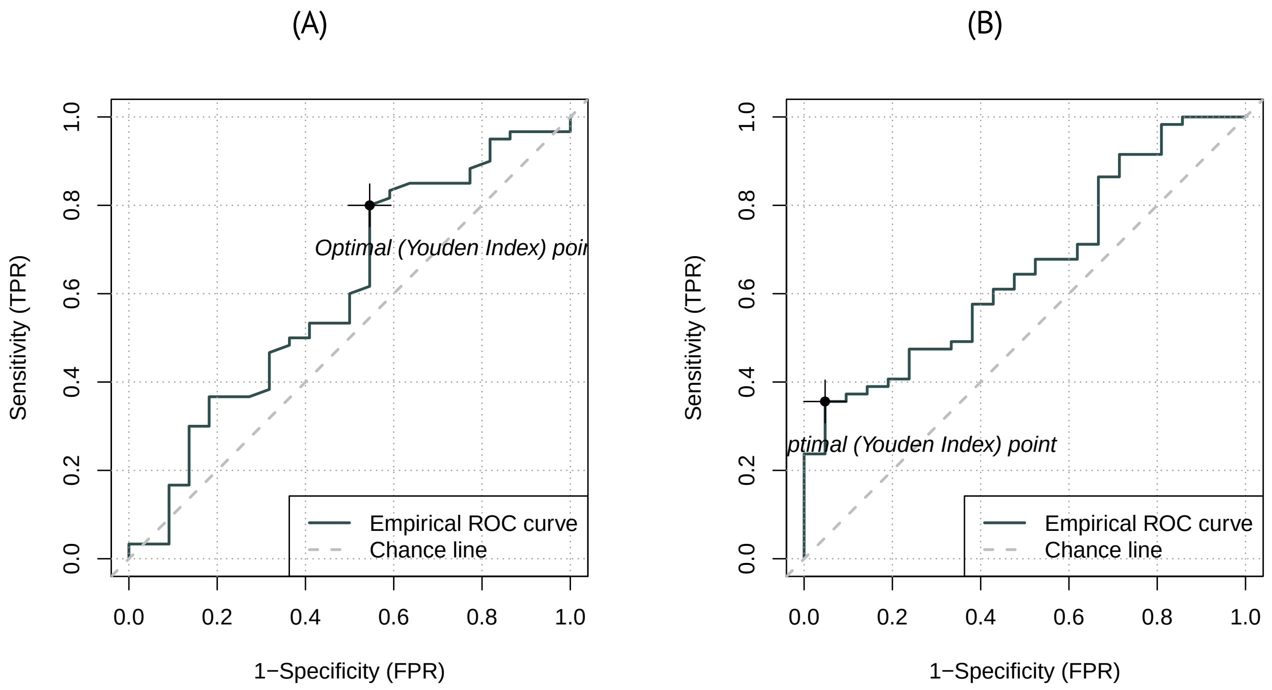

Figure S36. Receiver operating characteristics analysis with optimal point for the 25th percentile apparent diffusion coefficients in diagnosing congenital heart disease, comparing single- (A) and multi-slice (B) diffusion weighted imaging.

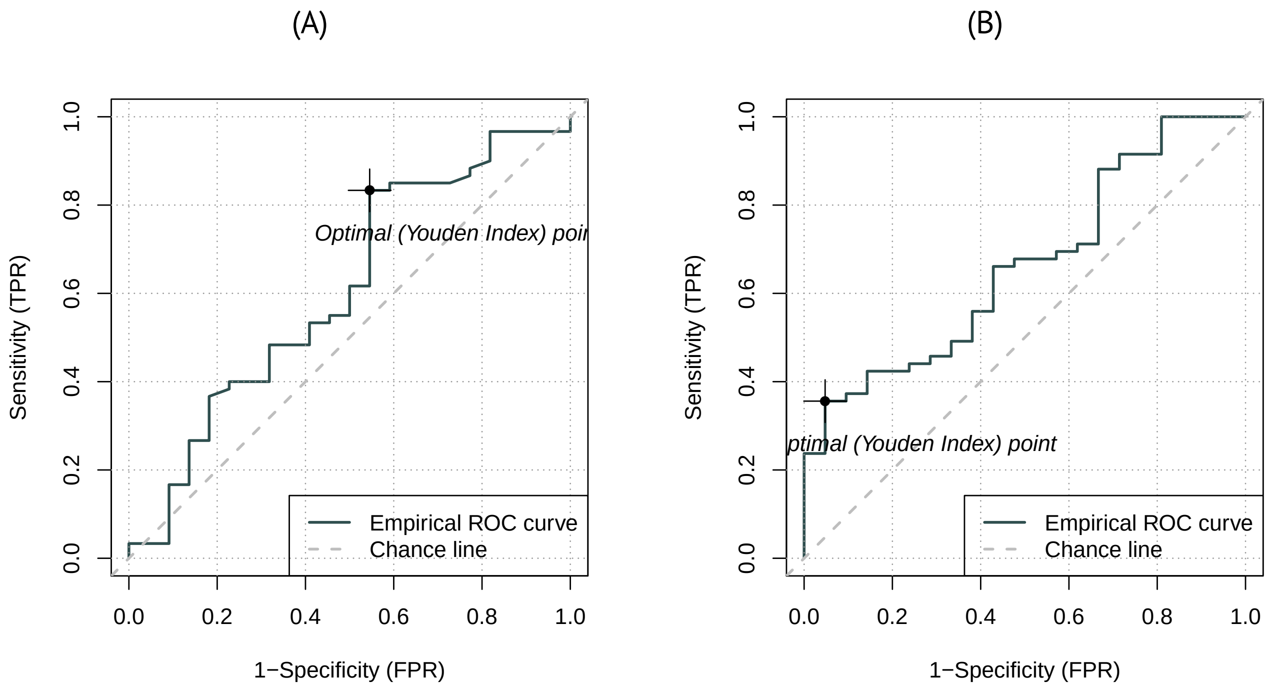

Figure S37. Receiver operating characteristics analysis with optimal point for the 30th percentile apparent diffusion coefficients in diagnosing congenital heart disease, comparing single- (A) and multi-slice (B) diffusion weighted imaging.

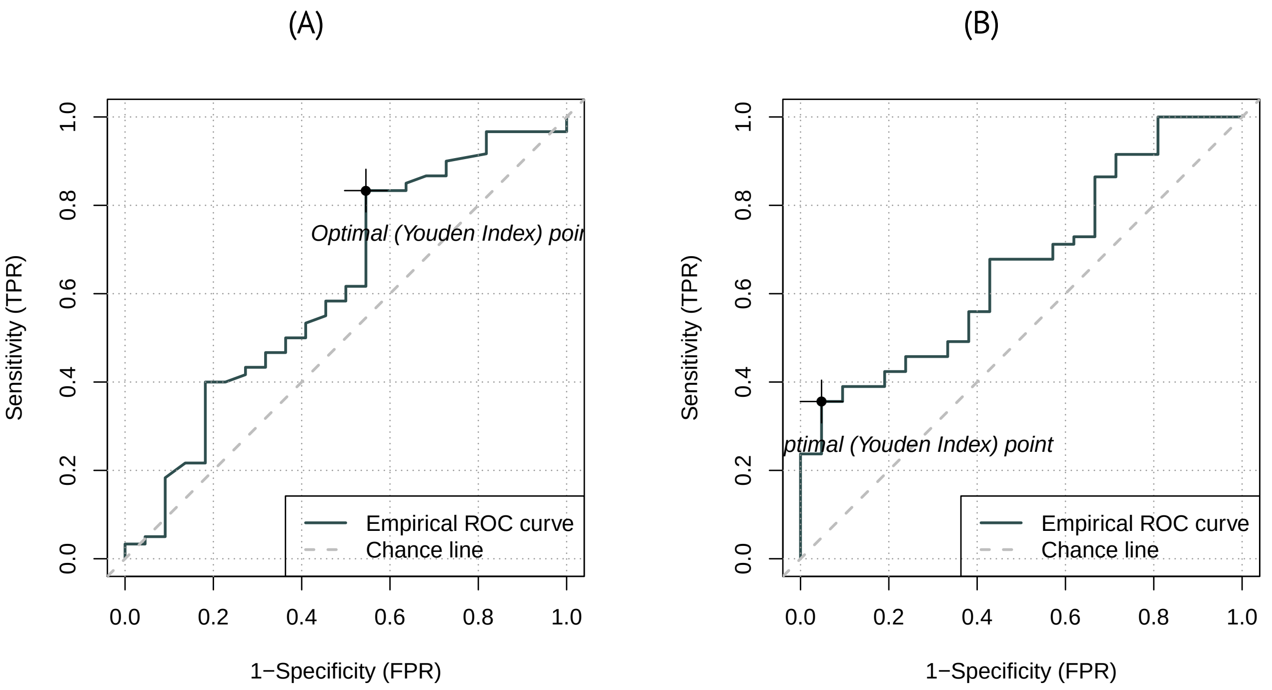

Figure S38. Receiver operating characteristics analysis with optimal point for the 35th percentile apparent diffusion coefficients in diagnosing congenital heart disease, comparing single- (A) and multi-slice (B) diffusion weighted imaging.

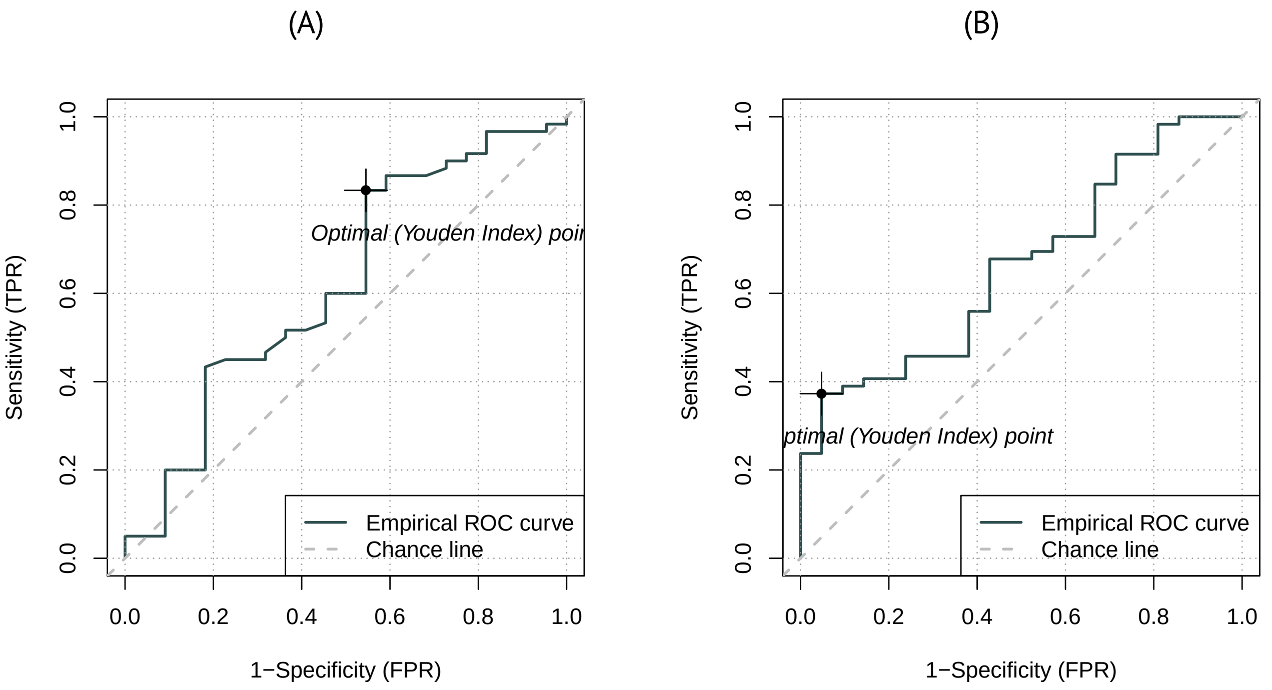

Figure S39. Receiver operating characteristics analysis with optimal point for the 40th percentile apparent diffusion coefficients in diagnosing congenital heart disease, comparing single- (A) and multi-slice (B) diffusion weighted imaging.

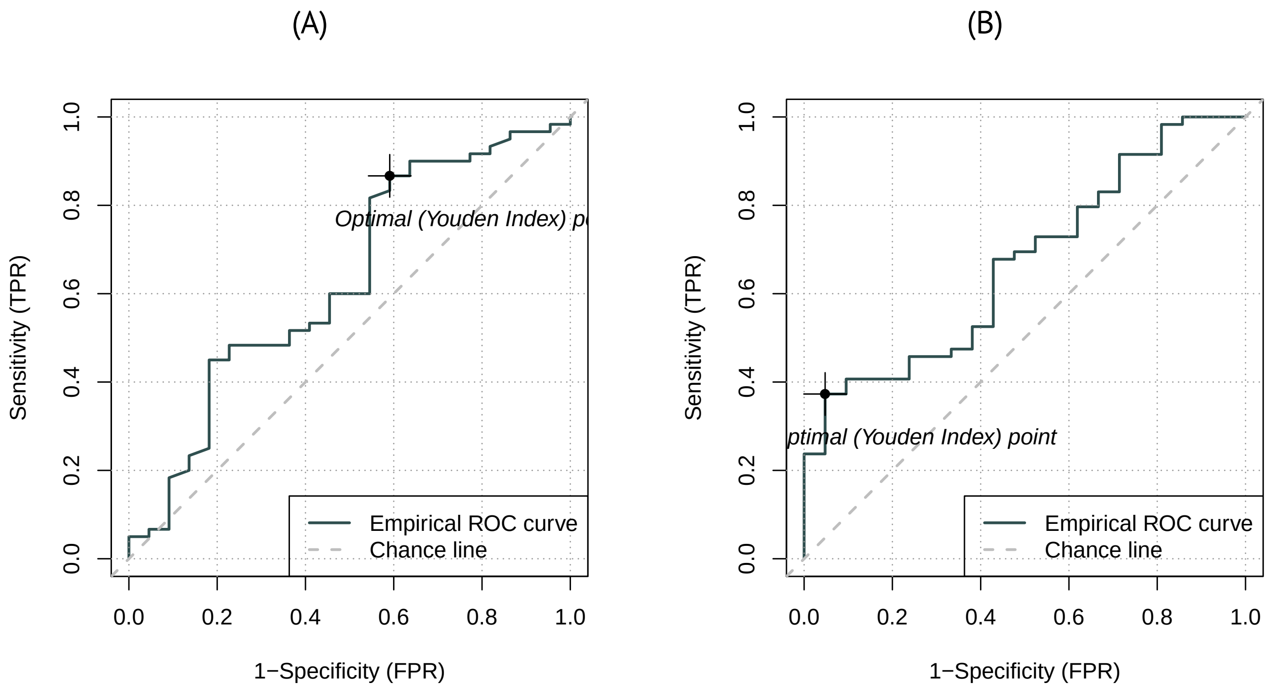

Figure S40. Receiver operating characteristics analysis with optimal point for the 45th percentile apparent diffusion coefficients in diagnosing congenital heart disease, comparing single- (A) and multi-slice (B) diffusion weighted imaging.

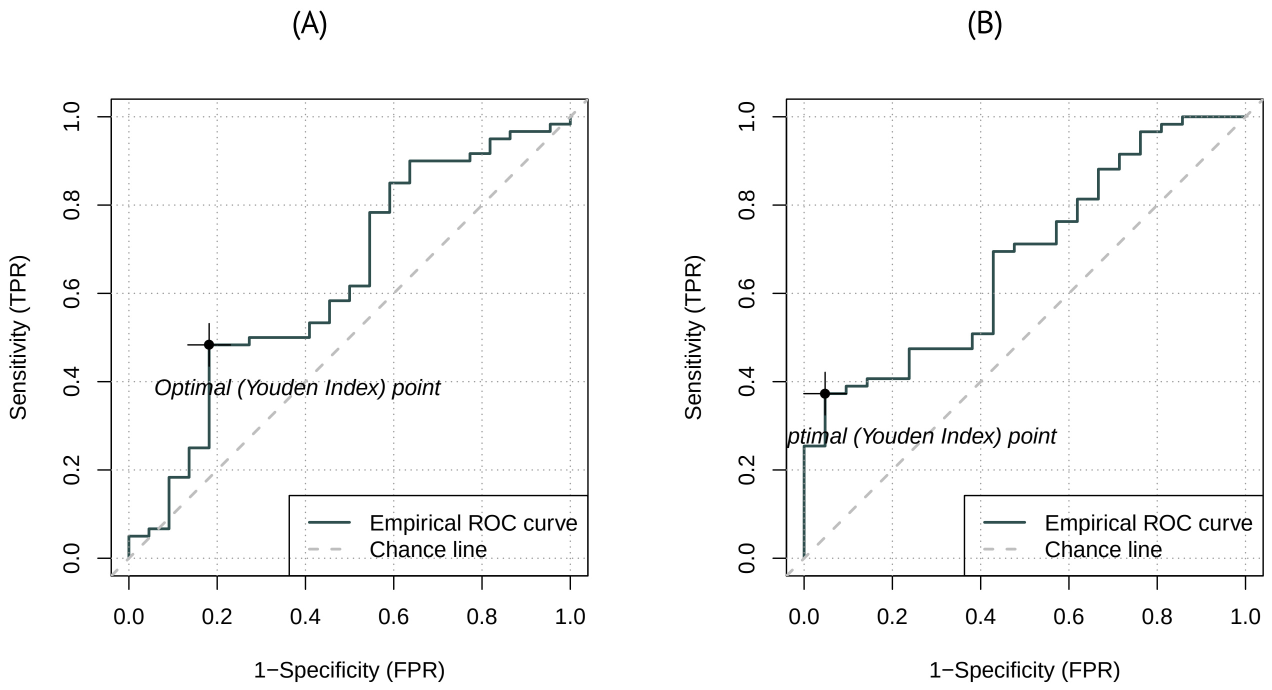

Figure S41. Receiver operating characteristics analysis with optimal point for the 50th percentile apparent diffusion coefficients in diagnosing congenital heart disease, comparing single- (A) and multi-slice (B) diffusion weighted imaging.

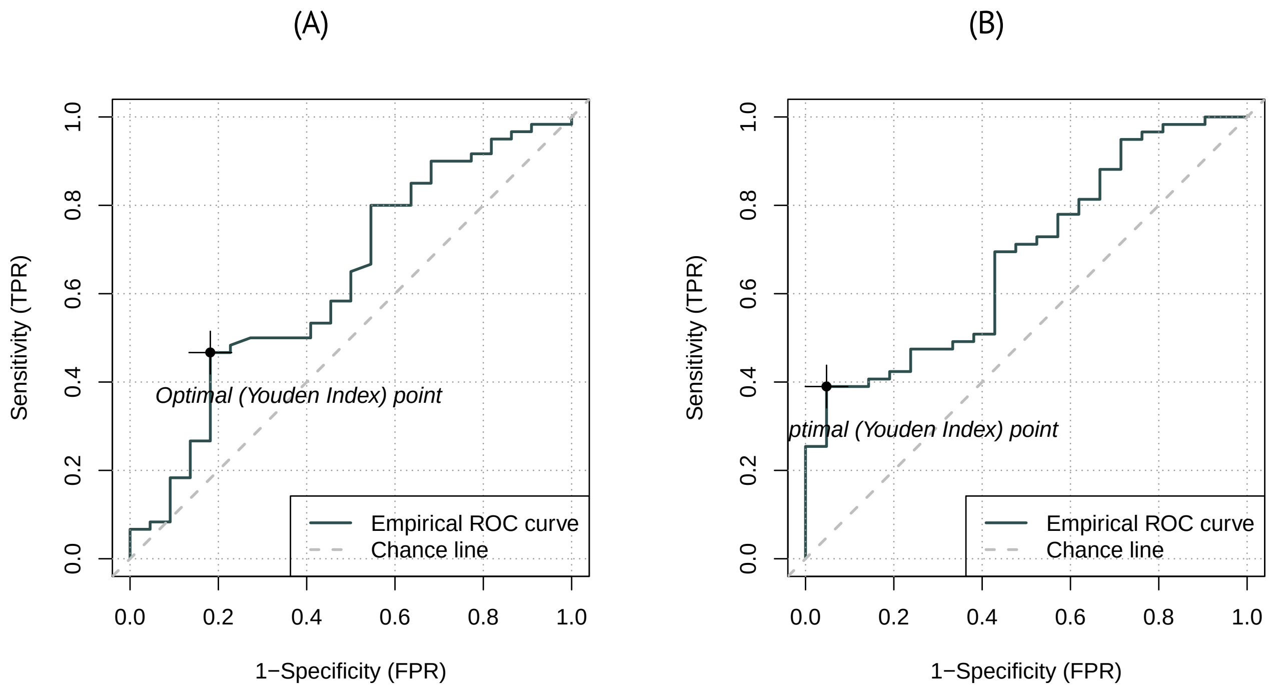

Figure S42. Receiver operating characteristics analysis with optimal point for the 55th percentile apparent diffusion coefficients in diagnosing congenital heart disease, comparing single- (A) and multi-slice (B) diffusion weighted imaging.

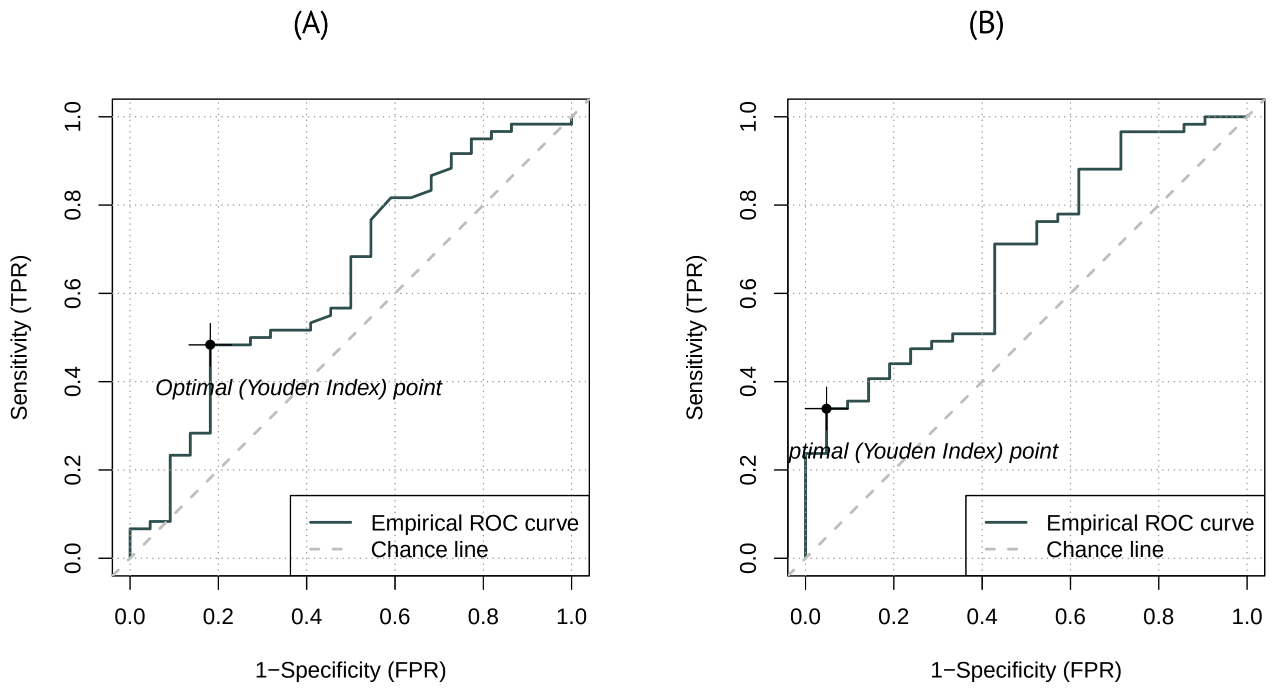

Figure S43. Receiver operating characteristics analysis with optimal point for the 60th percentile apparent diffusion coefficients in diagnosing congenital heart disease, comparing single- (A) and multi-slice (B) diffusion weighted imaging.

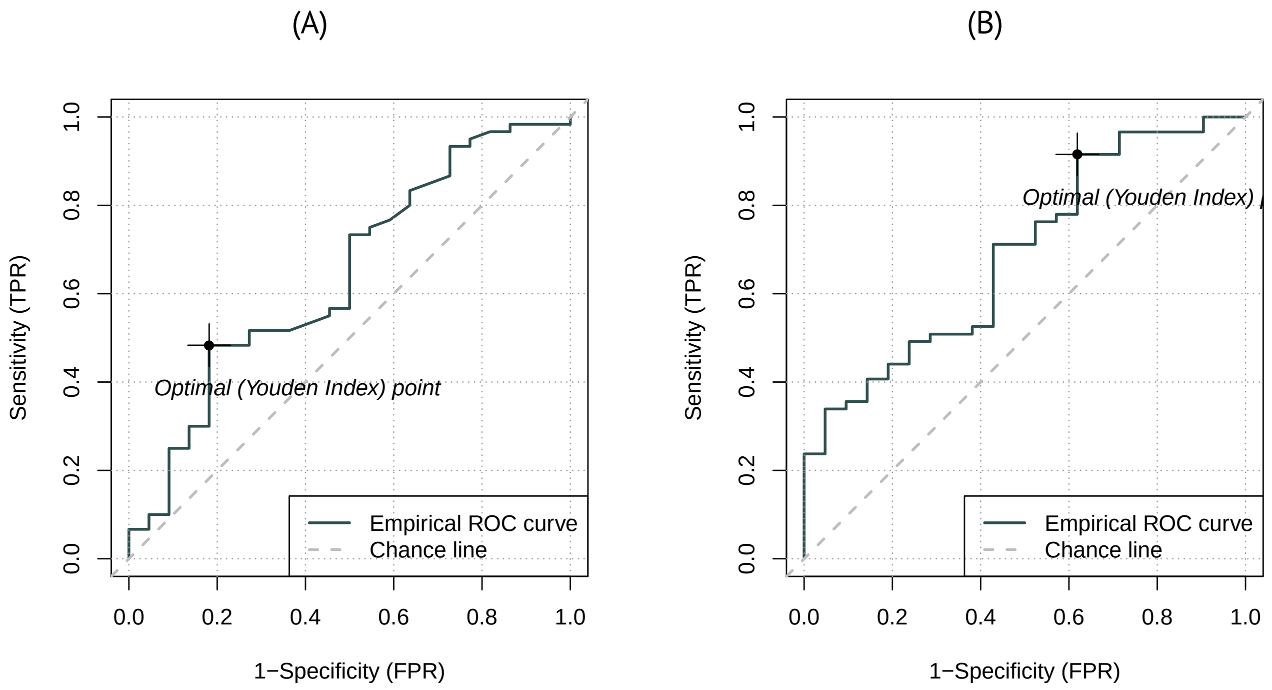

Figure S44. Receiver operating characteristics analysis with optimal point for the 65th percentile apparent diffusion coefficients in diagnosing congenital heart disease, comparing single- (A) and multi-slice (B) diffusion weighted imaging.

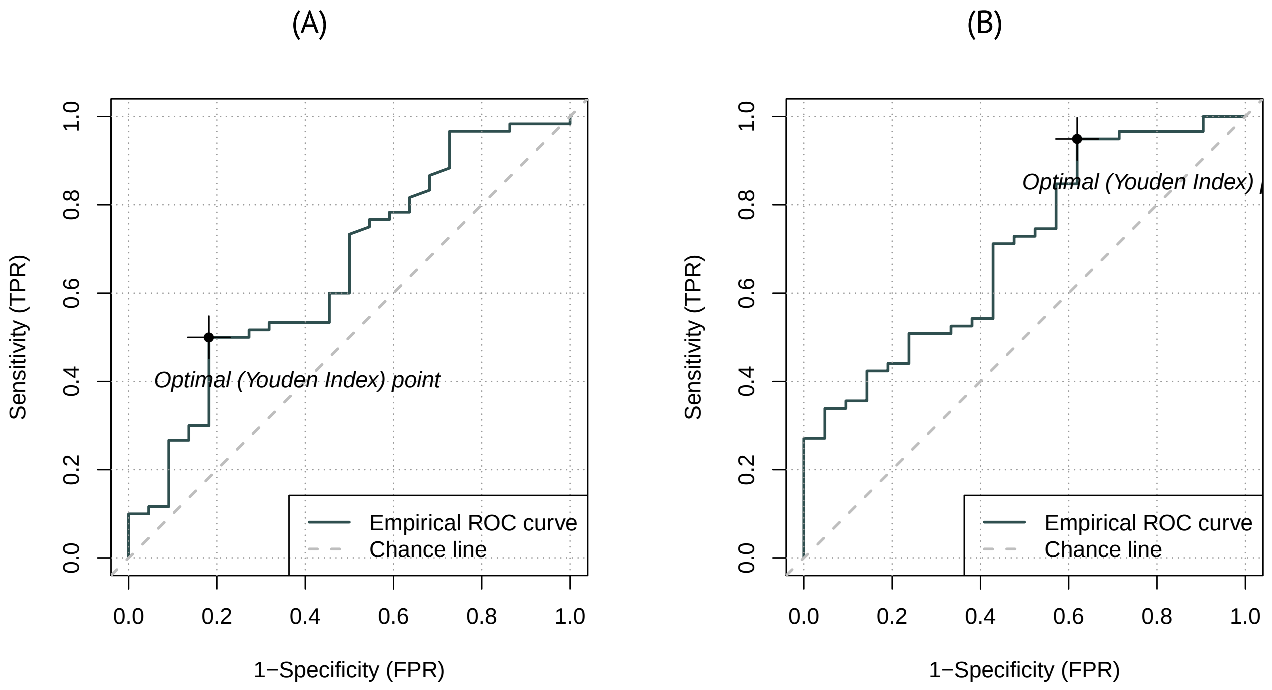

Figure S45. Receiver operating characteristics analysis with optimal point for the 70th percentile apparent diffusion coefficients in diagnosing congenital heart disease, comparing single- (A) and multi-slice (B) diffusion weighted imaging.

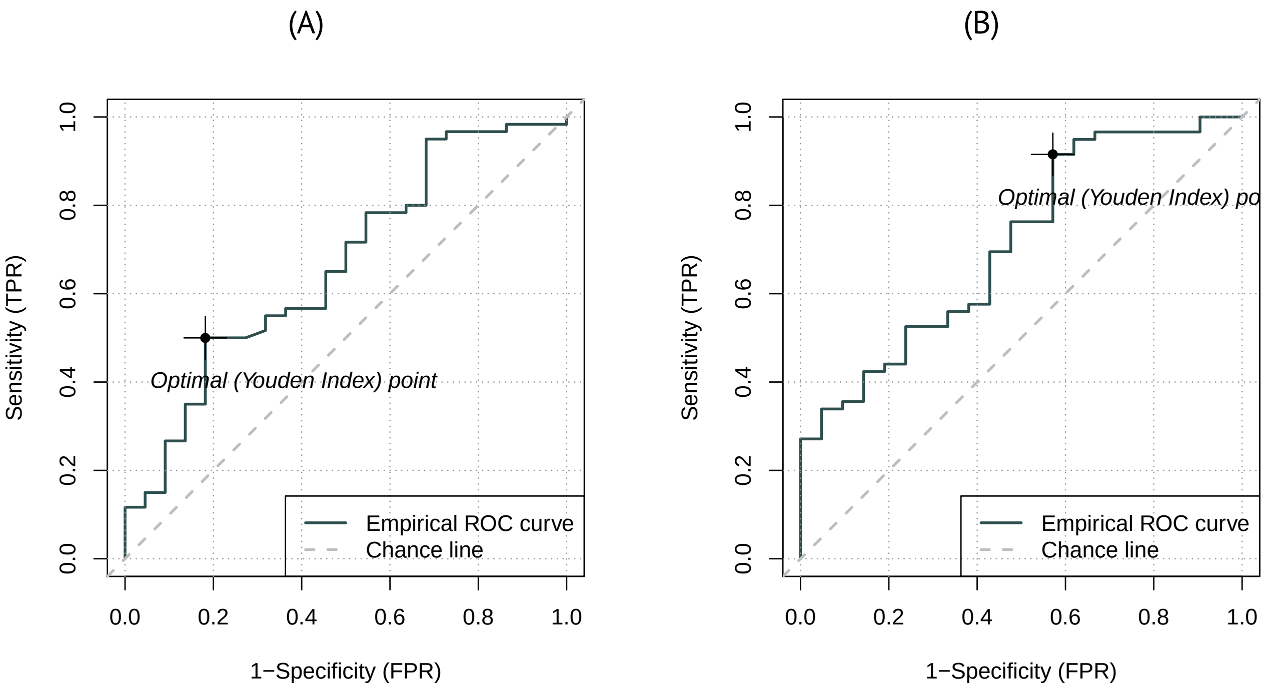

Figure S46. Receiver operating characteristics analysis with optimal point for the 75th percentile apparent diffusion coefficients in diagnosing congenital heart disease, comparing single- (A) and multi-slice (B) diffusion weighted imaging.

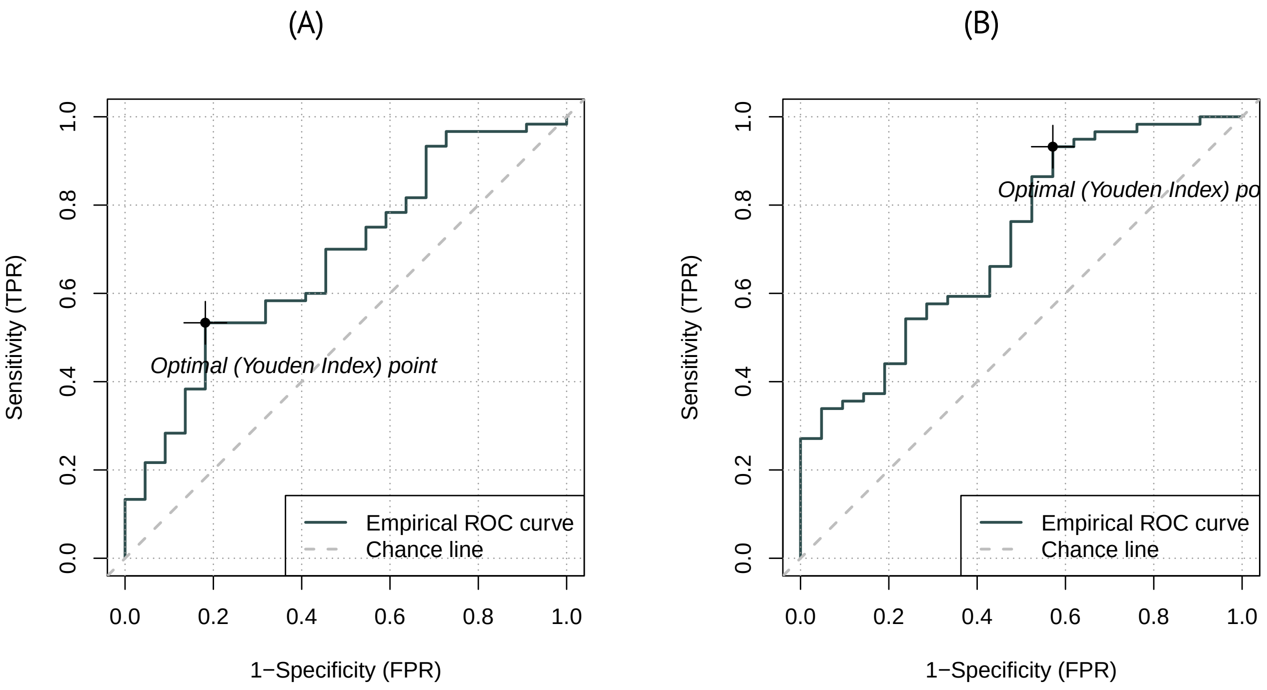

Figure S47. Receiver operating characteristics analysis with optimal point for the 80th percentile apparent diffusion coefficients in diagnosing congenital heart disease, comparing single- (A) and multi-slice (B) diffusion weighted imaging.

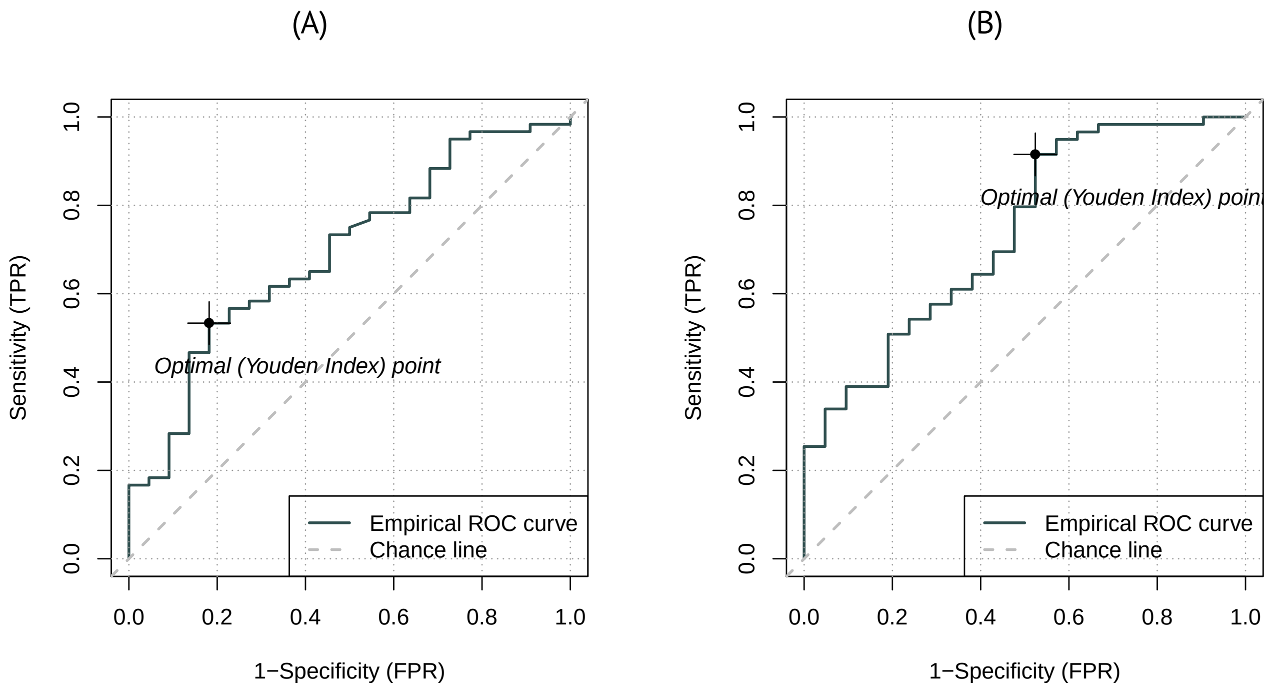

Figure S48. Receiver operating characteristics analysis with optimal point for the 85th percentile apparent diffusion coefficients in diagnosing congenital heart disease, comparing single- (A) and multi-slice (B) diffusion weighted imaging.

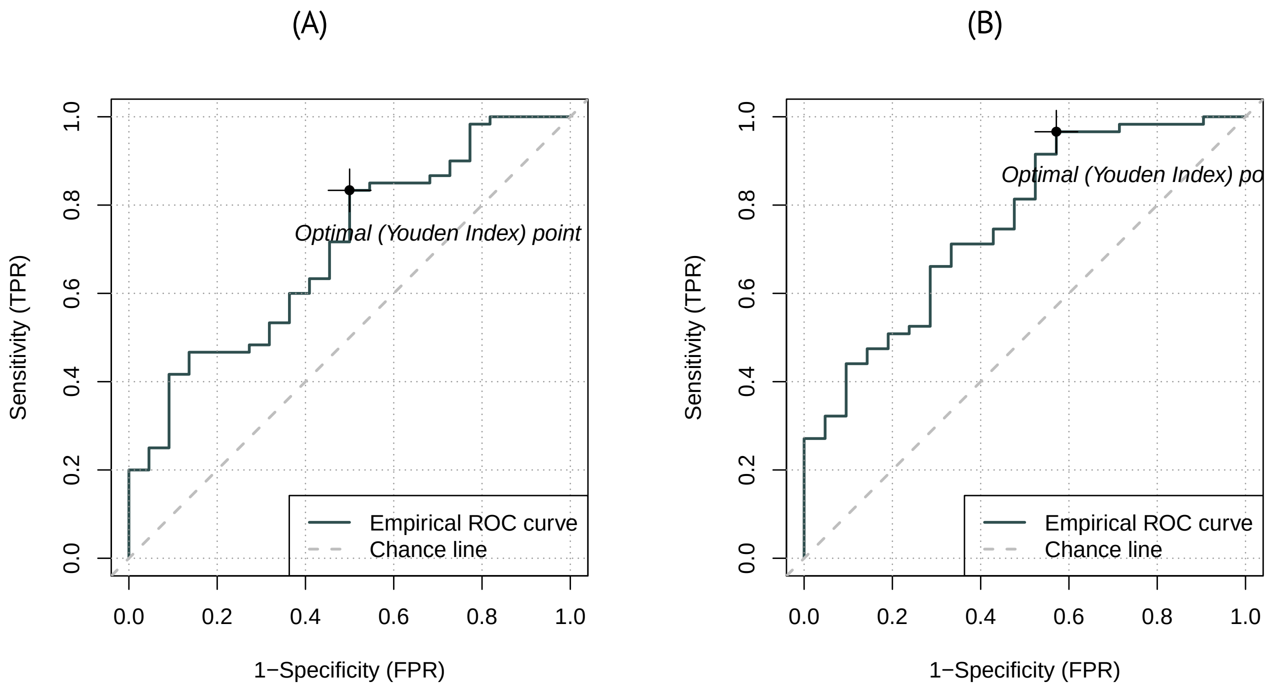

Figure S49. Receiver operating characteristics analysis with optimal point for the 90th percentile apparent diffusion coefficients in diagnosing congenital heart disease, comparing single- (A) and multi-slice (B) diffusion weighted imaging.

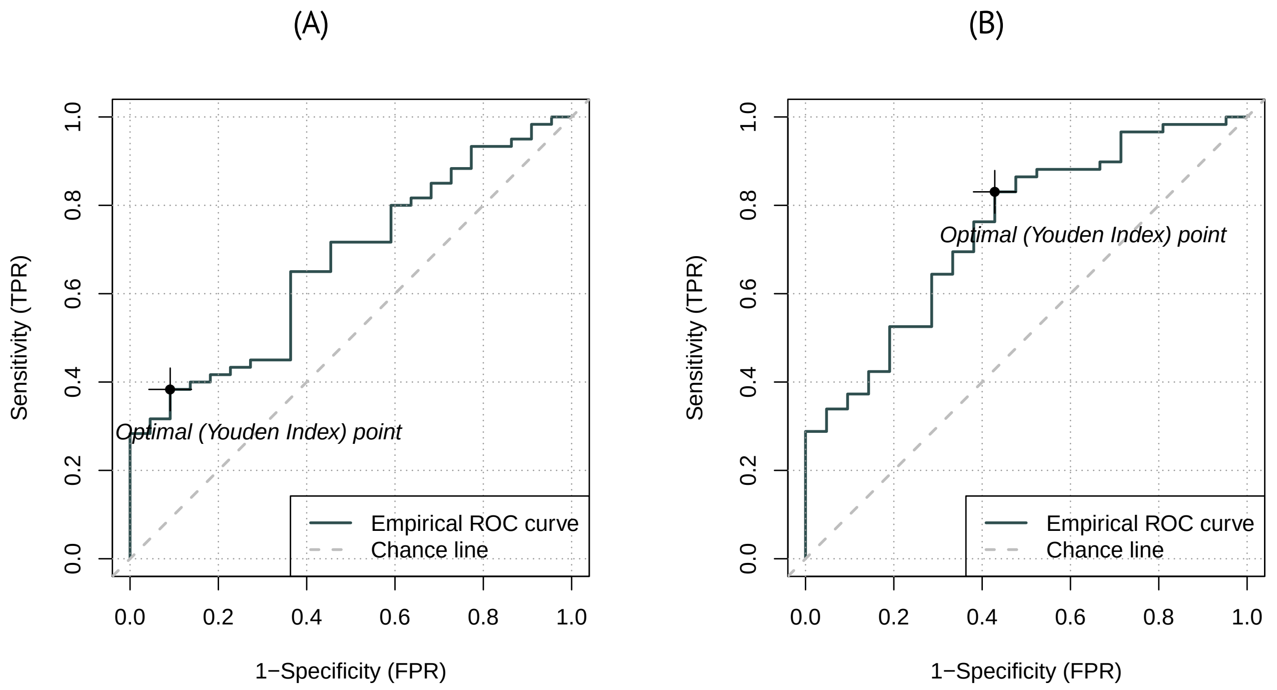

Figure S50. Receiver operating characteristics analysis with optimal point for the 95th percentile apparent diffusion coefficients in diagnosing congenital heart disease, comparing single- (A) and multi-slice (B) diffusion weighted imaging.

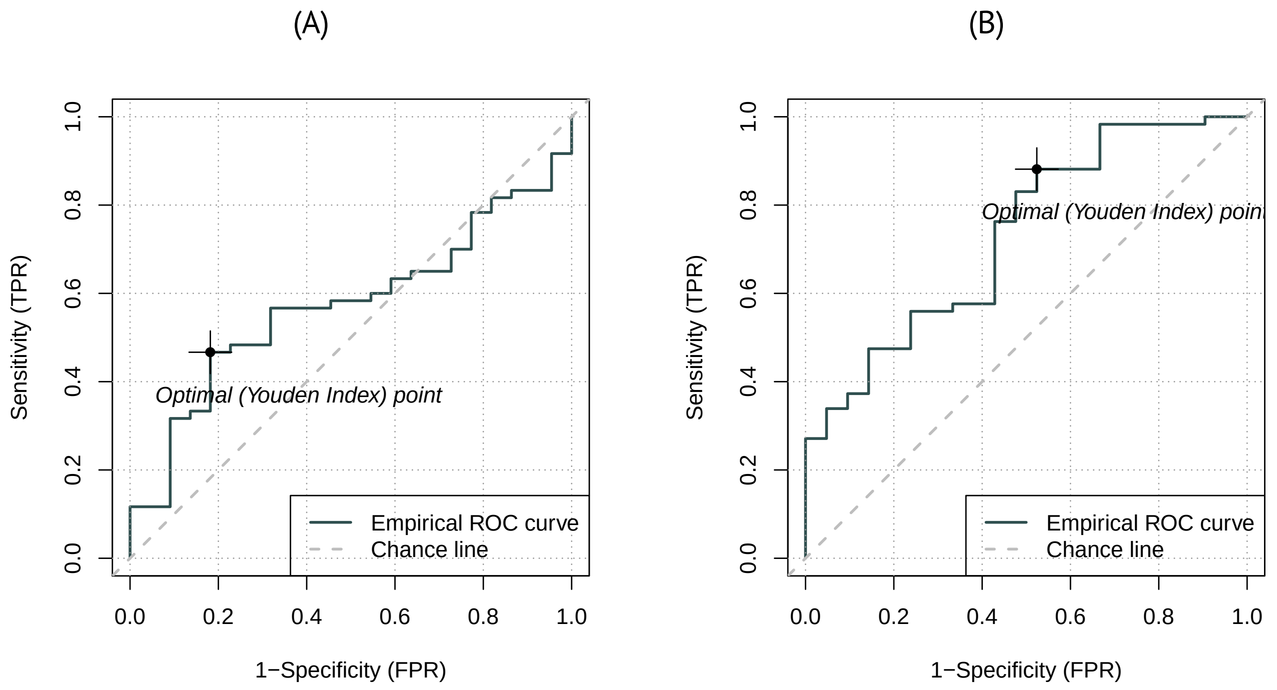

Figure S51. Receiver operating characteristics analysis with optimal point for the mean of apparent diffusion coefficients in diagnosing congenital heart disease, comparing single- (A) and multi-slice (B) diffusion weighted imaging.

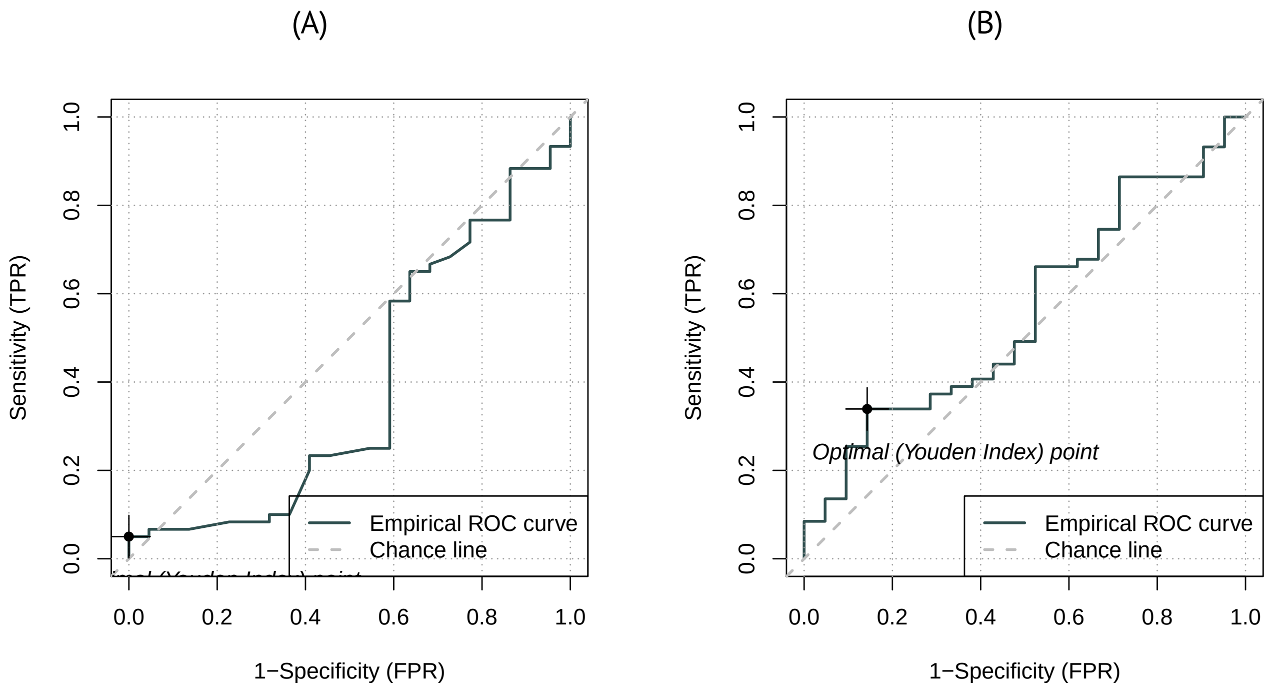

Figure S52. Receiver operating characteristics analysis with optimal point for the minimum of apparent diffusion coefficients in diagnosing congenital heart disease, comparing single- (A) and multi-slice (B) diffusion weighted imaging.

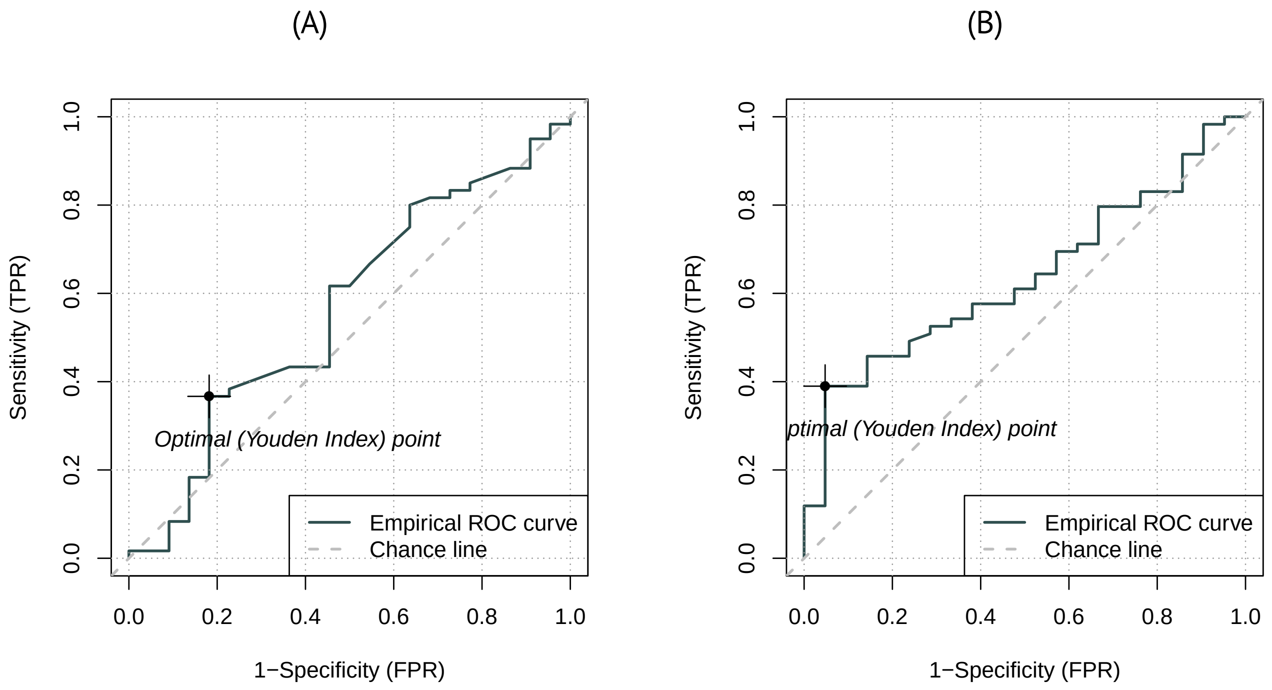

Figure S53. Receiver operating characteristics analysis with optimal point for the maximum of apparent diffusion coefficients in diagnosing congenital heart disease, comparing single- (A) and multi-slice (B) diffusion weighted imaging.

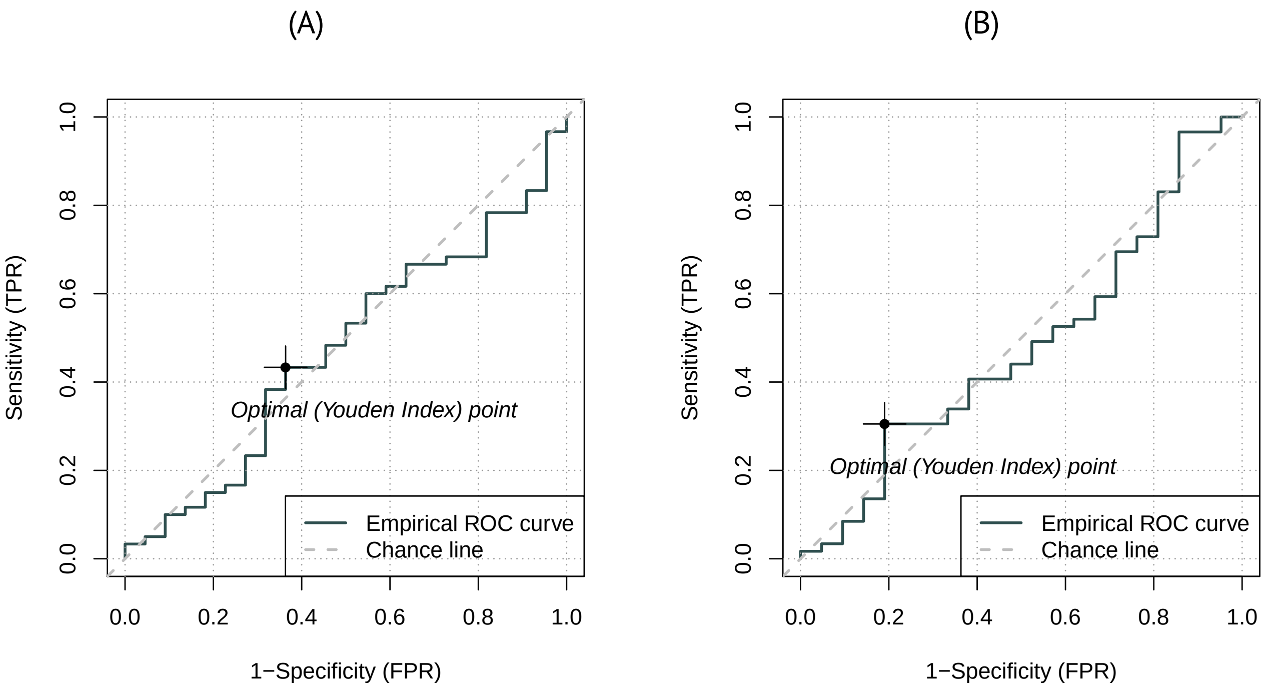

Figure S54. Receiver operating characteristics analysis with optimal point for the angular second moment in diagnosing congenital heart disease, comparing single- (A) and multi-slice (B) diffusion weighted imaging.

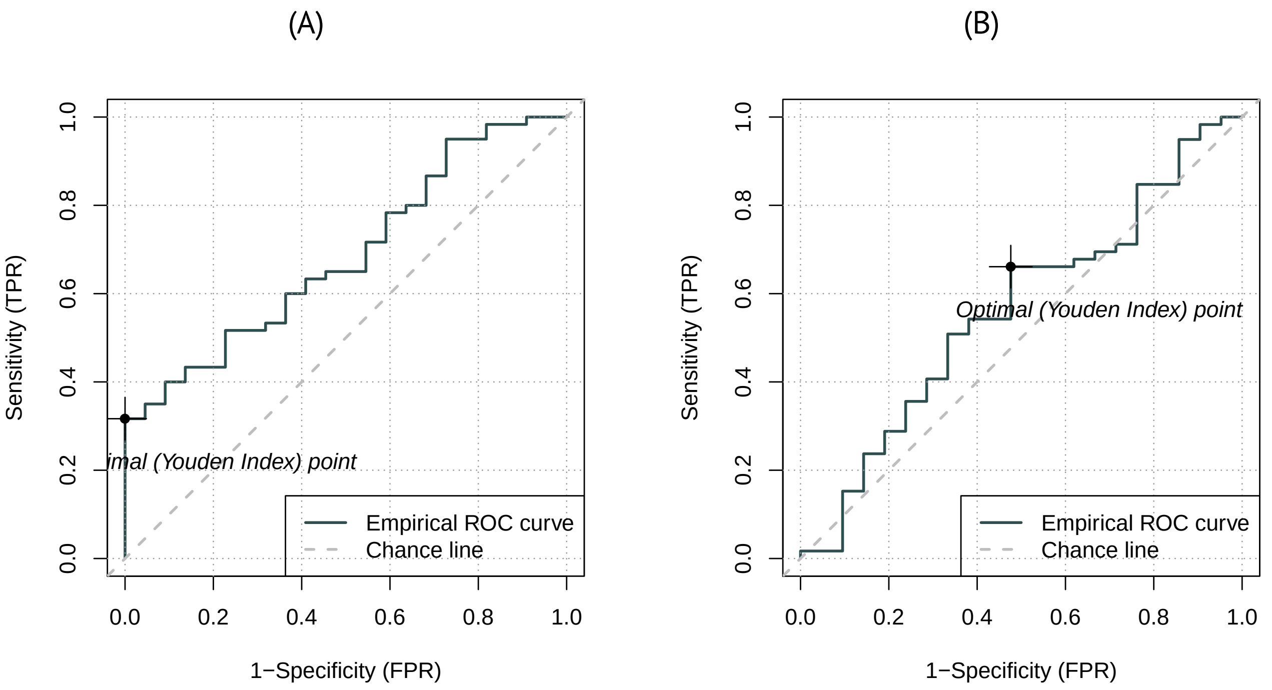

Figure S55. Receiver operating characteristics analysis with optimal point for the contrast in diagnosing congenital heart disease, comparing single- (A) and multi-slice (B) diffusion weighted imaging.

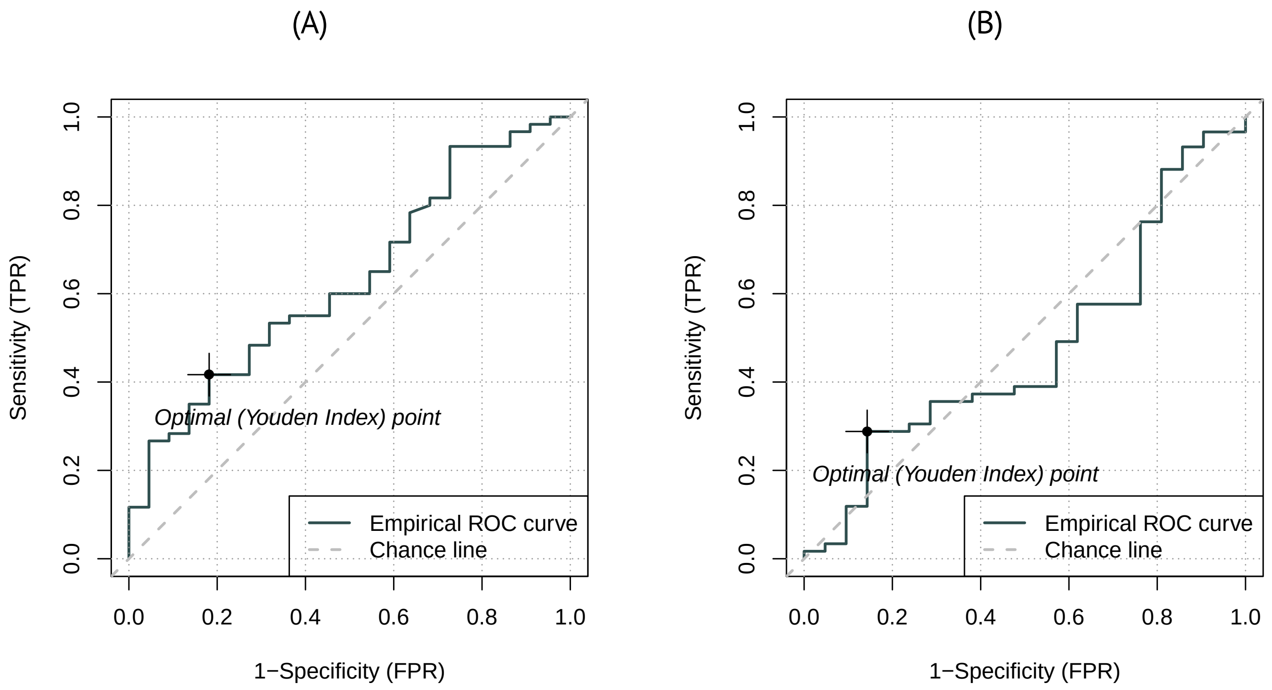

Figure S56. Receiver operating characteristics analysis with optimal point for the dissimilarity in diagnosing congenital heart disease, comparing single- (A) and multi-slice (B) diffusion weighted imaging.

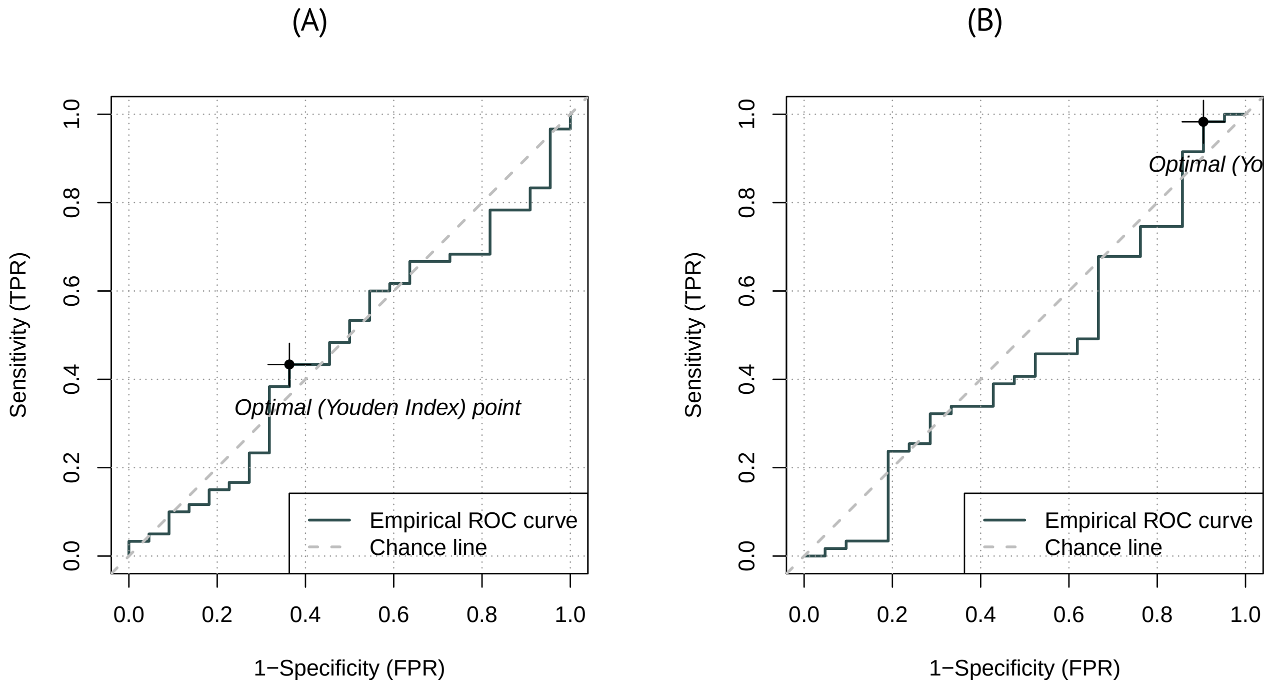

Figure S57. Receiver operating characteristics analysis with optimal point for the energy in diagnosing congenital heart disease, comparing single- (A) and multi-slice (B) diffusion weighted imaging.

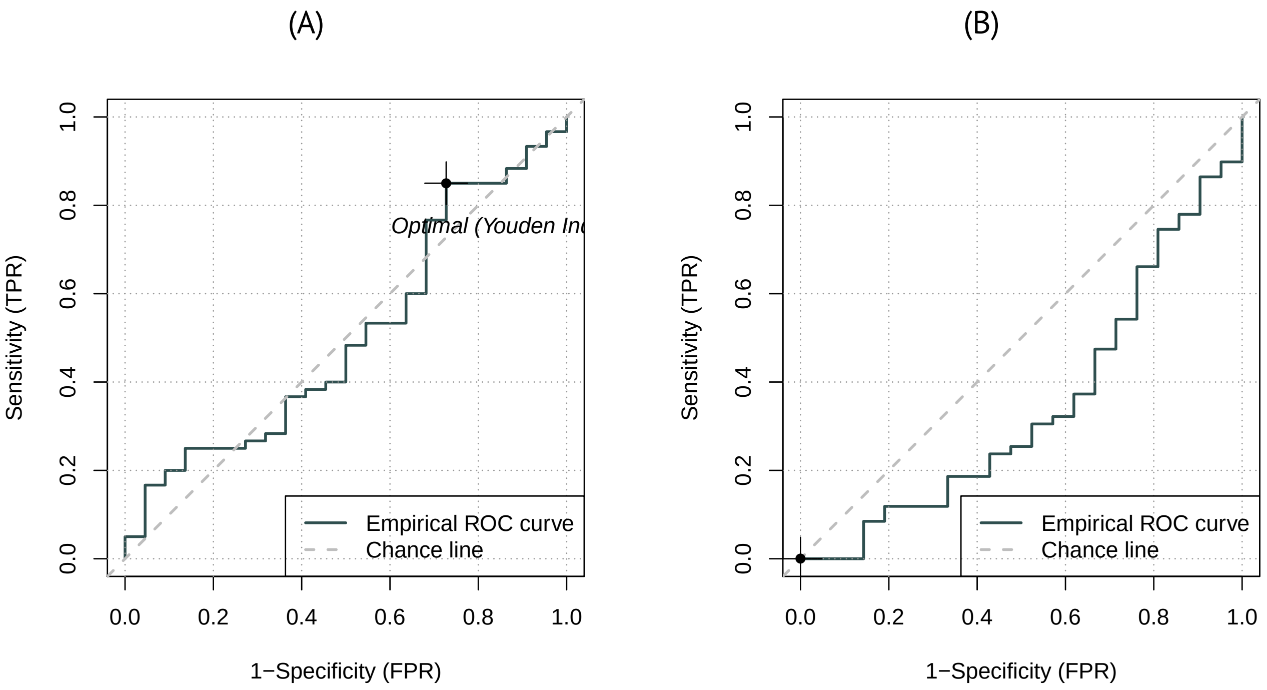

Figure S58. Receiver operating characteristics analysis with optimal point for the entropy in diagnosing congenital heart disease, comparing single- (A) and multi-slice (B) diffusion weighted imaging.

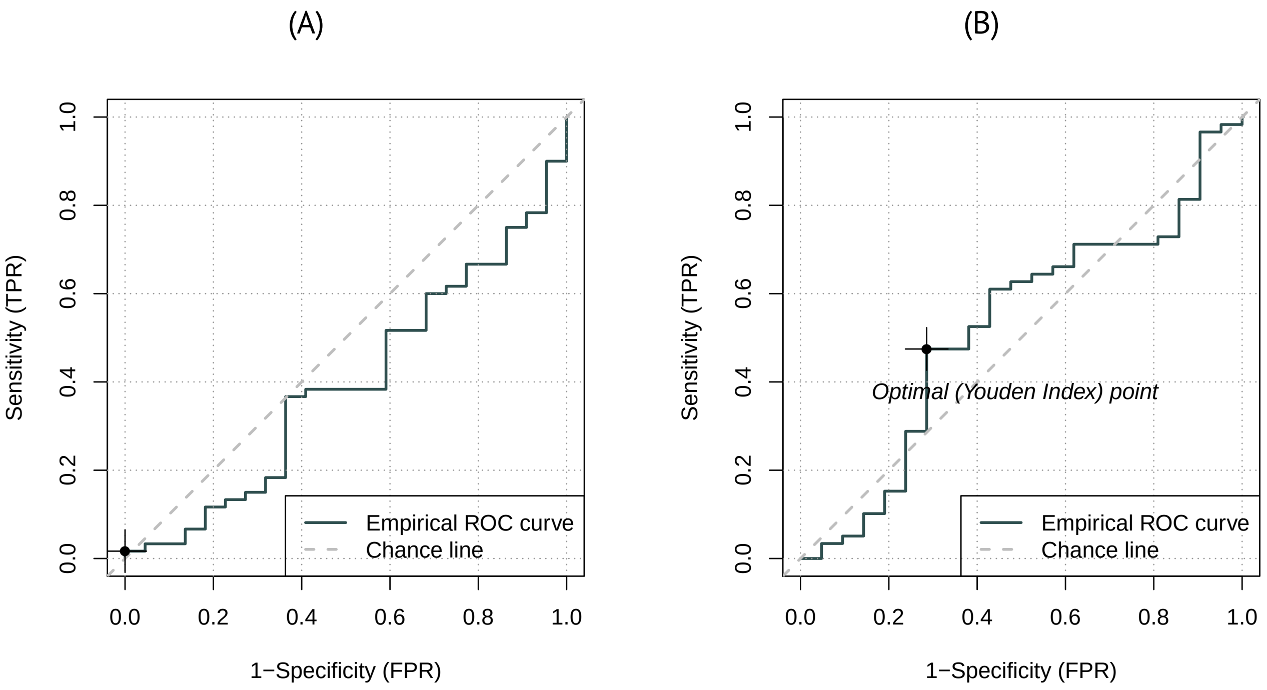

Figure S59. Receiver operating characteristics analysis with optimal point for the homogeneity in diagnosing congenital heart disease, comparing single- (A) and multi-slice (B) diffusion weighted imaging.

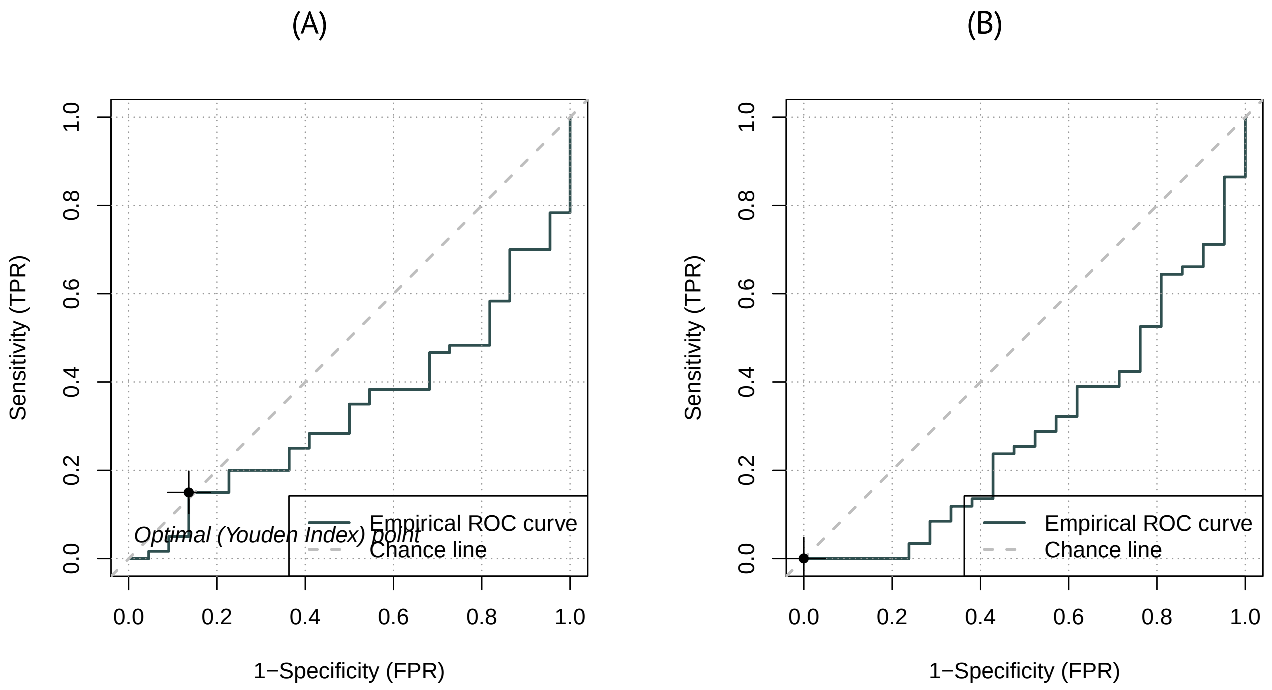

Figure S60. Receiver operating characteristics analysis with optimal point for the kurtosis in diagnosing congenital heart disease, comparing single- (A) and multi-slice (B) diffusion weighted imaging.

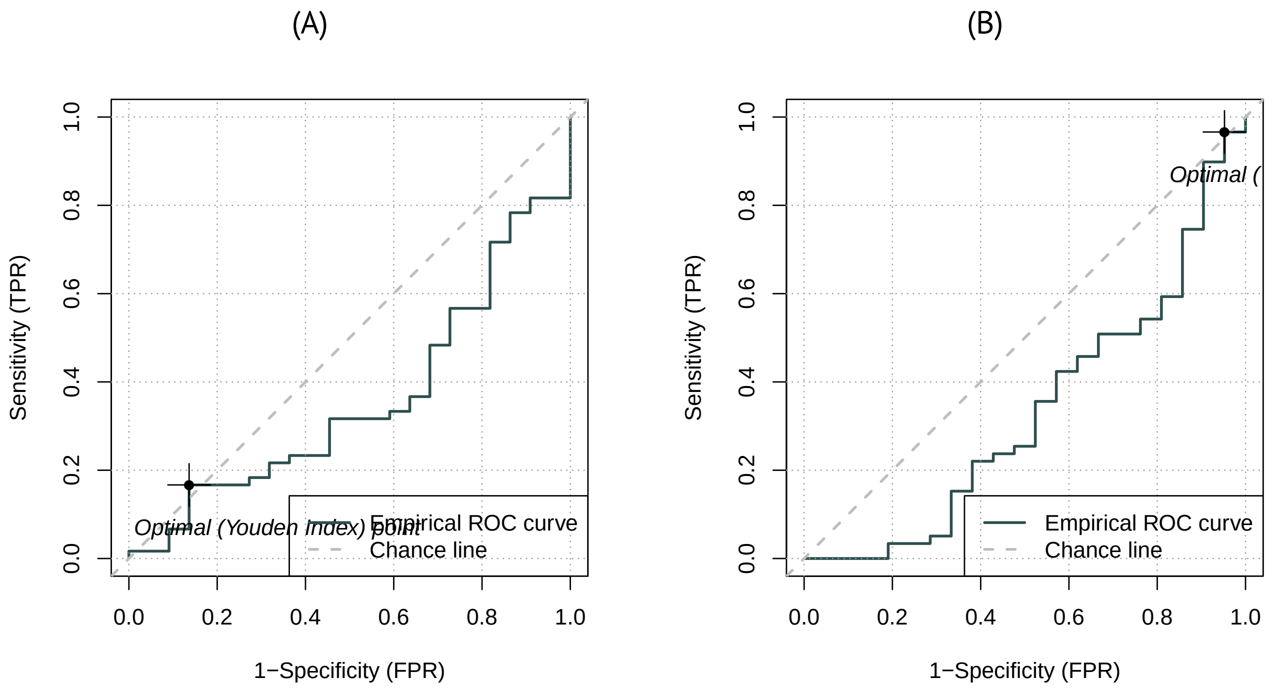

Figure S61. Receiver operating characteristics analysis with optimal point for the skewness in diagnosing congenital heart disease, comparing single- (A) and multi-slice (B) diffusion weighted imaging.

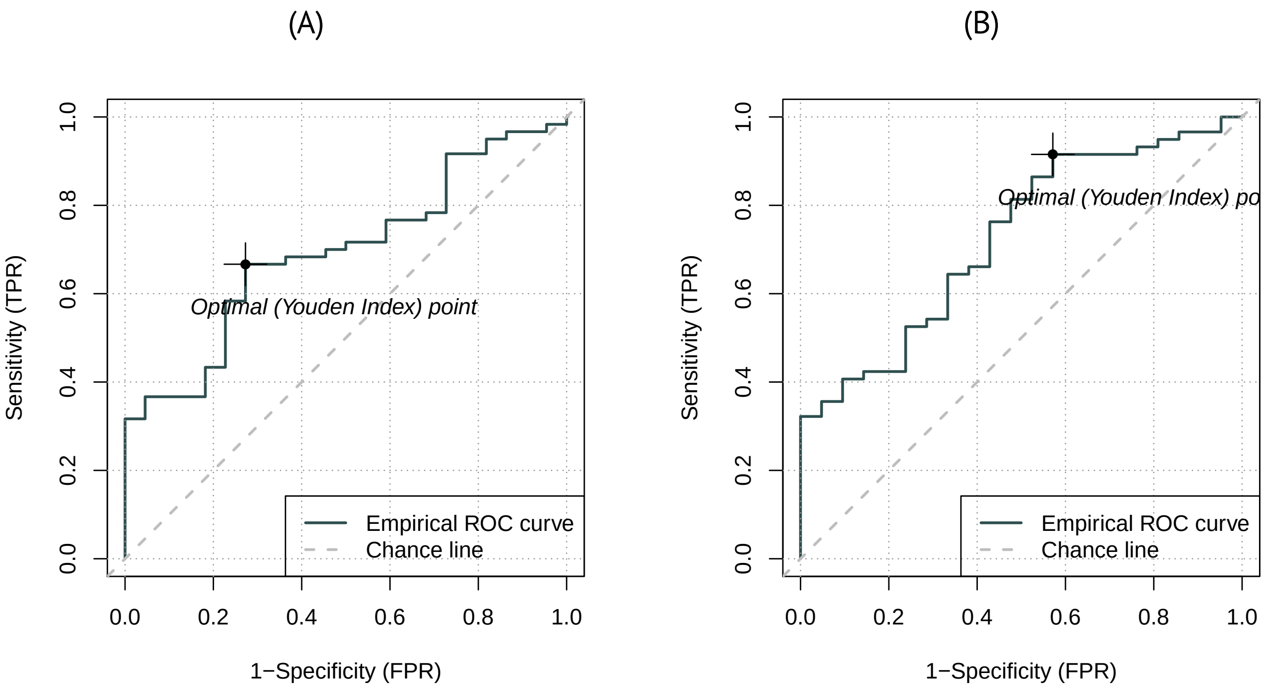

Figure S62. Receiver operating characteristics analysis with optimal point for the variance in diagnosing congenital heart disease, comparing single- (A) and multi-slice (B) diffusion weighted imaging.
